# Meta-Analysis of Carbohydrate Solution Intake during Prolonged Exercise in Adults: From the Last 45+ Years’ Perspective

**DOI:** 10.3390/nu13124223

**Published:** 2021-11-24

**Authors:** Dimitrios I. Bourdas, Athanasios Souglis, Emmanouil D. Zacharakis, Nickos D. Geladas, Antonios K. Travlos

**Affiliations:** 1 Section of Sport Medicine & Biology of Exercise, School of Physical Education and Sports Science, National and Kapodistrian University of Athens, 41 Ethnikis Antistasis, 17237 Athens, Greece; ngeladas@phed.uoa.gr; 2 Section of Didactics and Coaching in Sport Games, School of Physical Education & Sport Science, National and Kapodistrian University of Athens, 41 Ethnikis Antistasis, 17237 Athens, Greece; asouglis@phed.uoa.gr (A.S.); emzach@phed.uoa.gr (E.D.Z.); 3 Department of Sports Organization and Management, Faculty of Human Movement and Quality of Life Sciences, University of Peloponnese, Efstathiou and Stamatikis Valioti & Plataion Avenue, 23100 Tripoli, Greece; atravlos@uop.gr

**Keywords:** endurance, performance, systematic review, scientific quality, continuous, intermittent

## Abstract

Carbohydrate (CHO) supplementation during prolonged exercise postpones fatigue. However, the optimum administration timing, dosage, type of CHO intake, and possible interaction of the ergogenic effect with athletes’ cardiorespiratory fitness (CRF) are not clear. Ninety-six studies (from relevant databases based on predefined eligibility criteria) were selected for meta-analysis to investigate the acute effect of ≤20% CHO solutions on prolonged exercise performance. The between-subject standardized mean difference [SMD = ([mean post-value treatment group–mean post-value control group]/pooled variance)] was assessed. Overall, SMD [95% CI] of 0.43 [0.35, 0.51] was significant (*p* < 0.001). Subgroup analysis showed that SMD was reduced as the subjects’ CRF level increased, with a 6–8% CHO solution composed of GL:FRU improving performance (exercise: 1–4 h); administration during the event led to a superior performance compared to administration before the exercise, with a 6–8% single-source CHO solution increasing performance in intermittent and ‘stop and start’ sports and an ~6% CHO solution appearing beneficial for 45–60 min exercises, but there were no significant differences between subjects’ gender and age groups, varied CHO concentrations, doses, or types in the effect measurement. The evidence found was sound enough to support the hypothesis that CHO solutions, when ingested during endurance exercise, have ergogenic action and a possible crossover interaction with the subject’s CRF.

## 1. Introduction

Interest in the role and contribution of carbohydrates (CHOs) as an energy fuel, particularly during endurance exercise, dates back to the beginning of the 20th century [1]. Christensen and Hansen (1939) examined the role of a high-CHO diet and suggested that hypoglycemia causes fatigue during light exercise by affecting the central nervous system (CNS) [2]. In the late 1960s, it was revealed that exercise with glycogen depletion increases the resynthesis of muscle glycogen [3] and also that stored muscle glycogen plays a significant role during exercise [4]. Muscle glycogen stores are primly defined by diet prior to exercise. It has also been shown that the higher the muscle glycogen content, the higher the endurance performance [4,5]. In 1975, a study revealed that CHO feeding during prolonged exercise could increase exercise capacity, which was confirmed by another study in 1983 [5,6]. As scientific interest in the field of dietary supplements gradually grew, during the past 45 years, a great number of researchers have extensively investigated the effects of CHO consumption during endurance exercise, mostly from the perspective of determining the optimal composition and timing of CHO replacement beverages during exercise. Thus, the effect of CHO ingestion during endurance exercise has been reviewed by a great number of authors in the past and also in recent years [7,8,9,10,11], focusing on different areas of the CHO effect on performance.

It is widely accepted that endurance exercise requires a sufficient exogenous amount of CHO to postpone the onset of fatigue; when the CHO quantity is inadequate, performance is impaired [9,10,12]. However, many theories are still the subject of debate, and the conclusions of the relevant reviews have led to conflicting information about the optimum administration timing, dosage, type, and composition of CHO supplements [7,8,9,10,11]. For instance, four modern reviews recommend CHO intake up to 60 g·h^−1^ for exercise lasting up to 2.5 h and up to 90 g·h^−1^ when the duration of exercise exceeds 2.5 h [7,8,9]. Nevertheless, Mata et al. (2019) concluded that it is unclear which concentration (6, 8, or 10% etc.) or dose of CHO solution and which CHO substance (maltodextrin (MD), glucose (GL), sucrose (SUC), or a combination) enhance endurance performance better [10]. They also mentioned that “attending to the existing evidence, no universal recommendations regarding CHO intake should suggested to athletes” [10]. In a simplified approach, Brooks (2020) states that as gastrointestinal (GI) emptying and absorption are determinant factors of exogenous glucose availability, beverages containing 4−6% GL could be efficient during exercise for euglycemia maintenance, while GL solutions > 6% are often less effective and blamed for GI discomfort [11]. It is understood, however, that the studies varied in method, exercise duration, performance assessment (e.g., capacity: time to exhaustion vs. performance: fix distance), and total quantity of CHO that was administered, which may explain any inconsistency in the literature.

Unexpectedly, no systematic review or meta-analysis has taken into account the possible effect of the cardio-respiratory fitness (CRF) of the tested subjects on the CHO intake intervention. An extensive comparative and updated review on the effect of CHO supplements on different types of exercise, such as cycling vs. running, also appears to be lacking. Thermal stress-induced energy metabolic changes during prolonged exercise of different modes and intensities are likely to be different [13,14]; yet, the ergogenic role of CHO supplementation in different ambient conditions has not been reviewed in a meta-analysis. Additionally, with only a few exceptions, most past reviews more or less failed to mention how the search for relevant studies was carried out, what the inclusion criteria were for the studies and whether they were appropriate, whether the validity of included studies was assessed, whether the methods and statistics were reliable and appropriate, how conclusions were reached, whether results were explicit, and how the studies were integrated, which can elicit overestimation or underestimation of the intervention effect [15]. No meta-analysis has examined the effect of CHO ingestion with no additives (e.g., caffeine, protein) during endurance exercise on endurance capacity and performance, with the use of an accurate method of combining the results of independent studies, assessing risk-of-bias, and considering the potential limitations of the eligible studies [15].

Therefore, the purpose of this study was to select relevant papers from a specific period (1975–2021) for a systematic/critical review using a meta-analytic technique. It is certain that athletes, coaches, and training instructors will be interested in seeing the actual CHO ergogenic supplementations’ effect on different exercise modalities in association with supplement composition, concentration, administration time, and exercise duration; a systematic review conducted according to predefined methodological criteria will surely be beneficial to this audience. Therefore, the aim of this study was to investigate whether the literature supports the hypothesis that CHO supplementation in a liquid form during exercise enhances performance, taking into account the subjects’ CRF and the relative methodological quality of the papers searched. A further aim was to establish the optimum administration time and the optimal composition and concentration of CHO replacement during endurance exercise, and to contribute to resolving the controversy posed by the previous reviewers’ relevant conflicting findings. On the other hand, this study focused mainly on the scientific evidence for the efficacy of CHO supplementation rather than on understanding the mechanism/s involved.

## 2. Materials and Methods

### 2.1. Search Strategy

**Figure 1:**
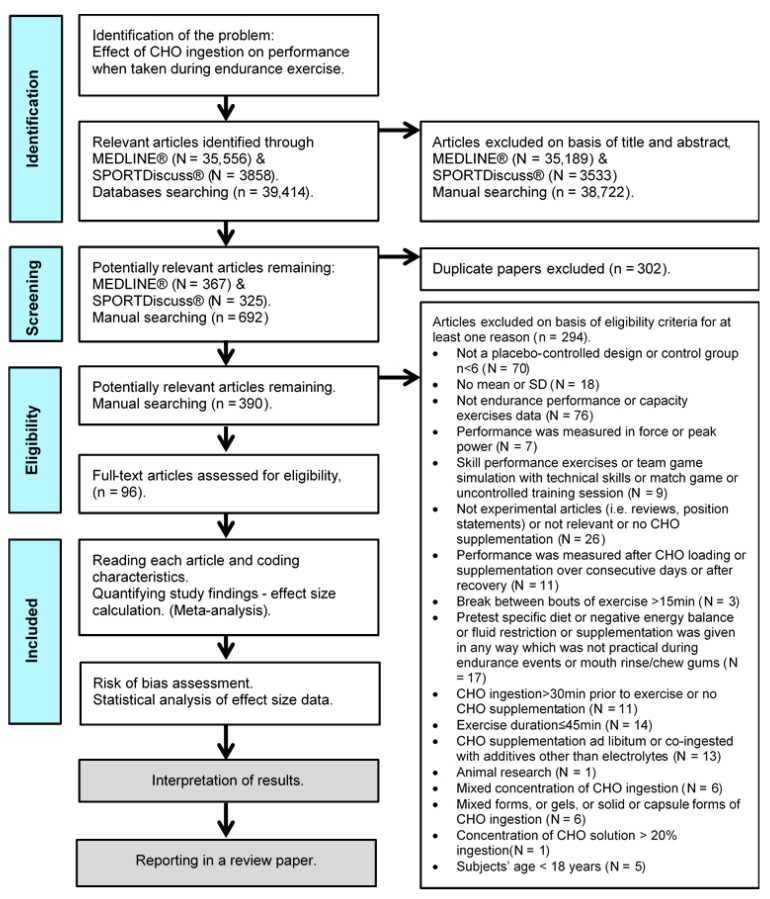
Prisma flowchart of the study selection process [16].

For the purposes of the present systematic review, a meta-analysis was conducted, based on the Preferred Reporting Items for Systemic Reviews and Meta-Analyses (PRISMA) and Cochrane Handbook for Systematic Reviews of Interventions statements guidelines [16,17]. Figure 1 outlines a summary of the procedures followed in this study.

### 2.2. Databases and Search

The initial electronic literature review was carried out by searching the MEDLINE® (via PubMed^®^), and SPORTDiscus^®^ (via EBSCOhost) databases until April 2021. We restricted our review to studies published since 1975, which, to our knowledge, was the year of publication of the first study showing that CHO feeding during prolonged exercise improves exercise capacity [5]. We did not review any study dating from August 2021 to the article submission day. We used the Boolean search syntax ((carbohydrate* OR CHO*) AND (endurance OR performance OR capacity OR exercise OR timing)). Full-text studies chosen were limited to those published in English peer-reviewed journals, with human subjects used. Additionally, control clinical trial OR/AND randomized clinical trial filters were activated, and a total of 36,605 studies from MEDLINE® and 3956 studies from SPORTDiscus® were found.

### 2.3. Eligibility Criteria

From the preliminary articles originally identified through the search of the electronic databases, all irrelevant articles were manually excluded based on their title and abstract. A number of studies were manually picked through predefined eligibility criteria by (two independent: E.Z. and G.M.) reviewers, who screened the potentially relevant papers by checking their titles, abstracts, methods, and results. In order to avoid risk of bias in selecting and rejecting papers, reviewers looked first at methods and then at the results.

The independent variables were: (a) the contents (e.g., GL, FRU) of a liquid form of supplementation only (i.e., CHO ≤ 20%, e.g., solution, drink, beverage); (b) the CHO concentration (%) or the dose (i.e., ingestion rate (CHO g·h^−1^)); and (c) the timing of CHO supplement ingestion during an endurance exercise (at regular intervals or single bolus dose), which was not less than 30 min before the beginning of the event until its end. The dependent variable was defined as the effect of CHO supplementation on endurance performance, lasting ≥ 45 min (time to exhaustion or time to complete a certain distance in events of variable duration and intensity). The rationale for initially restricting attention to exercise duration effort ≥ 45 min was that endogenous muscle glycogen is not fully depleted in 1-h all-out exercises [18]. During an exercise (>85%

$$\dot{V}$$

O_2_max) performed continuously for 20–30 min, fatigue in skeletal muscle is caused by an increased accumulation of H⁺ [19]. Thirdly, the ergogenic influence of a CHO solution on an intense exercise of a relatively short duration may be partially explained by the solution’s stimulus on the brain via mouth receptors sensitive to CHO; thus, the ergogenic effect may be not exclusively metabolic in nature but could also be attributed to the CNS [7].

Only papers of controlled interventions, where the authors reported that they used a specific experimental method, were chosen. When an article contained more than one research arm that qualified for inclusion, they were regarded as separate ‘interventions’ denoted as ‘trials’. In particular, studies or trials that did not involve a comparison group in a parallel or crossover design [20], single-subject design studies, studies that used sample sizes (N) less than six subjects per group, and studies that did not provide the numerical means (not depicted) and standard deviation (SD) or standard error (SE = SD divided by the square root of the sample size) for the dependent variable were not included. Editorials, letters to the editor, government’s reports, grey literature, or abstracts or scientific events or other articles indexed by non-scientific databases (not peer-reviewed) that did not contain original results were also excluded from this review. Original studies that reported the use of healthy human subjects (age ≥ 18 years) and were relevant to the topic of interest, i.e., where appropriate, independent and dependent variables could be defined from the article’s title and abstract, were included.

Additionally excluded were studies with substantial rest intervals (>15 min), or CHO supplementation given during recovery from exercise, during team games, during uncontrolled training sessions, during an exercise protocol that included technical sport drills (e.g., ball drills) designed to simulate a fast-paced game, or supplementation administered in many different concentrations or given in solid (e.g., chocolate, energy bar, pudding), gel, capsule, or mixed forms (e.g., solution co-ingested with gel), intravenously, or in any other way, which was not practical during endurance events. Studies that used an intermittent exercise protocol designed to simulate a fast-paced game or ‘stop and start’ sports, using only running, jogging, and walking activities, were included. Studies that used CHO supplementation in combination with electrolytes were chosen when the control group was also provided. Studies that used CHO supplementation: ad libitum, co-ingested with additives (i.e., any neurological stimulant (e.g., caffeine), neurotransmitter or neuromodulator (e.g., taurine), NAD⁺ precursor (e.g., nicotinic acid), substances that may have synergetic effect or are advocated in the medical literature for muscle fatigue reduction after exercise or boosting metabolism (e.g., vitamins, chromium picolinate, carnitine), and potential energetic substrate (e.g., fat, protein) [21,22,23]), in fasted state (except overnight fasting) or in negative energy balance, after pre-experimental CHO preloading or any kind of enriched or specific diet other than normal were excluded. Studies that exclusively used a fluid restriction protocol (pre supplementation or during exercise) or a protocol to investigate during exercise the effects on the hormonal response, immune response, gastric emptying, GI problems, GL oxidation, heart rate, rate of perceived exertion, cognitive performance, reaction time, resistance exercise, peak power, velocity, force, torque, energy cost or technical skills, and CHO mouth rinse or CHO chewing gum response after treatment were also excluded. Finally, after the removal of duplicate articles, 294 articles were excluded and 96 selected for further analysis (Figure 1).

### 2.4. Data Extraction

The next step was to code the characteristics and outcomes of the selected studies that were likely to influence the true intervention effect sizes [17]. Characteristics provided descriptive information about the study with the following categories: design (e.g., randomization, control/no control, statistical analysis…), protocol test (e.g., continuous, intermittent…), endurance exercise mode (e.g., cycling, running, swimming…), treatment variable (e.g., supplementation that was used, concentration, dose, administration time, composition, form…), subjects (e.g., gender, age, CRF, …), dependent variable (e.g., performance time, exhaustion time …), and environmental conditions (e.g., temperature, altitude …). No contact was made with the studies’ authors. Two reviewers (A.K. and K.P.) independently processed data extraction from the initially selected studies. Disagreements between reviewers with regard to including or excluding data of a given study were resolved by consensus.

### 2.5. Risk-of-Bias Assessment and Deficiencies in Scientific Design or Reporting

A modified version of the Cochrane risk-of-bias assessment tool for systematic reviews was employed to assess potential risk-of-bias in the eligible studies [24]. The modification was based on empirical evidence showing that they have a biasing effect on the estimates of a treatment’s effectiveness. This evidence derived from previous systematic reviews [25,26,27,28] and their importance to the reviewer in determining whether confidence should be placed on the author’s conclusions based on the CONsolidated Standards of Reporting Trials 2010 guidelines [29,30]. Nine risk-of-bias items were used for all eligible studies (i.e., eligibility criteria; statistical power calculation; subject’s familiarization; time series control of treatment allocation over the study period; subjects blinded to treatment; researchers blinded to treatment; reliability of measures; validity of measures; complication or dropout ≥ 15%), which were graded as low (+), some concerns (?), and high (−) risk-of-bias. Two external researchers (K.P. and A.K.), unaware of this study’s purpose and of any data that could help identify the studies’ authorship (e.g., authors’ names and affiliations, year, and type of publication), assessed the studies’ risk-of-bias based on answers to the signaling questions independently [24]. Any disagreement between the two researchers was resolved by discussion; if no consensus could be reached, a third researcher (T.T.) made the final decision.

### 2.6. Statistical Analyses

Data were analyzed using the Review Manager software, Version 5.3.4. (Cochrane Collaboration Copenhagen, The Nordic Cochrane Centre, Copenhagen, Denmark). The effect size (ES) of CHO supplementation on exercise performance was calculated as the between-subject standardized mean difference (SMD = ([mean post-value treatment group–mean post-value control group]/pooled variance)). Due to the nature of the test performance assessment, performance mean data were inserted into Review Manager software for analysis in a negative way (i.e., multiplied by −1) so that both studies (time-to-exhaustion and self-paced time-trial) corresponded to the same direction by means of the effect size in performance enhancement [32,33,34]. Studies with small sample sizes have a biased ES [35], and thus, each SMD was multiplied by a correction factor (g) to allow an unbiased estimate of ES [36,37,38]. The correction factor was calculated from the formula: g = [1 − 3/(4Ni-9)], where, Ni = pooled sample size. According to Higgins et al., (2011), SMD values of 0.2–0.4 indicate a small effect, 0.5–0.7 indicate a medium effect, and >0.8 indicate a large effect [15].

Furthermore, subgroup analysis was carried out to see if they had an advantage effect on their own and also to identify aspects of any possible study heterogeneity. Depending on the related published studies’ data, study trials were classified as follows: (a) with regard to the subjects’ characteristics, into three gender classes (male (M), female (F), combined (MF)), four age classes (young (18–29 years), adults I (30–39 years), adults II (40–49 years) [39]), and four CRF classes based on maximal oxygen uptake data (fair, good, excellent, superior) [40]; (b) with regard to exercise task, into three exercise mode classes (cycling, running, other (triathlon, duathlon, swimming, walking, loaded marching, roller-skiing)), two exercise protocol test classes (capacity: time to exhaustion, performance: time trial), three exercise type classes (intermittent: sessions interspersed with short rest or recovery periods involve activity of lower intensity; continuous: no break between sessions, regardless of the intensity of the sessions; intermittent shuttle: sessions that simulate the activity pattern of ‘stop and start’ sports), and four exercise time classes ((T), (45 min ≤ T ≤ 60 min, 60 min < T ≤ 120 min, 120 min < T ≤ 240 min, T > 240 min)); (c) with regard to supplementation, into seven CHO concentration classes (0% < CHO ≤ 2%, 2% < CHO ≤ 4%, 4% < CHO ≤ 6%, 6% < CHO ≤ 8%, 8% < CHO ≤ 10%, 10% < CHO ≤ 15%, 15% < CHO ≤ 20%), five CHO dose classes (CHO dose ≤ 40 g·h^−1^, 40 g·h^−1^ < CHO dose ≤ 60 g·h^−1^, 60 g·h^−1^ < CHO dose ≤ 80 g·h^−1^, 80 g·h^−1^ < CHO dose ≤ 100 g·h^−1^, CHO dose > 100 g·h^−1^), six CHO type classes (GL, MD, SUC, maltose (MAL), FRU, galactose (GAL)), 12 multiple transportable CHO classes ((MTC), (GL:FRU, GL:SUC, GL:MD, MD:FRU, MD:Dextrose (DEX), MD:SUC, GL:MD:FRU, GL:MD:DEX, GL:SUC:FRU, GL:MD:MAL:Saccharides, SUC:MD:IsoMAL, unclear CHO substances mixture), three CHO solution formulation classes (single-source CHO solution, double-source CHO solution, triple-or-more-source CHO solution), four administration time classes (prior to or at the beginning, during, prior to or at the beginning + during, late in exercise), and two supplement temperature administration classes (cool (<18 °C), neutral (18–26 °C)); and d) with regard to ambient conditions, into three thermal condition classes (cool (<18 °C), neutral (18–26 °C), heat (>26 °C)).

An assessment of the consistency of effects across eligible studies in the subgroups was also carried out. The between-study heterogeneity was assessed using the I² statistic and the Chi-square test. According to Higgins et al. (2003) and Sedgwick (2015), values of the I^2^ statistic of 0–50% represent low heterogeneity, 50–74% moderate heterogeneity, and ≥75% high heterogeneity [41,42]. It was assumed that subgrouped studies were characterized by a high degree of homogeneity due to similar clinical and methodological aspects. Consequently, SMD could be compared to find possible differences between treatments and controlled with a fixed-effect meta-analysis model (estimating the same underlying intervention effect) by means of the inverse-variance method when I^2^ < 50% [43]. However, the fixed-effects analysis may not be the proper option to account for the accessible random-effects within the analysis. On the other hand, irrespective of the I^2^ statistics, the random-effect meta-analysis model (which is usually used in case of I^2^ ≥ 50%) may provide a more conservative estimate that may be viewed as an ‘average intervention effect’ [44] and thus it was programmed to compute the pooled effect size by calculating the SMD in the current study. Nevertheless, the decision between fixed- and random-effects meta-analyses has been the subject of much debate and since many authors have argued that a fixed-effect analysis can be interpreted in the presence of heterogeneity, and that it makes fewer assumptions than a random-effects meta-analysis [45,46], Cochrane organization does not provide a universal recommendation [17]. For this reason, based on fixed-effects analysis assumptions [45,46] and Cochrane organization recommendations (that it may be reasonable to present both analyses) [17], we also ran fixed-effects analysis and report these results, only in two cases, where the presence of homogeneity (I^2^ < 50%) supported our actions and better interpreted our well-founded assumptions. So, the outcomes of this meta-analysis are derived from the random-effect meta-analysis model throughout the paper except otherwise stated. The presence or not of publication bias was investigated by funnel plots using the Review Manager software, Version 5.3.4. (Cochrane Collaboration Copenhagen, The Nordic Cochrane Centre, Copenhagen, Denmark) and Egger’s regression analysis using the Meta-Essentials tools (Rotterdam School of Management, Erasmus University, The Netherlands).

The descriptive data of the eligible studies is presented as means. Pooled estimates of the ES derived by either subgroup or comprehensive meta-analyses are presented as SMD and 95% confidence intervals (CIs) in square brackets (SMD [95% CI]). The alpha level for statistical significance was set at *p* ≤ 0.05 a priori.

## 3. Results

### 3.1. Study Characteristics

**Figure 2:**
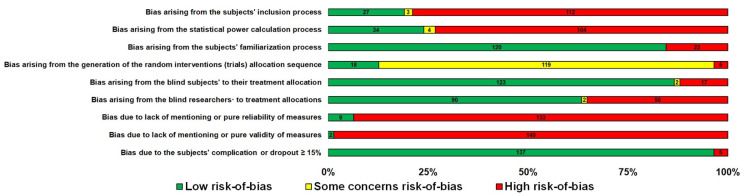
Overview of the authors’ judgments for each risk-of-bias item as a percentage in 142 trials.

An overview, along with the subjects’ characteristics, descriptive characteristics of the protocols used, and the effect (in SMD [95% CI]) of experimental CHO supplementation as compared to the control on an exercise task of the reviewed articles included in this meta-analysis, is reported in alphabetical order, by first author surname, in Table 1. The comparison of experimental CHO supplementation vs. control on exercise outcome, including raw data (in mean and SD [95% CI]) and risk-of-bias judgments of all trials is presented in a forest plot (Figure S1). The overview authors’ judgments for each risk-of-bias item are depicted as a percentage in Figure 2.

In total, 96 studies (142 trials) involving 1560 subjects in experimental groups (1534 in control groups) satisfied the inclusion criteria and were approved for further analysis. So, 142 SMDs were assessed and the total SMD [95% CI] estimate of 0.43 [0.35, 0.51] was significant, (*p* < 0.001). All studies used similar methods (pre-post design). The sample population of the control groups used was usually identical to that of the experimental groups in a crossover design, with the exception of three studies, which used a different sample population as control groups in a parallel design (Table 1). The participating subjects were mainly male, and their mean age was 19.3–44.0 (Table 1). Eighty-nine studies were lab-based and only seven field-based. The sport types of exercise used in the trials were cycling, running, triathlon, duathlon, roller-skiing, walking, loaded marching, swimming, and arm cranking (100, 35, 2, 1, 1, 1, 1, and 1, respectively; Table 1).

### 3.2. Effect of Carbohydrate Supplementation

**Figure 3:**
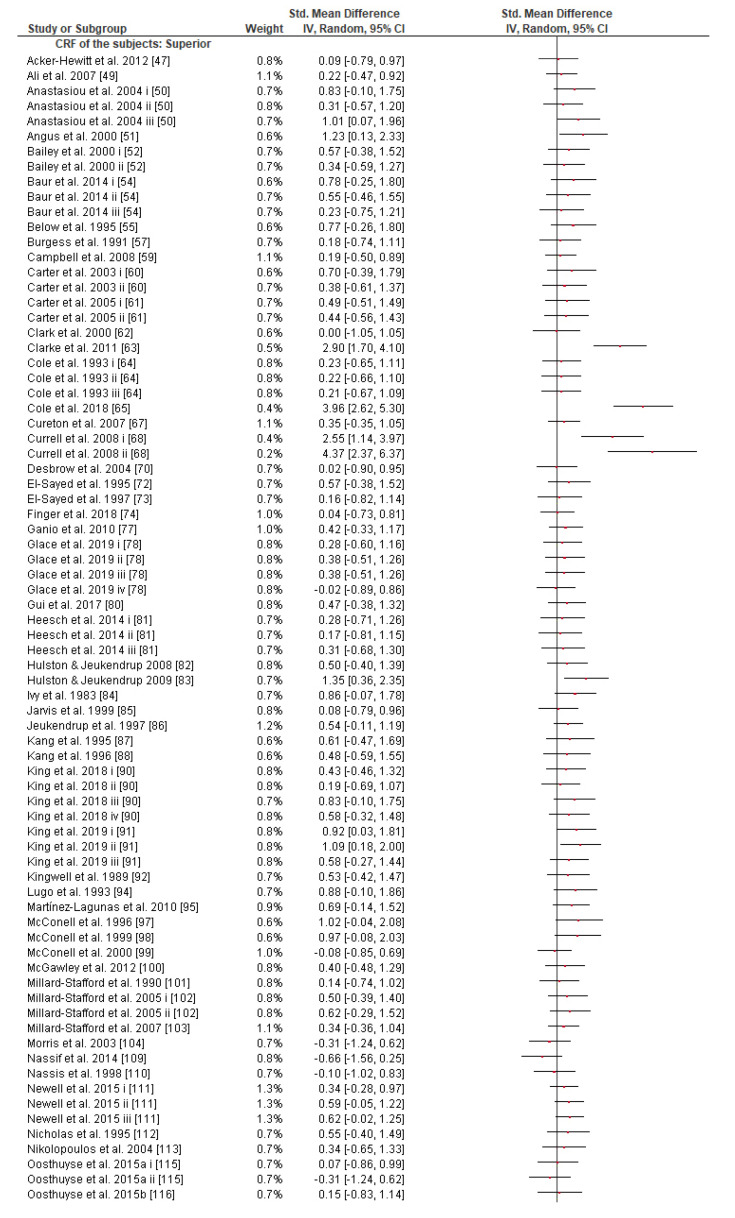

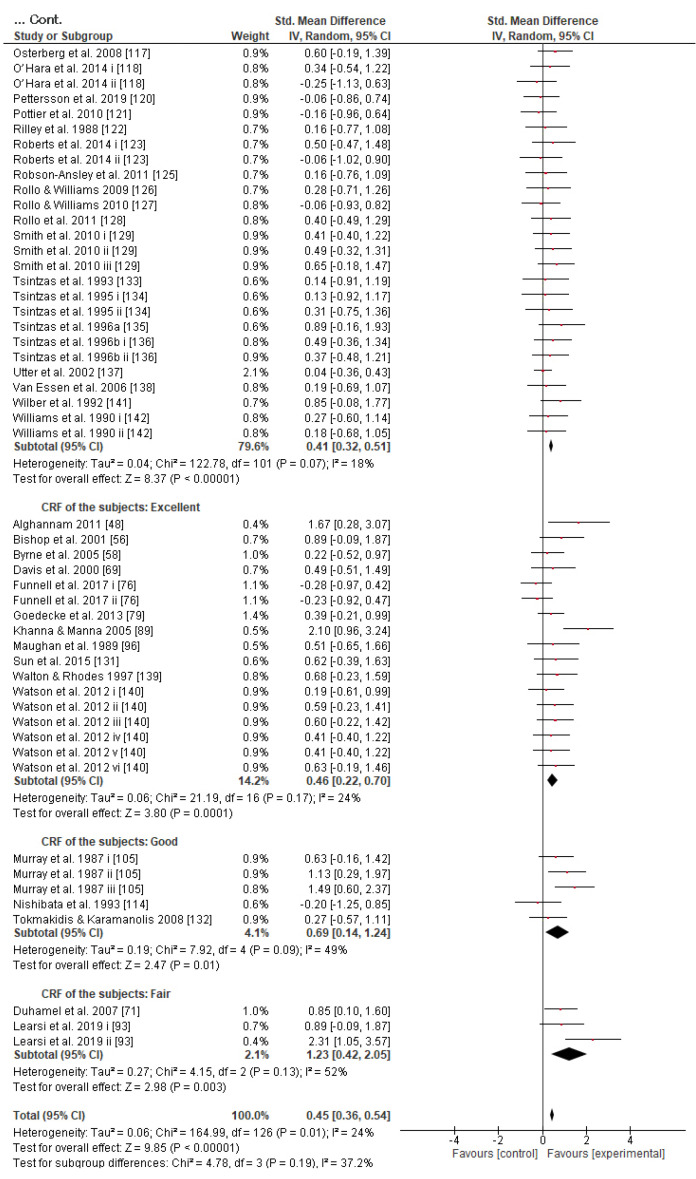
Forest plot shows the effects (red square symbol) of experimental carbohydrate supplementation as compared to a control on exercise outcome for 127 interventions [47,48,49,50,51,52,54,55,56,57,58,59,60,61,62,63,64,65,67,68,69,70,71,72,73,74,76,77,78,79,80,81,82,83,84,85,86,87,88,89,90,91,92,93,94,95,96,97,98,99,100,101,102,103,104,105,109,110,111,112,113,114,115,116,117,118,120,121,122,123,125,126,127,128,129,131,133,134,135,136,137,138,139,140,141,142]. Subgroup analyses show the results with regards to the exercise task in four exercise duration time groups (45 min ≤ T ≤ 60 min, 60 min < T ≤ 120 min, 120 min < T ≤ 240 min, T > 240 min). The black diamond symbol at the subgroups and at the bottom of the figure represents the standardized mean difference with the 95% confidence intervals for all interventions following random effects meta-analyses. Studies or trials that provided insufficient data for subgroup classification were not included. Abbreviations: CI, confidence interval; IV, inverse variance; SD, standard deviation; Std, standardized; T, time; i–vi denote different intervention arms (trials) within the same study.

Subgroup analysis showed that there were no significant differences between the age classes and subjects’ gender in the effect measurement (Figures S2 and S3). However, it is worth mentioning that although there is a tendency of the effect size to be reduced as the subjects’ CRF level increases according to the random-effects analysis (Figure 3), the fixed-effects analysis shows a significant effect size reduction as the subjects’ CRF level increases (*p* = 0.03, Figure S4).

**Figure 4:**
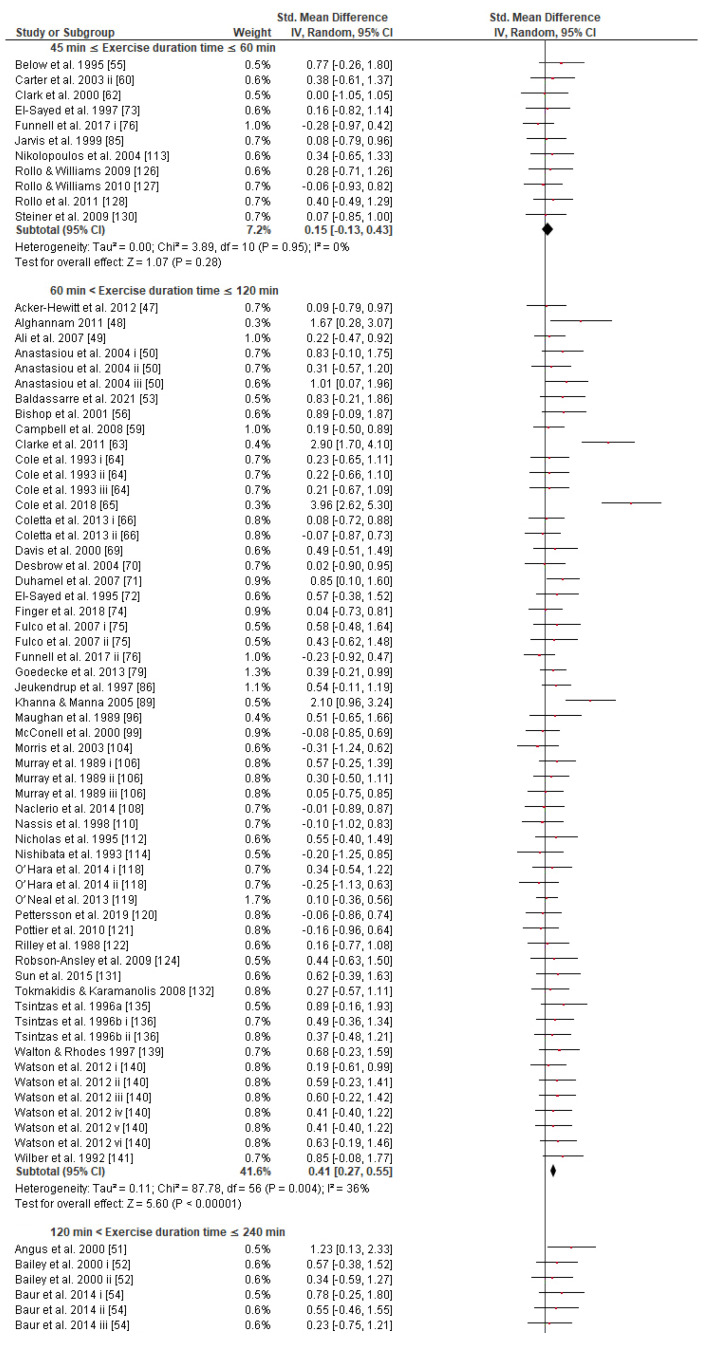

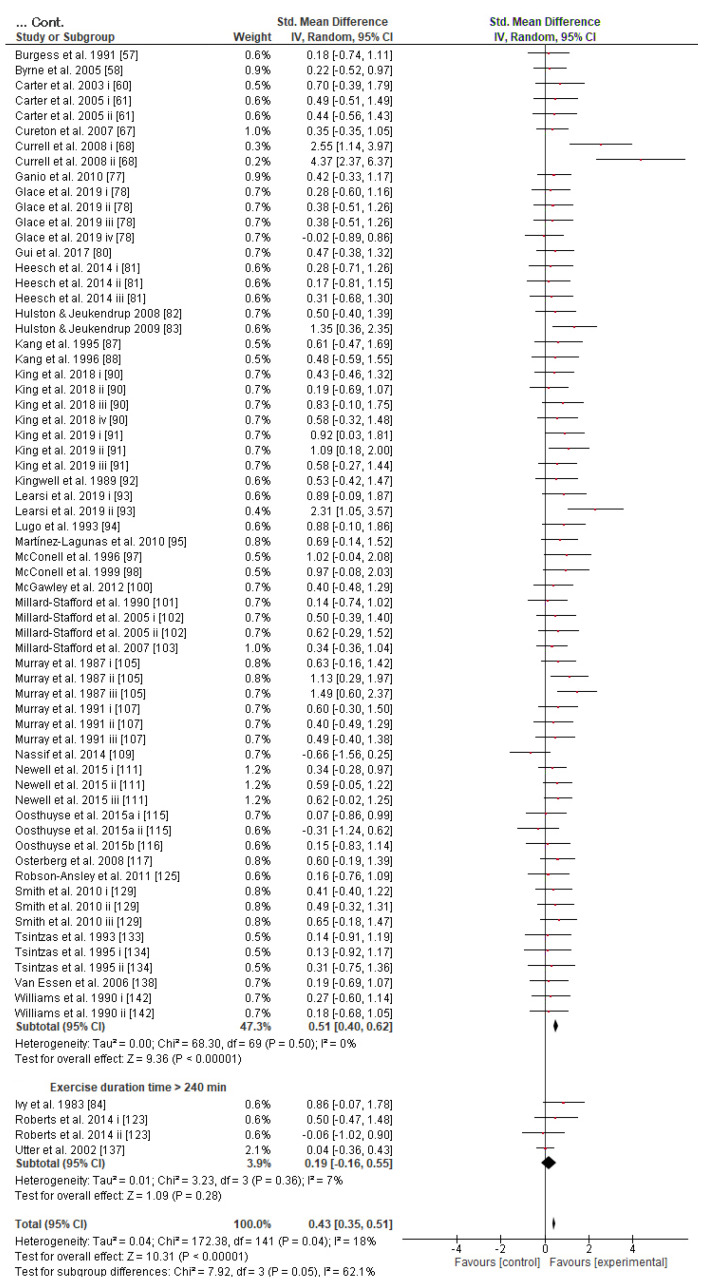
Forest plot shows the effects (red square symbol) of experimental carbohydrate supplementation as compared to a control on exercise outcome for 142 interventions [47,48,49,50,51,52,53,54,55,56,57,58,59,60,61,62,63,64,65,66,67,68,69,70,71,72,73,74,75,76,77,78,79,80,81,82,83,84,85,86,87,88,89,90,91,92,93,94,95,96,97,98,99,100,101,102,103,104,105,106,107,108,109,110,111,112,113,114,115,116,117,118,119,120,121,122,123,124,125,126,127,128,129,130,131,132,133,134,135,136,137,138,139,140,141,142]. Subgroup analyses show the results with regards to the exercise task in four exercise duration time groups (45 min ≤ T ≤ 60 min, 60 min T ≤ 120 min, 120 min T ≤ 240 min, T 240 min). The black diamond symbol at the subgroups and at the bottom of the figure represents the standardized mean difference with the 95% confidence intervals for all interventions following random effects meta-analyses. Studies or trials that provided insufficient data for subgroup classification were not included. Abbreviations: CI, confidence interval; IV, inverse variance; SD, standard deviation; Std, standardized; T, time; i–vi denote different intervention arms (trials) within the same study.

Regarding the exercise task, subgroup analysis showed that there were no significant differences between exercise modes, protocol tests, or type classes in the effect measurement (Figures S5–S7). What is remarkable, however, is the greater SMD in the cycling (0.47 [0.38, 0.57]) compared to running mode (0.35 [0.17, 0.52]) subgroup classes, though the effect is not significantly different. The effect of CHO interventions compared to control trials in the four exercise task duration time classes was significantly different between classes, (*p* ≤ 0.05, Figure 4). This revealed the advantageous role of CHO supplementation when CHO supplements are ingested during endurance exercise lasting 1–2 h (0.41 [0.27, 0.55]) or 2–4 h (0.51 [0.40, 0.62]) in comparison to exercise sessions lasting less than 1 h (0.15 [−0.13, 0.43]) or more than 4 h (0.19 [−0.16, 0.55]).

**Figure 5:**
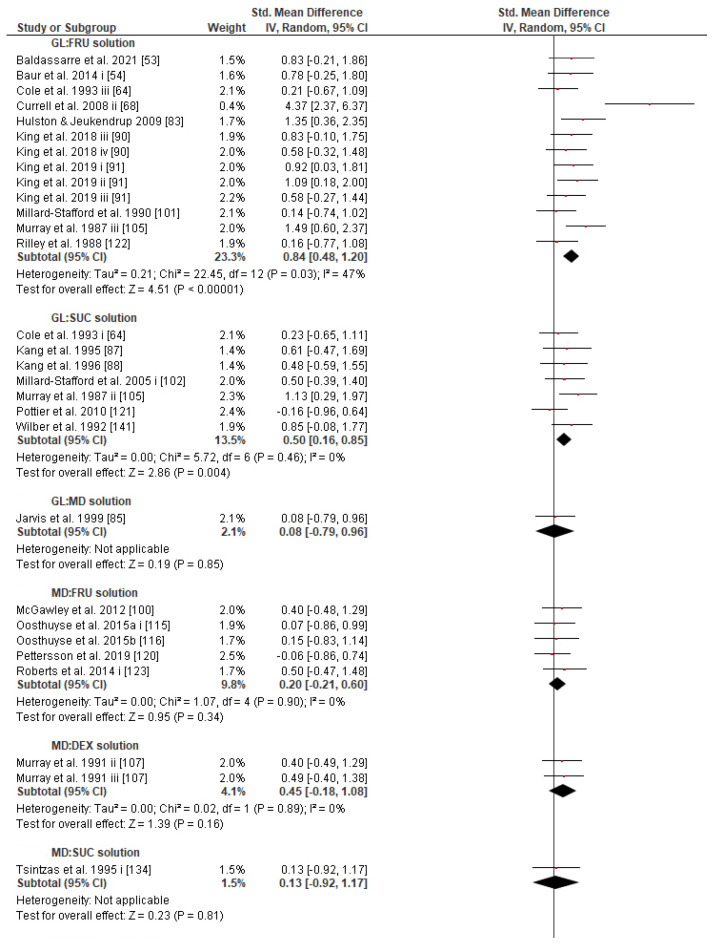

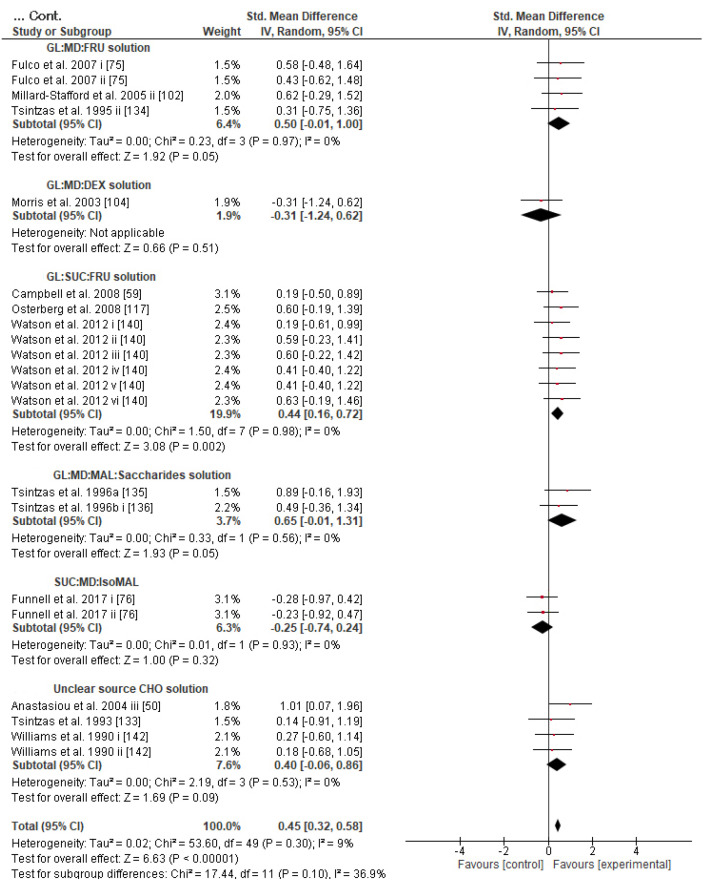
Forest plot shows the effects (red square symbol) of experimental carbohydrate supplementation as compared to a control on exercise outcome for 50 interventions [50,53,54,59,64,68,75,76,83,85,87,88,90,91,100,101,102,104,105,107,117,121,122,133,134,135,136,140,141,142]. Subgroup analyses show the results with regards to supplementation in 12 MTC groups (GL:FRU, GL:SUC, GL:MD, MD:FRU, MD:DEX, MD:SUC, GL:MD:FRU, GL:MD:DEX, GL:SUC:FRU, GL:MD:MAL:Saccharides, SUC:MD:IsoMAL, unclear CHO substances mixture). The black diamond symbol at the subgroups and at the bottom of the figure represents the standardized mean difference with the 95% confidence intervals for all interventions following random effects meta-analyses. Studies or trials that provided insufficient data for subgroup classification were not included. Abbreviations: CHO, carbohydrate, CI, confidence interval; DEX, dextrose; FRU, fructose; GAL, galactose; GL, glucose; IV, inverse variance; MD, maltodextrin; MAL, maltose; MTC, multiple transportable carbohydrate; SD, standard deviation; Std, standardized; SUC, sucrose; i–vi denote different intervention arms (trials) within the same study.

**Figure 6:**
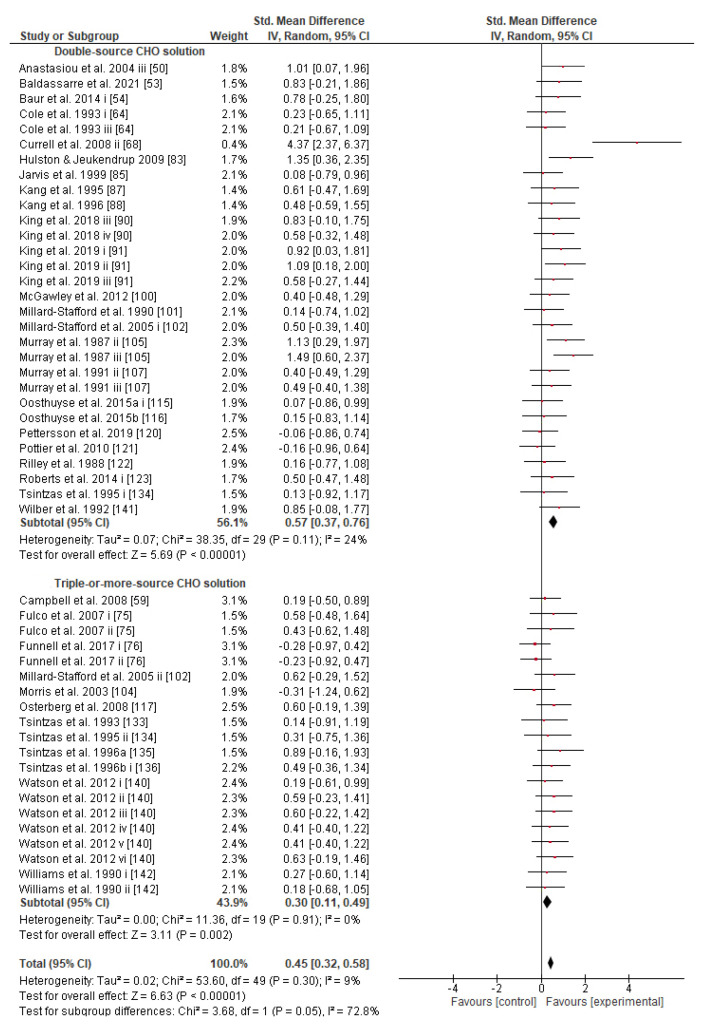
Forest plot shows the effects (red square symbol) of experimental carbohydrate supplementation as compared to a control on exercise outcome for 50 interventions [50,53,54,59,64,68,75,76,83,85,87,88,90,91,100,101,102,103,104,105,107,115,116,117,120,121,122,123,133,134,135,136,140,141,142]. Subgroup analyses show the results with regards to supplementation in two carbohydrate formulation groups (double-source CHO solution, triple-or-more-source CHO solution). The black diamond symbol at the subgroups and at the bottom of the figure represents the standardized mean difference with the 95% confidence intervals for all interventions following random effects meta-analyses. Studies or trials that provided insufficient data for subgroup classification were not included. Abbreviations: CHO, carbohydrate; CI, confidence interval; IV, inverse variance; SD, standard deviation; Std, standardized; i–vi denote different intervention arms (trials) within the same study.

**Figure 7:**
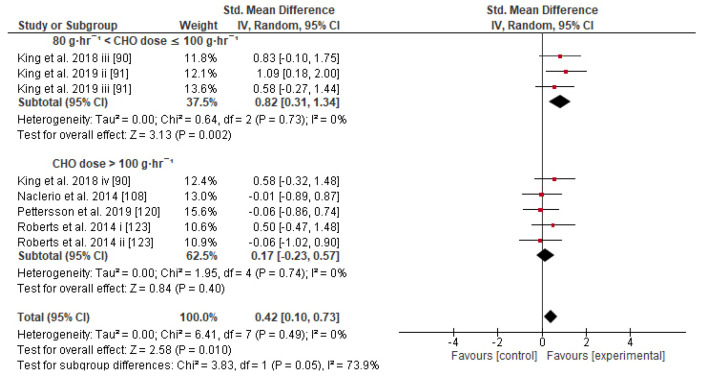
Forest plot shows the effects (red square symbol) of experimental carbohydrate supplementation as compared to a control on exercise outcome for 8 interventions [90,91,108,120,123]. Subgroup analyses show the results with regards to supplementation in two carbohydrate dose groups (80 g·h^−1^ < CHO dose ≤ 100 g·h^−1^, CHO dose > 100 g·h^−1^). The black diamond symbol at the subgroups and at the bottom of the figure represents the standardized mean difference with the 95% confidence intervals for all interventions following random effects meta-analyses. Studies or trials that provided insufficient data for subgroup classification were not included. Abbreviations: CHO, carbohydrate; CI, confidence interval; IV, inverse variance; SD, standard deviation; Std, standardized; i–vi denote different intervention arms (trials) within the same study.

**Figure 8:**
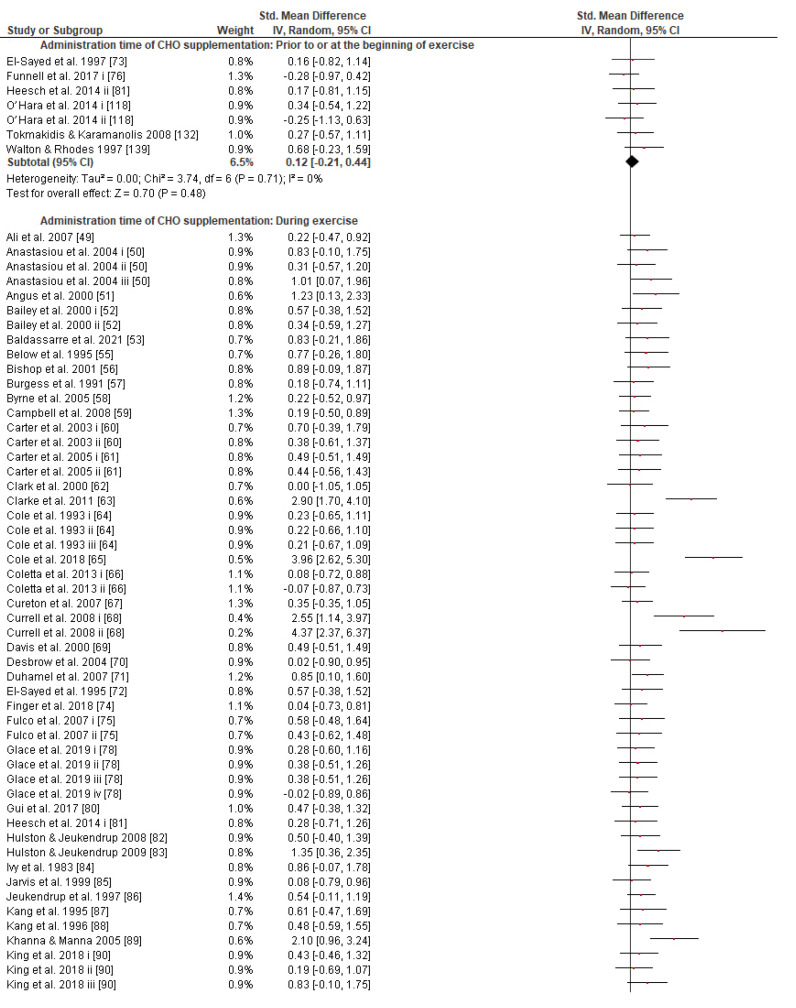

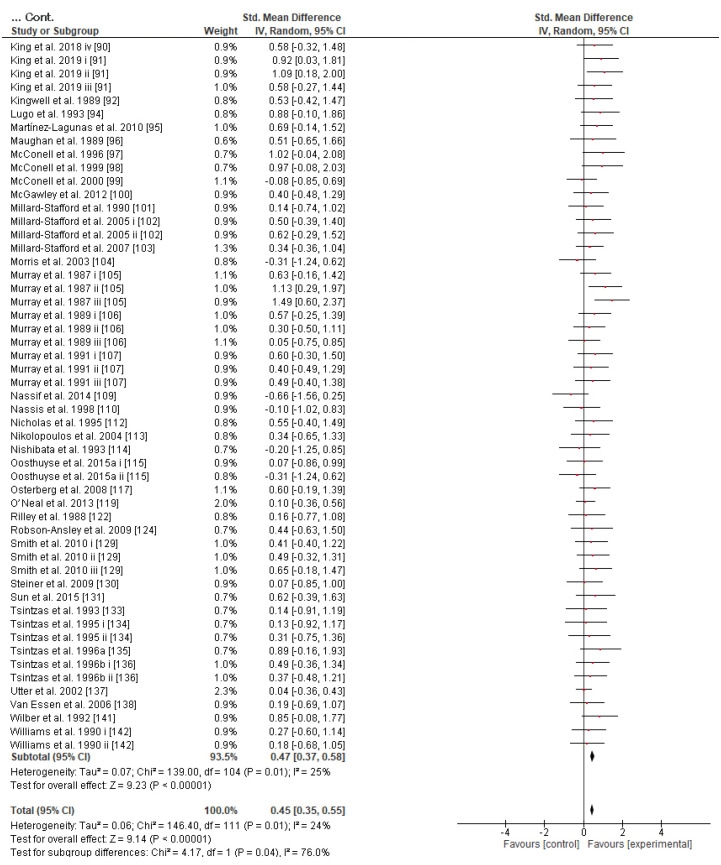
Forest plot shows the effects (red square symbol) of experimental carbohydrate supplementation as compared to a control on exercise outcome for 112 interventions [49,50,51,52,53,55,56,57,58,59,60,61,62,63,64,65,66,67,68,69,70,71,72,73,74,75,76,78,80,81,82,83,84,85,86,87,88,89,90,91,92,94,95,96,97,98,99,100,101,102,103,104,105,106,107,109,110,112,113,114,115,117,118,119,122,124,129,130,131,132,133,134,135,136,137,138,139,141,142]. Subgroup analyses show the results with regards to supplementation in two carbohydrate administration time groups (prior to or at the beginning of exercise, during exercise). The black diamond symbol at the subgroups and at the bottom of the figure represents the standardized mean difference with the 95% confidence intervals for all interventions following random effects meta-analyses. Studies or trials that provided insufficient data for subgroup classification were not included. Abbreviations: CHO, carbohydrate; CI, confidence interval; IV, inverse variance; SD, standard deviation; Std, standardized; i–vi denote different intervention arms (trials) within the same study.

Concerning the CHO supplementation, subgroup analysis showed that there were no significant differences in the SMD between different CHO supplementation concentrations, doses, types, composites, formulations, administration times, and temperatures of supplement administration classes (Figures S8–S13). It was, however, found by the random-effects analysis that CHO supplementation composed of GL:FRU has a slight tendency for a greater effect on performance in comparison to other MTC compositions (marginally insignificant, Figure 5). This tendency becomes a significantly greater effect of GL:FRU formation on performance in comparison to other MTC compositions using the fixed-effects analysis model, (*p* = 0.04, Figure S14). Further subgroup analysis showed that the effect measurement of the CHO supplement ingested was significantly higher when a double-source CHO solution formulation (0.57 [0.37, 0.76]) was used, in comparison to a triple-or-more-source CHO solution formulation (0.30 [0.11, 0.49]), (*p* ≤ 0.05; Figure 6). Regarding the administration of high CHO doses, the effect of CHO dose at rates > 100 g·h^−1^ had a significantly lower effect measurement (0.17 [−0.23, 0.57]) in comparison to dose rates of 81–100 g·h^−1^ (0.82 [0.31, 1.34]), (*p* ≤ 0.05, Figure 7). Moreover, CHO supplementation during exercise had a significantly higher effect (0.47 [0.37, 0.58]) in comparison to CHO supplementation when administered prior to or at the beginning of the exercise (0.12 [−0.21, 0.44]) (*p* ≤ 0.05, Figure 8). Lastly, not enough evidence was found to confirm or reject the hypothesis that the absence of differences in the effect measurement between different ambient thermal conditions after CHO supplementation was due to the limited studies available (Figure S15).

### 3.3. Risk of Bias

Evidence of some risk of bias was found in all included studies (Figure 2). The predominant risk of bias derived from the lack of reliability and validity of measurements in the studies (Figure 2). Risk of bias also derived from the subjects’ inclusion and statistical power process in the vast majority of the included studies (Figure 2). Furthermore, the Egger’s test of the intercept and inspection of the funnel plot suggested no potential publication bias (Figure S16).

## 4. Discussion

More than 45 years of research into the effects of CHO supplementation on performance during endurance exercise has provided a wealth of evidence to suggest that CHO contributes to fatigue postponement; yet, there have been no clear conclusions about the optimum administration timing, dosage, and type of CHO. Meta-analyzing the efficacy of CHO (≤20%) solution compared with control (placebo) on prolonged exercise performance in subjects over 18 years old found that regardless of the CHO supplementation time administration, form, and concentration, and exercise type, mode, or protocol, consumption of CHO solution has positive effects on performance. However, a crossover interaction between SMD and the subjects’ CRF level could exist. Carbohydrate solution seems to have a tendency to favor cyclists’ performance more than that of runners. As the exercise duration increases from 1 up to 4 h, the effect size of CHO intake on performance is also increased. A 6–8% CHO solution composed of two CHOs (GL:FRU) than more components appeared sufficient to increase the chances of a better performance. Interval frequency administration during the event proved superior to administration before the exercise. Moreover, though the effect size on performance seems to be unaffected by the use of different CHO doses schedules, it was found that a CHO dose of 80–100 g·h^−1^ has a significantly greater effect size on endurance performance in comparison to a CHO dose of more than 100 g·h^−1^.

### 4.1. Effects of CHO Supplementation on Endurance Exercise and Gastrointestinal Symptoms

In 2011, two meta-analyses reported a significant acute effect of CHO supplementation on endurance exercise performance (time trials or time to exhaustion tests) [143,144]. In the first one, the mean effect size of CHO solution intake on performance, derived from 50 studies, ranged from 0.30 to 0.53 [144]; in the second one, it derived from 73 studies (122 trials) and ranged from ~2 to ~6% [143]. Three years later, another meta-analysis demonstrated a positive influence on endurance performance in 82% of the 61 studies included (*n* = 679 subjects) and no effect in the remaining 18% [145]. Another systematic review, also comparing CHO solution(s) with placebo or water conditions, showed an improvement of exercise performance in 13 of the 17 studies included [146]. Similarly, we found strong evidence supporting that CHO solutions favor performance in prolonged exercise in 88.9% of the trials included, with an overall SMD = 0.43, (*n* = 1560).

On the other hand, high concentrated CHO sport solutions (>8% CHO) may in some cases impede the process of fluid absorption, hinder performance, [147,148,149,150], and additionally cause unpleasant GI distress [151,152]. It has been observed that GI symptoms are associated with endurance exercise after CHO feeding mainly in long-distance running and triathlon [153,154]. Mesenteric blood flow is reduced when exercise intensity is high, and notably when participants are hypohydrated. This is thought to be one of the main causes of the development of GI symptoms among other physiological, mechanical, psychological, or nutritional factors [155,156]. These GI symptoms might impede performance, making it difficult for athletes to win or even to follow the race [155,156]. In this review, we found three trials in which GI problems were reported during running only; one of them did not use FRU [134] and the other two used FRU in combination [134,135]. Fructose seems to be absorbed slowly from the intestinal tract and may be responsible for a significant osmotic effect in the intestines, which may cause GI problems [157]. This may have been the case in the above studies; however, there is insignificant evidence to further investigate this hypothesis in this study, as the causal mechanisms for most GI problems are still unclear. Until this is resolved, it may be wise for the athlete to avoid single FRU as a CHO supplement.

### 4.2. Effects of Mode, Protocol, and Type of Exercise

CHO supplementation supposedly has different effects on performance in different modes of exercise. This may be due to different muscle groups at work (for instance, during cycling, the upper body muscles do not contribute as much as in a triathlon or cross-country skiing) or to possible differences in the CHO absorption rate, which may be aided by small stomach movements during different exercise modes (for instance, running vs. cycling). Undoubtedly, the athletes’ weight is one of the determinant factors of energy expenditure, running economy, and, consequently, endurance exercise performance [158]. As running is less mechanically efficient than cycling, runners during given mechanical work exercise would be expected to have greater energy requirements than cyclists; therefore, a runner’s performance would be more CHO dependent [159]. This simply means that when both athletes ingest the same amount of CHO supplements, the cyclist may have a greater advantage in exercise performance compared to the runner, under similar work exercise conditions. It has been reported, however, that the ergogenic action of exogenous CHO during exercise, cycling or running, although depending on different physiological and metabolic mechanisms, is similar and of a comparably relative intensity; exogenous CHO oxidation rates between long running and cycling are identical as well [160,161]. Our findings show that CHO supplementation in cyclists tends to have a larger effect size on performance in comparison to runners (Figure S5). These results may be explained by what was mentioned above; however, this topic requires further investigation. The lack of an adequate number of comparative studies on other exercise modes prevents us from drawing conclusions.

Previous studies have demonstrated that fatigue during prolonged exercise in subjects fed CHO supplement occurs at approximately the same time as muscle glycogen depletion [162,163,164,165,166]. On the contrary, there seems to be no dose–response association between CHO uptake and improved exercise capacity [160]. However, our results indicate that the effect size of CHO supplementation on performance has no significant difference between capacity and performance tests (Figure S6). It must be noted, by the way, that performance tests better simulate real-world competitive endurance sports and are more reliable [167]. Therefore, the effects of CHO supplementation on performance between time trial and time to exhaustion tests seem identical and are in agreement with other relevant systematic reviews [144,145].

Originally, most of the studies investigated the role of CHO supplements during continuous (rather than intermittent) endurance exercise and showed an increase in endurance efficiency. During the prolonged intermittent and intermittent shuttle exercise (periods of intermittent bursts of high or lower intensity exercise (punctuated by rest or lower intensity activities)), energy is derived conjointly from anaerobic and aerobic metabolism [8,168]. So, in conditions of prolonged multi-sprint activities, the intra-muscular phosphocreatine and muscle glycogen stores are gradually depleted, and deterioration of performance is unavoidable [8,168,169]. Consequently, the research community has relatively recently extended its interest to investigating CHO supplements on performance during various intermittent exercise disciplines, especially in popular team games [8,168,169]. Nevertheless, the multi-complex nature of ‘stop and start’ sports (which makes it hard not only to simulate them but also to measure the subjects’ physical performance (i.e., for non-technical skills) in a reliable and accurate manner) and the various methodological approaches applied among studies create conditions that complicate an interpretation of the results. The CHO concentration of sport solution that was used so far in these studies was ~6–8% and the quantity of CHO (GL, SUC, and/or MD) ingested was ~30–60 g·h^−1^ [169]. Based on the relevant studies, for ‘stop and start’ sports (lasting from 1 to 2.5 h) that have shown an improved performance (or no effect), consumption of CHO at rates of 30–60 g·h^−1^ as an acute fueling strategy is generally recommended [8,9,169]. Similarly, we originally found strong evidence that CHO intake solutions have a significant and similar impact on performance during prolonged intermittent, intermittent shuttle, and continuous endurance exercise (Figure S7). However, the positive effect of CHO intake solutions on intermittent shuttle exercise (60 min < T ≤ 120 min) in the included studies derived from trials that had used mainly single-source CHO solutions (MD or SUC at a concentration range of 6.4–7%, [48,49,63,79]), with only one of them having used a CHO dose intervention (SUC 32.6 g·h^−1^ using a 7% solution, [79]). It is also worth mentioning here that in the three trials that had used >8% concentrated solutions in a range of CHO doses ~40–120 g·h^−1^, no positive result was found [76,108].

### 4.3. Effects of CHO Supplementation and Exercise Duration

Theoretically, CHO availability via the blood is not limited during exercise in individuals taking CHO supplements. Our findings show that: (a) as exercise duration increases up to 4 h, so does the effect size of CHO intake on performance (Figure 4); and (b) the positive SMD remains significant in exercise lasting more than 4 h; however, the effect is tempered. As muscle glycogen is gradually reduced during a 1–4-h all-out endurance exercise, the positive impact of CHO intake on exercise performance increases. As the duration of the exercise is prolonged (>4 h) and the intensity inevitably decreases, the performance should depend less on the availability of CHOs [19], since the percentage of energy contribution of CHO will be tempered in comparison to fats [170,171,172,173]. Thus, it is not surprising that most athletes (subjects) consume less CHO (~20–40 g·h^−1^) than the guidelines suggest [9] in endurance races lasting from 4 to 24 h [174,175,176,177,178]. The positive effect of CHO intake on performance in exercise lasting 45 min to 1 h appears to also be tempered in the included studies (Figure 4) (although a 6.4% CHO solution administration showed better results, [60,113,126,127,128]). Possible explanations are that (a) muscle glycogen is not fully depleted in this duration [18], (b) muscle fatigue is possibly due to the accumulation of H⁺ [19], and (c) the ergogenic effect of CHO solution may not be exclusively metabolic but could also be attributed to the CNS [7]. Similar evidence was also reported in one meta-analysis and one systematic review [144,145].

### 4.4. Effects of CRF and Gender on CHO Supplementation

Given that (a) GL expenditure is closely correlated with exercise intensity [179], (b) training reduces the flux of GL (so athletes may rely more on fat catabolism) for a specific power output [179], (c) endurance preparation reduces endogenous oxidation of blood glucose during prolonged exercise [180], (d) trained athletes could resynthesize better GL from lactate in comparison to untrained ones [11,181], and (e) muscle glycogen has a direct impact on CHO availability and CRF [182], it could be assumed that the more the athletes trained, the less they depend on exogenous CHO during prolonged exercise [11,179,183]. Indeed, we observed an interaction between SMD and the subjects’ CRF level (Figure 3 and Figure S4). Two previous reviews had partially discussed the potentially limited effect of exogenous CHO on the performance of trained endurance athletes as opposed to untrained ones [160,182] without reaching a permanent conclusion. The present study is probably the first one to report clear evidence as the fixed effect analysis indicated; nonetheless, more research is needed on this topic, since the conservative random effect analysis failed to prove any CRF effect on the efficacy of CHO intake during exercise (Figure 3).

In terms of sex, the already existing guidelines often assumed a similarly acute exercise effect of CHO supplementation in males and females. A study with a mixed-gender group [184] revealed that males and females responded in a similar fashion to CHO supplementation enriched with protein. On the contrary, it has been well documented that females oxidize more fat during endurance exercise than males and seem to metabolize endogenous CHO in different degrees, a process influenced by estrogen levels [12,52,185,186,187,188]. Our findings indicate no different effects of CHO supplementation on endurance performance between male (111 trials) and female subjects (8 trials) (Figure S2). However, it should be noted that in two of the trials, the menstrual status of the female participants was not controlled [78,139]. The well-established sexual dimorphism in muscle mass, hemoglobin concentration, level of reproductive hormones, and CHO oxidation [185,189,190,191], as well as the limited number of trials with female-only populations do not allow us to draw safe comparative conclusions.

### 4.5. Effect of Different CHO Supplement Concentrations and CHO Solution Formulations

The energy-dependent sodium-glucose link transporter (SGLT1) actively transports GL (which may also derive from other hydrolyzed CHO sources, such as starch, MAL, MD, or GL-polymer) and GAL through the intestinal mucosa [7,192,193,194]. Other CHOs, such as FRU and SUC, use GLUT5 (facilitated diffusion) and SCRT (if SUC is not hydrolyzed into GL and FRU) protein transporters, respectively [7,192,193,194]. Between GL-only and FRU-only (i.e., single-source CHO solution formulation), FRU has been theorized to be a better source of CHO than GL because it is absorbed more slowly than GL and therefore does not stimulate an insulin response [7,195,196]. Accordingly, the oxidation rate of ingested FRU during endurance exercise is slower than GL [197,198,199] or similar [200]. With the exception of FRU, GAL, IsoMAL, and starches (oxidation rate up to 40 g·h^−1^) [201], exogenous CHO is oxidized at a maximum rate of approximately 1 g·min^−1^ (60 g·h^−1^) [7,202,203,204,205]. Glucose-polymer has also been shown to be more digestible, with faster gastric emptying than GL, and could hence provide GL and fluids more effectively [206,207,208,209,210]. Moreover, a combination of different types of CHO like GL:FRU in a ratio of 2:1 (referred to as MTC, because they use multiple protein transporters (SGLT1 and GLUT5) [211]) speeds up oxidation rates up to 25% more than was previously thought (due to the GL barrier of 60 g·h^−1^). A series of studies also demonstrated even higher oxidation rates, up to 75%, using MTC (GL:FRU in ratio of 2:1) [201,212]. Moreover, it seems that ingestion of a solution composed of MTC during prolonged exercise (≥2–2.5 h) at high rates (>60 g·h^−1^) benefits performance more than the consumption of sport solutions containing GL-only (or MD-only) and minimizes the chances of GI discomfort [7,68,213,214,215,216].

The main goal of the intake of CHO sport solution during exercise is euhydration and euglycemia maintenance, and the minimum GL concentration required to boost water absorption is 0.9% [217]. So, in order to sustain an adequate glycogen supply and prevent hypoglycemia in exercise events lasting >1 h, a 6–8% CHO solution was generally recommended in many reviews [12,146,218,219]. Briefly, most modern reviews give more appreciation to solutions of 6–8% CHO concentration than to less or more concentrated drinks, and more credit to solutions composed of MTC than to single-source CHO solutions (e.g., GL or MD-only), in order to achieve high oxidation rates (>90 g·h^−1^), and consequently ensure better performance during prolonged exercise [7,9,12,143,145,146,216,218,219,220,221]. Similarly, the present meta-analysis provides evidence that CHO supplementation composed of GL:FRU is superior to other MTC compositions (fixed effect analysis; Figure S14). According to our results, the 6–8% CHO concentrated solutions show the highest SMD (Figure S8), and the double-source CHO solutions increase performance significantly more than triple-or-more-source CHO solutions (Figure 6). These findings combined show that a 6–8% CHO concentrated solution composed of two types of CHO (GL:FRU in a ratio of 2:1) is sufficient to increase the chances of a better performance. This statement has to be considered with caution since the effect of GL:FR on performance, in the present study, becomes weak as far as the random effect analysis is performed (Figure 5). It appears that further investigation is needed. With regard to interventions of various single-source CHO-only solutions (53 trials), although no significant differences in efficacy were found (Figure S10), the vast majority of trials (49) appear to have used MD-only, GL-only, or SUC-only solutions, which also showed greater SMD values in comparison to MAL-only, FRU-only, or GAL-only solutions (4 trials) [145]. This is obviously the reason why in some reviews, GL, MD, and SUC are recommended in cases where a single-source CHO solution is preferred [145,169].

### 4.6. Effect of CHO Administration Time and Dose

Regarding CHO administration time (i.e., prior to vs. during vs. late in exercise), there seems to be a lack of relevant comparative studies and only a few reviews have addressed this topic, albeit not extensively [12,160]. Briefly, it is reported that interval CHO administration during exercise is prioritized over feeding prior to exercise on its own [12]. Given that we did not study the CHO administration time pattern (e.g., feeding every 10 min vs. 20 min vs. 30 min vs. 40 min), our results reveal the advantage of interval CHO ingestion during exercise for performance as opposed to administration prior to exercise on its own (Figure 8). However, a prior-to-exercise CHO feeding protocol also has a significant effect on performance that cannot be neglected, and it could be used as an added strategy or on its own as long as it does not cause gastrointestinal discomfort or provoke insulin disturbance and therefore possible rebound hypoglycemia (Figure S12).

Concerning the CHO dose–response, our results show that the effect size on performance is unaffected by the use of different CHO dose schedules (g·h^−1^), probably because of the large variance of SMD among different trials that used different experimental methods (Figure S9). It also appears that performance is benefited significantly more from a CHO dose of 80–100 g·h^−1^ in comparison to a CHO dose >100 g·h^−1^ (Figure 7), and also more in comparison to 60–80 g·h^−1^, though not significantly (*p* = 0.15). On the other hand, some reviews consider that CHO intake of up to 60 g·h^−1^ for exercise lasting up to 2.5 h and up to 90 g·h^−1^ when the exercise duration exceeds 2.5 h should be recommended [7,9,220]. For the most part, these recommendations seem based on a previous review [221], which in turn seems to have premised its arguments on four previous studies about trained male endurance cyclists only [68,129,215,222]. The first study (*N* = 8) did not actually test the dose–response in terms of CHO g·h^−1^ intake but tested the CHO ingestion at a rate of 1.8 g·min^−1^ during 120 min of exercise where the significant effect was confirmed [68]. The second study (*N* = 9) compared the effect of two different iso-caloric beverages, containing GL or GL:FRU (no placebo/control group), at an ingestion rate of 2.4 g·min^−1^ ~144 g·h^−1^ on exercise lasting more than 3 h [215]. The third study (*N* = 12) compared ingestion of a placebo or CHO at doses of 15, 30, and 60 g·h^−1^ (but not a higher dose, such as 80 or 90 g·h^−1^) on exercise lasting more than 2 h [129]. The last study (in an abstract form at that time, *N* = 51) suggested an CHO ingestion dose between 60 and 80 g·h^−1^ for an optimum performance enhancement when exercise lasts 2 h [222]. Based on this evidence, it is more reasonable to suggest a CHO ingestion dose of 60–80 g·h^−1^ for exercise lasting up to 2.5 h in the trained male cyclists population only, rather than recommend a CHO intake up to 60 g·h^−1^ for exercise lasting up to 2.5 h and up to 90 g·h^−1^ when the duration of exercise exceeds 2.5 h for the general population in various exercise modes.

In 2013, Jekendrup (one of the leading researchers in the field) argued that, with a few exceptions, decent dose–response research was notably absent and many of the published reports had substantial methodological flaws, which made it difficult to establish a valid relationship between the quantity of CHO consumption and the efficiency of the dose–response [223]. In the absolute sense of the dose–response term (i.e., g·h^−1^), relevant research studies are still limited at the present time [169]. We only found a total of 11 relevant studies that satisfied our inclusion criteria (Figure S9). Regardless of this, most modern reviews recommend a CHO intake up to 60 g·h^−1^ during an endurance event lasting up to 2.5 h, and up to 90 g·h^−1^ when the duration of exercise exceeds 2.5 h [7,9,143,220]. Nevertheless, as the duration of an all-out endurance exercise decreases and the intensity increases accordingly, performance should depend more on the availability of CHOs, since CHOs will contribute to a higher energy percentage than fats [19,170,171,172,173]. Therefore, much more energy would need to be derived from CHO sources as a percentage in an exercise lasting up to 2.5 h than when the exercise exceeds 2.5 h [170]. A CHO ingestion dose of ~80 g·h^−1^ has also been suggested for an optimum performance benefit when exercise lasts ~2 h [224]. So, in endurance events lasting up to 2.5 h, if we supply 90 g·h^−1^ of MTC (e.g., GL: 60 g·h^−1^ and FRU: 30 g·h^−1^, taking advantage of both different CHO transporters, SGLT1 and GLUT5, respectively), theoretically, the chance of an optimum CHO oxidation rate [225] will be increased [226], thus increasing the chances of a higher performance in comparison to 60 g·h^−1^ GL-only (or MD-only) ingestion [227]. In this case, if the extra CHOs are not fully oxidized in the first 2.5 h of the endurance task, the residual CHOs will probably help reduce the overall amount of CHOs needed for replenishment in the later stages of the exercise or post exercise and may favor the athlete for upcoming exercise events series. Consequently, with our findings in mind, since GI comfort is not compromised [216,228], the recommended CHO dose could also be raised up to 90 g·h^−1^ (instead of 60 g·h^−1^) composed of MTC for endurance events lasting 1–2.5 h [10,90,145,146].

The main reason for recommending a CHO dose up to 90 g·h^−1^ (instead of 60 g·h^−1^) when exercise duration exceeds 2.5 h is to compensate for the metabolic demands due to the increased glycogen depletion rates [7,9,143,220]. However, this guideline is very general and may not consider all the multifactorial metabolic demands of various ultra-exercise events, as (a) most guidelines do not take into account body mass (BM) and sex differences. (b) Controversially, the above recommendation equates in theory the energy requirements in exogenous CHOs for endurance races that just last >2.5 to those for races that last, for instance, 4, 8, 12, and 24 h, which obviously have different intensities and metabolic demands. As the duration of an ultra-endurance event increases and the intensity decreases accordingly, the performance should depend less on the availability of exogenous CHOs [19,170,171,172,173]. We found that the effect size of CHO intake on performance is smaller for an exercise duration >4 h (4 trials) (Figure 3). It was also revealed that (a) runners’ (subjects’) consumption was surprisingly 14.93 g·h^−1^ in race durations ~4.28 h [175]; (b) most athletes’ consumption rate ranged from 0.27–0.64 g·kg^−1^·h^−1^ in race durations ~24 h [174] or 20–40 g·h^−1^ in single-stage and multistage ultramarathon events [176,177,178]; and (c) mean consumption was 62.2 g·h^−1^ in race durations of 24 h [229], which is less than the prevailing recommendation. Therefore, as new evidence keeps coming up in the literature, we believe that the issue of exercise duration deserves further investigation, perhaps even a reconsideration of the hitherto proposed recommendations.

However, another question arises regarding dose: Why are the doses recommended so far expressed mainly in g·h^−1^ units and not, correspondingly, in individual BM, as proposed [9], given that heavier athletes consume more energy than lighter ones? On the one hand, a meta-analysis has reported that the ergogenic action of a CHO dose up to 80 g·h^−1^ depends on the athlete’s size among other factors [144]. On the other hand, it has been concluded that that there is no evidence for expressing CHO dose guidelines in relation to BM because they are not correlated [201], a conclusion that was later adopted by several other reviewers [7,9,143,220]. This viewpoint appears to be based on the results of a series of CHO oxidation rate studies [161,230,231,232,233]. However, research in these studies was conducted in the same laboratory, on trained male cyclist subjects only (BM ranged from ~58–84 kg), whereas exercise performance was not tested, which limited the results’ generalizability. Moreover, the subjects in these studies followed their own diet (except the last 1–2 days) before the experiment [161,230,231,232,233]. Since the CHO content of any diet is a determinant factor of the gut absorption capacity, as later studies have shown (due to the intestinal transporters’ upregulation when the amount of CHO intake is increased [201,234,235]), it should have a significant impact on the oxidation rate. In this sense, as the subjects did not experience the same diet routine, it is reasonable to assume that their gut was trained differently, and they would have different CHO absorption rates [234,235], most probably uncorrelated to their BM, and thus different oxidation rates. This alone should call for a re-examination of the notion of expressing CHO dose guidelines in g·h^−1^ values.

### 4.7. Methodological Aspects, Strength, Limitations, and Suggestions for Future Research

Though meta-analysis has been widely applied to human performance research [236,237,238,239], its results have been criticized as ‘mixing apples and oranges’ [240]. However, what needs to be considered is whether studies of different internal validity and methodological control (e.g., small sample size, no control group, and non-randomized) should be included, and whether they should be considered as weighty as studies with more appropriate experimental designs. For this reason, we only included controlled intervention studies, whose authors reported that they used a specific experimental method involving a comparison group in a parallel or crossover design. We also introduced a modified version of the Cochrane risk-of-bias assessment tool to assess potential limitations of the eligible studies [24]. Nevertheless, it should be emphasized that a comparison of the more methodologically sound studies is not sufficient on its own. The different methodologies and techniques employed by the different studies could also explain the lack of consistency in the literature. Furthermore, we attempted to adjust the methodological research characteristics employed throughout this review using specific predefined criteria; nonetheless, evidence of overall risk-of-bias in the included studies remains subjective to some extent [24]. In the current meta-analysis, the vast majority of the eligible studies used a crossover design (139 trials). We assume that the ~one week washout period reported by the revised studies was sufficient for subjects to recover from residual fatigue derived from the previous exercise testing session, and that the time of washout between repeated trials did not impact the key meta-analysis findings. Hence, crossover designs are not considered a threat to this study. The effect size has also been criticized in the meta-analysis technique. Hedges (1981), who introduced the unbiased effect size, suggested a solution to this, which was adapted for use in our review [36]. In an effort to further increase the homogeneity of our results and be able to draw conclusions on variant modes, durations, and types of exercise, and different classes of subjects CRF, compositions, concentrations, and administration times of CHO supplements, we also applied subgroup analysis. Although only studies with accessible full-text papers written in English were selected, our outcomes show no potential publication bias. It should also be stated that even though all methodological precautions were taken, the extended period (1975–2021) of studies being analyzed introduces a level of uncertainty in drawing conclusions, due to changes in the experimental methods and designs through time. Similarly, another point of introduced uncertainty in the present meta-analysis deals with the fact that performance was not measured in all studies in a uniform way. Time to exhaustion and time trail tests evaluate different physiological mechanisms and each one entails its own repeatability accuracy [32,33,34]. Despite this, the present study found no effect of the performance test on CHO efficacy as others also did [144,145].

Gut trainability derived from ‘nutritional training’ in order to train the GI tract by improving the rate of emptying and fluid absorption and thus favor exercise performance in endurance events is a known practice among endurance athletes [234,235]. However, no study reported that subjects were checked in advance, as to whether they used to ingest CHO supplements or a high CHO-enriched diet for a long period before the study, which means that we do not know if the results of these studies could have been affected by subjects’ gut trainability effect. To overcome this methodological flaw in future studies, all subjects will need to follow a standardized control diet for many weeks prior to the investigation period.

Most of the review studies were conducted on cycling (100 trials), in laboratory environments (133 trials) and in a male population (111 trials), making it hard to generalize to other natural sporting environments (i.e., aquatic activities), elite athletes, and the female population. Perhaps more applied research is needed to investigate the effect of CHO supplements on endurance exercise of different types, durations, and modes at different phases of a female’s menstrual cycle [241] and in elite athletes. Further, investigation is required to assess whether CHO solution administration is ergogenic in ultra-exercise events of varied duration, and what is the optimal dose, type, and concentration of a CHO drink for enhancing performance. Ambient temperature, exercise mode, and intensity seem to differently influence the energy substrate’s oxidation and metabolism [13,14]. We did not find enough studies on different hypobaric or thermal conditions combined with different CHO solution temperatures to draw safe conclusions; therefore, more research is needed to address this issue. In the last decade, however, there has been an emerging interest in CHO dose (CHO g·h^−1^) response in endurance performance, and various studies recommend 30–60 g·h^−1^ to 90 or even a surprising 120 g·h^−1^ [7,9,242]. As was earlier explained, the CHO dose–response in exercise performance could possibly depend on the athlete’s weight [144]. Since no modern study has investigated individuals’ dose–response control for their BM or lean mass in conjunction with their diet, this is another future research topic that needs to be addressed. Furthermore, any recommended dose that will be derived from these studies should preferably be expressed in CHO g·h^−1^·kg^−1^ of BM, as suggested by the American College of Sports Medicine. This will enable global scope organizations to update their recommendations and make them more suitable and accurate for a wider range of athletes’ body sizes [9]. In addition, as more investigation on dose–response is required, more studies are needed to determine the potential action of different CHO doses on exercise performance, especially in different exercise modalities and intermittent events. The validity and reliability of the experimental protocols should be measured and reported by the researchers accordingly.

In this article, we selected and analyzed relevant papers from a specific period (1975–2021) and verified the hypothesis that CHO supplementation during prolonged exercise enhances performance or delay fatigue progression. Maintaining blood glucose concentrations and increasing CHO oxidation rates could be the main reasons behind such performance improvement. Fatigue is not solely located at the peripheral level, as explained by the appearance of a “metabolic endpoint”, in which muscle glycogen levels are exhausted and plasma GL levels are lowered (peripheral factor) [243,244]. There is substantial evidence that mechanisms located at the CNS are also involved in fatigue exhibition (central factor) [245,246] and it seems that the CHO ergogenic effect also has a central component independent of the metabolic one [247]. The drop in central activation during persistent muscular contraction could be caused by substrates’ depletion in the CNS and/or changes in the levels of particular neurotransmitters [244]. Exercise-induced hypoglycemia has been linked to a significant reduction in voluntary activation during persistent muscle contractions [248], as well as a drop in brain GL uptake and the overall cerebral metabolic rate [249]. On the contrary, when euglycemia is maintained, the drop in CNS activation recedes [250]. Moreover, a decrease in brain GL has also been linked to the homeostatic drive to sleep [251], suggesting that it may play a role in fatigue progression, and changes in extracellular GL concentrations have been shown to have a considerable impact on serotonin release and absorption during exercise and recovery, indicating that GL plays a key role in central neurotransmission control [252]. Thus, as exercise activity duration increases (e.g., >2 h) and glycogen depletion becomes apparent, CHO supplementation is also expected to continue to have a significant influence on central fatigue markers [253]. However, the mechanisms that cause performance declines can interact at any level of the brain–muscle circuit, and while the literature normally distinguishes between peripheral and central fatigue, both pathways may be intertwined and the complicated relationship between peripheral and cerebral components could influence fatigue during prolonged exercise [254]. Therefore, as the effects of CHO supplementation on the interactive mechanisms of central and peripheral fatigue during prolonged exercise are still unclear [255], more research is needed on this complex phenomenon impacted by both central and peripheral factors.

### 4.8. Practical Implications and Guidelines for the Athlete

In the last 45 years, the undiminished interest in research on CHO solutions (sport drinks) has promoted our insights and sport supplementation practices. Gastrointestinal absorption, splanchnic and muscle blood flow, muscle energy uptake, and substrate oxidation have all been established as possible barriers to the potential ergogenic effects of CHO solutions [256]. However, with an ever-growing body of reviews on the influence of ingesting CHO solutions during prolonged exercise on performance, and different recommendations from a different perspective, there is the question of what constitutes substantive advice [7,8,9,10,11]. Moreover, many athletes have reported that is not very clear if the dose should be up to 60 g·h^−1^ for the first 2.5 h and then be increased to 90 g·h^−1^, or the dose should be 90 g·h^−1^ from the beginning of an endurance event when the exercise lasts more than 2.5 h (personal communication, in recent ultra-endurance races). There are also many who are misled by retailers, possibly due to the difficulty of interpreting the scientific data, the existence of a plethora of different guidelines, and various misconceptions [8,257,258].

**Figure 9:**
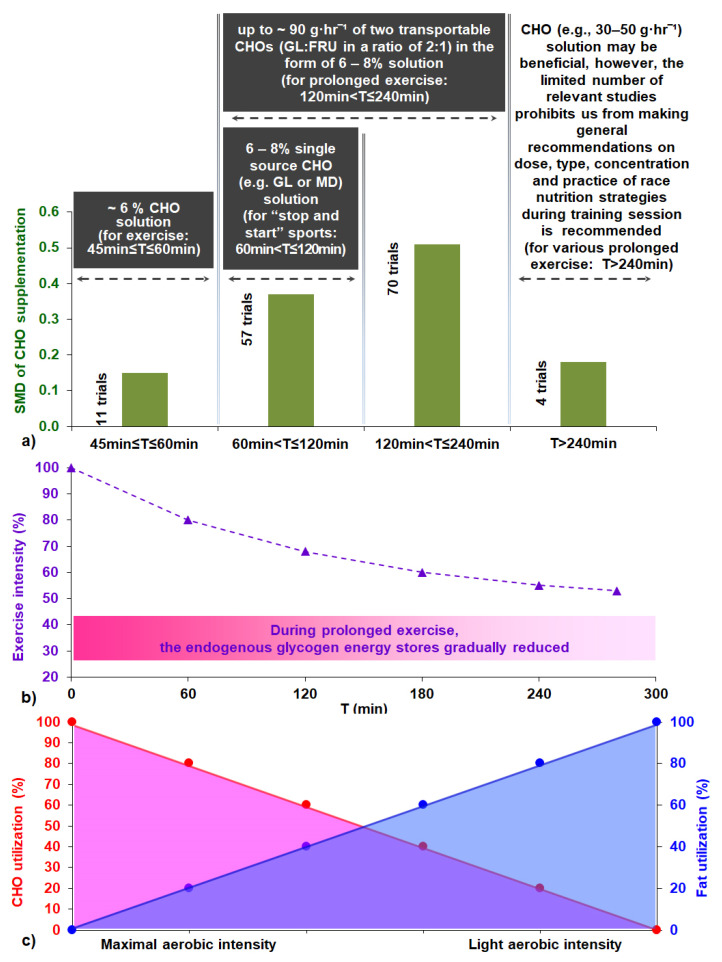
(**a**) Suggested key guidelines for carbohydrate supplementation during prolonged exercise and effect size of CHO supplementation as a function of exercise duration, referenced in the present study. (**b**) Idealized various intensities for constant exercise as a function of exercise time and endogenous glycogen energy stores reduction [170,171,172,173]. (**c**) Idealized fuels used as a function of exercise effort [170,171,172,173]. Abbreviations: CHO, carbohydrate; FRU, fructose; GL, glucose; MD, maltodextrin; SMD, standardized mean difference; T, exercise time.

Therefore, since our study does not cover the whole spectrum of research on CHO solution ingestion during prolonged exercise, our recommendations are inevitably limited by our findings. We also feel that it will be more appropriate to propose a more simplified version of the already existing guidelines to athletes [9], which is likely to have a greater impact on the sport population. Thus, we think that the same or an even better result could be obtained by simply recommending a dose up to 90 g·h^−1^ of two transportable CHOs (GL:FRU in a ratio of 2:1) in the form of a 6–8% CHO solution during exercise events (intermittent and continuous) lasting from 1 to 4 h (Figure 9). With regard to ‘stop and start’ sports, it seems that a 6–8% concentrated single-source CHO solution (e.g., GL or MD or SUC) is sufficient not only to serve hydration purposes, but also to meet increased demands for the exogenous CHO supply. For exercise lasting 45 min ≤ T ≤ 60 min, a ~6% CHO solution is recommended. Taking supplemental CHO solutions (e.g., 30–50 g·h^−1^ as proposed by the International Society of Sports Nutrition [177]) in prolonged exercise (T > 240 min) may be beneficial. Nevertheless, the fact that we found only three relevant studies prevents us from making universal recommendations; therefore, athletes should test and rehearse race nutrition strategies, including precise macronutrient/fluid, quality, and amount during training sessions. It should be duly pointed out that the guidelines suggested above are based on our analysis, which mainly derived from mild environmental condition studies, in the male population and non-elite athletes, and so their generalizability is limited to the study population/ambient condition. As perceived, scientific evidence should never be ignored. However, athletes’ CHO supplementation plans could be tailored to their individual preferences, responses, and tolerance to various dose strategies and needs. Athletes should also take into account the provided options for CHO solution ingestion in a competition event or training session [8,9,10,11,12,145,146]. Additionally, athletes should not neglect the benefit of a balanced diet, gut training, and pre-event CHO supplementation, and they should always be well hydrated [8,9,10,11,145,146,234].

## 5. Conclusions

We examined the efficacy of CHO (≤ 20%) solution compared to controls on prolonged exercise performance in subjects over 18 years old in 96 studies (142 trials). We found that CHO solution favors performance in prolonged exercise, continuous and intermittent, and cyclists tend to have a greater effect size in comparison to runners. As the exercise duration increases from 1 to up to 4 h, so does the effect size of CHO intake on performance. A crossover interaction between SMD and the subjects’ CRF level was also considered, although not between gender classes; however, the number of studies in the female population are limited and the athlete’s gender may be a worthy variable for future consideration. A 6–8% CHO concentrated solution composed of two transportable CHOs (i.e., GL:FRU in a ratio of 2:1) appears to be sufficient to increase the chance of a better performance. An administration schedule during the event seems superior for performance in comparison to administration prior to exercise on its own, although CHO supplementation prior to exercise should not neglected. Additionally, although the effect size on performance seems to be unaffected by different CHO dose schedules, it appears that a CHO dose of 80–100 g·h^−1^ has a greater effect size on performance in comparison to 60–80 g·h^−1^ and is significantly more beneficial for endurance performance in comparison to a CHO dose >100 g·h^−1^. However, all included studies investigating the efficacy of CHO supplements during prolonged exercise have shown some evidence of risk-of-bias, an issue that should be addressed in future research. More research is needed to investigate the impact of CHO supplementation and CHO dose, preferably expressed per kg of BM as suggested [9], on prolonged exercise of different types, duration, modes, in the female population, at different phases of the menstrual cycle, and in elite athletes. We hope that the findings of and questions raised by this review will help set a direction for future applied research.

## Figures and Tables

**Table 1:** Overview of the reviewed articles and the effect of experimental carbohydrate supplementation as compared to a control on an exercise task in SMD with 95% CI. Data presented as mean or range or otherwise stated. Where not reported in the articles, sufficient averaged data per sex subgroups and averaged pooled values for mixed sample groups are referred to, respectively.

Study/Trial †	Study Design	*N*/ Gender	Age (year)	BMI (kg·m^−2^)	$\dot{V}$O_2_max (mL·kg^−1^·min^−1^)	Lab/ Field	Exercise Task (Brief Description)	CHO Supplementation (Brief Description)	SMD IV, Random [95% CI)
Acker-Hewitt et al., 2012 [47]	CS	10/M	28.0	23.0	66.0	Lab	20 min of SS cycling [60% PPO (Wmax)] + a simulated 20-km TT, TA: 21.5 °C	8% solution, 250 mL fluid (CHO, 20 g) administered at: immediately prior to exercise, following the 20-min SS, and 20 min into the TT	0.09 [−0.79, 0.97]
Alghannam 2011 [48]	CS	6/M	26.0	21.9	51.4 *	Lab	75 min of intermittent football-specific running (interspersed with a 15 min recovery) + run TF at 80% $\dot{V}$O_2_peak, TA: 20.6 °C	6.9% CHO solution [MD, 1 g·kg^−1^] administered at: 15 min prior to exercise and at 45 min	1.67 [0.28, 3.07]
Ali et al., 2007 [49]	CS	16/M	21.3	23.0	56.0	Lab	90 min intermittent high-intensity shuttle running (~66 sprints) protocol [LIST: 15 min block consists of 10–12 repeated cycles of walking, running (at a speed equivalent to 95% $\dot{V}$O_2_max), jogging (at a speed equivalent to 55% $\dot{V}$O_2_max), and sprinting]	6.4% CHO-E solution (Lucozade Sport, GlaxoSmithKline, Brentford), 5 mL·kg^−1^ before and 2 mL·kg^−1^ every 15 min of exercise	0.22 [−0.47, 0.92]
Anastasiou et al., 2004 i [50]	CS	10/M	25.1	23.0	56.6 *	Lab	cycling time to complete the target amount of work (J) = 0.75 × Wmax × 3600, (~60 min), TA: 27.9 °C	GL-E drink, (0.65 g·kg^−1^) 15 min prior to exercise ~600 mL and during at 15-min intervals ~200 mL of GL-E drink (0.2 g·kg^−1^)	0.83 [−0.10, 1.75]
Anastasiou et al., 2004 ii [50]	CS	10/M	25.1	23.0	56.6 *	Lab	cycling time to complete the target amount of work (J) = 0.75 × Wmax × 3600, (~60 min), TA: 27.9 °C	MAL-E drink, (0.65 g·kg^−1^) 15 min prior to exercise ~600 mL and during at 15-min intervals ~200 mL of MAL-E drink (0.2 g·kg^−1^)	0.31 [−0.57, 1.20]
Anastasiou et al., 2004 iii [50]	CS	10/M	25.1	23.0	56.6 *	Lab	cycling time to complete the target amount of work (J) = 0.75 × Wmax × 3600, (~60 min), TA: 27.9 °C	CHO-mix-E drink, (0.65 g·kg^−1^) 15 min prior to exercise ~600 mL and during at 15-min intervals ~200 mL of CHO-mix-E drink (0.2 g·kg^−1^)	1.01 [0.07, 1.96]
Angus et al., 2000 [51]	CS	8/M	22.0	23.0	65.4 *	Lab	cycling time to complete “as quickly as possible” 35 kJ·kg^−1^, TA: 20.0–22.0 °C	6% CHO solution (Gatorade, Quaker Oats Co.), 250 mL at 15-min intervals	1.23 [0.13, 2.33]
Bailey et al., 2000 i [52]	CS	9/F	27.0	21.5	49.6 *	Lab	TE cycling at 70% $\dot{V}$O_2_peak, during follicular phase of the menstrual cycle, TA: 22.7 °C	6% CHO solution (Gatorade, Quaker Oats Co.), CHO 0.6 g·kg^−1^·h^−1^, (5 mL·kg^−1^ every 30 min beginning at min 30 of exercise)	0.57 [−0.38, 1.52]
Bailey et al., 2000 ii [52]	CS	9/F	27.0	21.5	49.6 *	Lab	TE cycling at 70% $\dot{V}$O_2_peak, during luteal phase of the menstrual cycle, TA: 22.7 °C	6% CHO solution (Gatorade, Quaker Oats Co.), CHO 0.6 g·kg^−1^·h^−1^, (5 mL·kg^−1^ every 30 min beginning at min 30 of exercise)	0.34 [−0.59, 1.27]
Baldassarre et al., 2021 [53]	CS	8/5 M 3 F	23.0	23.0	#	Lab	3 × 30 min (20 s interval) swimming at a pre-set pace (corresponding to 10-km) + a TE at 100% $\dot{V}$O_2_peak, TA: 27 °C	8% CHO solution, 45 g GL:FRU in ratio of 1:1 in 550 mL of water (Enervitene Sport Cheerpack, Enervit©) during each of the two intervals (total 60 g·h^−1^ of CHO)	0.83 [−0.21, 1.86]
Baur et al., 2014 i [54]	CS	8/M	25.0	23.8	62.0	Lab	120 min of constant-load cycling at 55% Wmax + a simulated 30 km TT	12% CHO-E beverage (GL:FRU in a ratio of 2:1: Tate and Lyle, Decatur, IL), 600 mL prior to exercise, 150 mL bolus every 15 min during the constant-load part of the trial (total: 1200 mL) and at three points during the 30-km TT (7.5, 15, and 22.5 km; total: 450 mL)	0.78 [−0.25, 1.80]
Baur et al., 2014 ii [54]	CS	8/M	25.0	23.8	62.0	Lab	120 min of constant-load cycling at 55% Wmax + a simulated 30 km TT	8% CHO-E beverage (moderate-GL beverage), 600 mL prior to exercise, 150 mL bolus every 15 min during the constant-load part of the trial (total: 1200 mL) and at three points during the 30-km TT (7.5, 15, and 22.5 km; total: 450 mL)	0.55 [−0.46, 1.55]
Baur et al., 2014 iii [54]	CS	8/M	25.0	23.8	62.0	Lab	120 min of constant-load cycling at 55% Wmax + a simulated 30 km TT	12% CHO-E beverage (high-GL beverage), 600 mL prior to exercise, 150 mL bolus every 15 min during the constant-load part of the trial (total: 1200 mL) and at three points during the 30-km TT (7.5, 15, and 22.5 km; total: 450 mL)	0.23 [−0.75, 1.21]
Below et al., 1995 [55]	CS	8/M	23.0	22.0	62.9	Lab	50 min of cycling at 80 ± 1% $\dot{V}$O_2_max (± SEM) + ~10 min performance test: time to complete amount of work at intensity maintained a $\dot{V}$O_2_ of 10% above his LT, TA: 31.2 °C	6% CHO-E solution (Gatorade, Quaker Oats Co.) during exercise, (1330 ± 60 mL)	0.77 [−0.26, 1.80]
Bishop et al., 2001 [56]	CS	9/M	21.0	25.3	53.1	Lab	TF cycling at 75% $\dot{V}$O_2_max, TA: 19.3 °C	5% CHO-E (GL) beverage, before (5 mL·kg^−1^) and at 15-min intervals (2 mL·kg^−1^) during exercise	0.89 [−0.09, 1.87]
Burgess et al., 1991 [57]	CS	9/M	24.0−30.0	#	59.9 *	Lab	165 min of cycling at 70% $\dot{V}$O_2_max + TE at 80% $\dot{V}$O_2_max, TA: 22.0 °C	1.8% CHO-E solution (SUC 18 g·L^−1^), 3.5 mL·kg^−1^ at the 20th min and every 20 min in a total of 160 min of exercise	0.18 [−0.74, 1.11]
Byrne et al., 2005 [58]	CS	14/M	20.7	22.1	53.0	Lab	3 × 60 min cycles of loaded marching at 4.4 km·h^−1^ and 5% gradient, separated by 15 min rest, TA: 35.0 °C	5.8% CHO-E fluid (5.8 g·100 mL^−1^: Gatorade Quaker, Chicago, IL, USA), 5 mL·kg^−1^ prior to exercise, followed by 3 mL·kg^−1^ every 15 min during exercise and rest periods	0.22 [−0.52, 0.97]
Campbell et al., 2008 [59]	CS	16/8 M 8 F	35.8 M and 32.4 F	23.6 M and 22.2 F	59.3 * M and 50.2 * F	Lab	80 min of cycling at 75% $\dot{V}$O_2_peak + a 10 km TT, TA: 19.0–23.0 °C	5.9% CHO-E drink, {per 8-oz serving or 0.6 g·kg^−1^·h^−1^, [(SUC:GL-FRU mix), 14 g]}	0.19 [−0.50, 0.89]
Carter et al., 2003 i [60]	CS	7/M	22.6	23.3	59.5	Lab	TF cycling at 60% $\dot{V}$O_2_max, TA: 35.0 °C	6.4% CHO-E solution, MD 0.51 g·kg^−1^ (8 mL·kg^−1^, 5 min prior to exercise) and at 15-min intervals (3 mL·kg^−1^) during exercise	0.70 [−0.39, 1.79]
Carter et al., 2003 ii [60]	CS	8/M	22.6	23.3	59.5	Lab	TF cycling at 73% $\dot{V}$O_2_max, TA: 35.0 °C	6.4% CHO-E solution, MD 0.51 g·kg^−1^ (8 mL·kg^−1^, 5 min prior to exercise) and at 15-min intervals (3 mL·kg^−1^) during exercise	0.38 [−0.61, 1.37]
Carter et al., 2005 i [61]	CS	8/M	24.0	23.2	60.5	Lab	TE cycling at 60% $\dot{V}$O_2_max, TA: 35.0 °C	6.4% CHO solution (sweetened, MD), 8 mL·kg^−1^ 5 min prior to exercise and at 15-min intervals 3 mL·kg^−1^ during exercise	0.49 [−0.51, 1.49]
Carter et al., 2005 ii [61]	CS	8/M	24.0	23.2	60.5	Lab	TE cycling at 60% $\dot{V}$O_2_max, TA: 35.0 °C	6.4% CHO solution (non-sweetened, MD), 8 mL·kg^−1^ 5 min prior to exercise and at 15-min intervals 3 mL·kg^−1^ during exercise	0.44 [−0.56, 1.43]
Clark et al., 2000 [62]	PS	7/#M #F	23.0−26.0	24.3	64.0 *	Lab	km cycling TT	7.6% CHO solution (GL-polymer), 8 mL·kg^−1^ 30 min before, 2 mL·kg^−1^ 2 min before and at 10, 20, and 30 km of the TT	0.00 [−1.05, 1.05]
Clarke et al., 2011 [63]	CS	12/M	25.0	22.8	61.3	Lab	2 × 45 min of various soccer-specific running intensities on a motorized treadmill, +3 min self-chosen pace test and test of high-intensity exercise capacity (Cunningham and Faulkner test), TA: 30.5 °C	6.6 % CHO-E solution (Still Lucozade Sport, GlaxoSmithKline, Gloucestershire, UK) at 0, 15, 30, 45, 60, and 75 min of exercise (223 ± 7 mL at each time point)	2.90 [1.70, 4.10]
Cole et al., 1993 i [64]	CS	10/M	28.0	24.0	59.6	Lab	105 min of cycling at 70% $\dot{V}$O_2_max + 15 min all out ride performance, TA: 23.1 °C	6% CHO-E solution (G:SUC) at 15-min intervals (9.75 mL·kg^−1^·h^−1^)	0.23 [−0.65, 1.11]
Cole et al., 1993 ii [64]	CS	10/M	28.0	24.0	59.6	Lab	5 min of cycling at 70% $\dot{V}$O_2_max + 15 min all out ride performance, TA: 23.1 °C	8.3% CHO-E syrup (high FRU corn) at 15-min intervals (9.75 mL·kg^−1^·h^−1^)	0.22 [−0.66, 1.10]
Cole et al., 1993 iii [64]	CS	10/M	28.0	24.0	59.6	Lab	105 min of cycling at 70% $\dot{V}$O_2_max + 15 min all out ride performance, TA: 23.1 °C	8.3% CHO-E solution (6% high FRU corn syrup + 2.3% GL-polymer) at 15-min intervals (9.75 mL·kg^−1^·h^−1^)	0.21 [−0.67, 1.09]
Cole et al., 2018 [65]	CS	14/M	42.6	23.7	57.6	Lab	120 min of cycling at a submaximal exercise intensity (60% Maximal Minute Power), TA: 19.6 °C	6% CHO solution (MD 18 g·300 mL^−1^ of water: Blackburn Distributions, Blackburn, UK) every 30 min during exercise	3.96 [2.62, 5.30]
Coletta et al., 2013 i [66]	CS	12/M	18.0−55.0	22.7	59.7	Field	19.2 km run at a race pace + 1.92 km sprint to the finish	6% CHO (Gatorade, Inc., Chicago, IL, USA) of 120 mL servings 5 min before the start, and every 4 km throughout the run (total: 600 mL)	0.08 [−0.72, 0.88]
Coletta et al., 2013 ii [66]	CS	12/M	18.0−55.0	22.7	59.7	Field	19.2 km run at a race pace + 1.92 km sprint to the finish	7.4% CHO (Gatorade, Inc., Chicago, IL, USA) of 120 mL servings 5 min before the start, and every 4 km throughout the run (total: 600 mL)	−0.07 [−0.87, 0.73]
Cureton et al., 2007 [67]	CS	16/M	27.5	23.2	60.5	Lab	120 min of cycling at intensity between 60% and 75% $\dot{V}$O_2_max every 15 min + 15 min all out ride performance, TA: 28.5 °C	6% CHO-E fluid (Gatorade®, Quaker Oats Co., Barrington, IL, USA), 6 mL·kg^−1^ (10 min before and immediately) prior to exercise and 3 mL·kg^−1^ every 15 min intervals over the first 105 min of exercise	0.35 [−0.35, 1.05]
Currell et al., 2008 i [68]	CS	8/M	32.0	#	64.7	Lab	120 min of cycling exercise at 55% Wmax + a TT to complete a set amount of work as quickly as possible (~60 min), TA: 20.0–23.0 °C	14.4% GL beverage (1.8 g·min^−1^), 600 mL prior to exercise and 150 mL every 15 min throughout the SS period and at 25, 50, and 75% of the TT	2.55 [1.14, 3.97]
Currell et al., 2008 ii [68]	CS	8/M	32.0	#	64.7	Lab	120 min of cycling exercise at 55% Wmax + a TT to complete a set amount of work as quickly as possible (~60 min), TA: 20.0–23.0 °C	14.4% GL:FRU beverage in a ratio of 2:1 (1.8 g·min^−1^), 600 mL prior to exercise and 150 mL every 15 min throughout the SS period and at 25, 50, and 75% of the TT	4.37 [2.37, 6.37]
Davis et al., 2000 [69]	CS	8/M	27.1	#	55.0 *	Lab	10 min warm up, 5 × 15 min bouts of intermittent shuttle running (at 95 and 55% of $\dot{V}$O_2_max separated by 3 min rest) + 1 bout of intermittent shuttle running to fatigue	6% CHO-E drink (5 mL·kg^−1^, CHO 60 g·L^−1^) 10 min prior to exercise and at 15-min intervals	0.49 [−0.51, 1.49]
Desbrow et al., 2004 [70]	CS	9/M	30.0	#	65.1 *	Lab	amount of cycling work (14 kJ·kg^−1^) as fast as possible (equal to ~60 min at ~75% Wmax), TA: 22.0 °C	6% CHO-E drink (Gatorade, Quaker Oats Co.), 8 mL·kg^−1^ prior to exercise and 2 mL·kg^−1^ between 20–30%, 50–60%, 70–80% of the total amount of work	0.02 [−0.90, 0.95]
Duhamel et al., 2007 [71]	CS	15/14 M 1 F	19.3	24.5	44.0 *	Lab	TF cycling at ~60% $\dot{V}$O_2_peak, TA: 20.0 °C	6% CHO-E solution (CHO > 1 g·kg^−1^, 100–300 mL) after 30 min of exercise and every 15 min thereafter, served at 20.0 °C	0.85 [0.10, 1.60]
El-Sayed et al., 1995 [72]	CS	9/M	23.8	22.2	60.7	Lab	60 min of continuous cycling at the external workload predicted to elicit 70% $\dot{V}$O_2_max + a 10 min self-paced, all-out effort performance ride, TA: 22.0 °C	7.5% GL solution (3 mL·kg^−1^) at 15 min prior to exercise and at 20-min intervals (3 mL·kg^−1^) during the submaximal exercise	0.57 [−0.38, 1.52]
El-Sayed et al., 1997 [73]	CS	8/M	24.0	21.9	66.5 *	Lab	60 min of simulated cycling TT at a self-selected maximal pace, TA: 22.0 °C	8% GL solution (4.5 mL·kg^−1^) prior to exercise	0.16 [−0.82, 1.14]
Finger et al., 2018 [74]	CS	13/M	29.7	23.1	62.2	Field	running 10 km + cycling 40 km + 5 km running (TT 5 km) at a self-selected pace, TA: 18.0–22.0 °C	75 g of MD diluted in 450 mL of cold water, doses of 150 mL at kilometers 5, 20, and 35 of the cycling section	0.04 [−0.73, 0.81]
Fulco et al., 2007 i [75]	PS	9/#M #F	30.0−30.7	23.5	43.4 *	Lab	amount of cycling work (720 kJ) as fast as possible (~60 min of cycling at 4300 m altitude while living at the same altitude for 1 days and acclimatization ~2 years at 2000 m)	10% CHO solution [(mass/volume, 9%) MD + 2% GL + 1% FRU: Ergo Drink. U.S. Army Soldier Systems Command, Natick, MA)] at the start of the exercise every 15 min thereafter (0.175 g·kg^−1^)	0.58 [−0.48, 1.64]
Fulco et al., 2007 ii [75]	PS	9/#M #F	30.0−30.7	23.5	43.4 *	Lab	amount of cycling work (720 kJ) as fast as possible (~60 min of cycling at 4300 m altitude while living at the same altitude for 3 days acclimatization ~2 years at 2000 m)	10% CHO solution [(mass/volume, 9%) MD + 2% GL + 1% FRU: Ergo Drink. U.S. Army Soldier Systems Comm, Natick, MA, USA)] at the start of the exercise every 15 min thereafter (0.175 g·kg^−1^)	0.43 [−0.62, 1.48]
Funnell et al., 2017 i [76]	CS	16/M	23.0	23.5	54.2	Lab	3 blocks of the LIST (simulated soccer performance, totaling 45 min), each block (15 min) consisted of ~11 repeated cycles of walking (three shuttles at 1.5 m·s^−1^), sprinting (15 m), rest (4 s), jogging (three shuttles at 55% predicted $\dot{V}$O_2_max) cruising (three shuttles at 95% predicted $\dot{V}$O_2_max), 3rd block was “self-selected” intensity distance was recorded	12% CHO-E solution (SUC:MD:IsoMAL), 250 mL before the LIST	−0.28 [−0.97, 0.42]
Funnell et al., 2017 ii [76]	CS	16/M	23.0	23.5	54.2	Lab	6 blocks of the LIST (simulated soccer performance, totaling 90 min), each block (15 min) consisted of ~11 repeated cycles of walking (three shuttles at 1.5 m·s^−1^), sprinting (15 m), rest (4 s), jogging (three shuttles at 55% predicted $\dot{V}$O_2_max) cruising (three shuttles at 95% predicted $\dot{V}$O_2_max), sixth block was “self-selected” intensity distance was recorded	12% CHO-E solution (SUC:MD:IsoMAL), 250 mL before at half-time of the LIST	−0.23 [−0.92, 0.47]
Ganio et al., 2010 [77]	CS	14/M	27.0	22.8	60.4	Lab	120 min of submaximal cycling (alternating 61 ± 5% 75 ± 5% $\dot{V}$O_2_max) + a 15-min maximal effort cycling (pedal revolutions increased linearly), TA: 28.7 °C	6% CHO-E solution (Gatorade, Quaker Oats Co., Barrington, IL, USA) before (6 mL·kg^−1^) every 15 min during exercise (3 mL·kg^−1^)	0.42 [−0.33, 1.17]
Glace et al., 2019 i [78]	CS	10/M	35.0	#	60.3 *	Lab	120 min of cycling at VT with interspersed higher-intensity intervals + a 3 km TT	5.9% CHO-E (Gatorade, PepsiCo, Purchase, NY, USA) at a rate of 1% of BM each h (male subjects)	0.28 [−0.60, 1.16]
Glace et al., 2019 ii [78]	CS	10/F	42.0	#	46.5 *	Lab	120 min of cycling at VT with interspersed higher-intensity intervals + a 3 km TT	5.9% CHO-E (Gatorade, PepsiCo, Purchase, NY, USA) at a rate of 1% of BM each h (female subjects)	0.38 [−0.51, 1.26]
Glace et al., 2019 iii [78]	CS	10/M	35.0	#	60.3 *	Lab	120 min of cycling at VT with interspersed higher-intensity intervals + a 3 km TT + a ride to exhaustion at RCT	5.9% CHO-E (Gatorade, PepsiCo, Purchase, NY, USA) at a rate of 1% of BM each h (male subjects)	0.38 [−0.51, 1.26]
Glace et al., 2019 iv [78]	CS	10/F	42.0	#	46.5 *	Lab	120 min of cycling at VT with interspersed higher-intensity intervals + a 3 km TT + a ride to exhaustion at RCT	5.9% CHO-E (Gatorade, PepsiCo, Purchase, NY, USA) at a rate of 1% of BM each h (female subjects)	−0.02 [−0.89, 0.86]
Goedecke et al., 2013 [79]	CS	22/M	24.0	25.0	51.8	Lab	(2 × Illinois agility run) + SSM (LIST ~90 min) + Illinois agility run + timed run to fatigue (20 m runs at progressively increasing speeds at the point where two consecutive shuttles could not be completed)	7% CHO-E drink (SUC: Energade, Tiger Consumer Br&s Ltd., Bryanston, Johannesburg, South Africa), 250 mL prior to the warm-up and following the 3rd 15-min exercise bout and 50 mL during the 90 sec-break separating each 15-min exercise bout (total: 700 mL, CHO 49 g)	0.39 [−0.21, 0.99]
Gui et al., 2017 [80]	CS	11/F	32.4	21.0	49.0	Lab	5 km at 70% $\dot{V}$O_2_max + 16 km performance run, TA: 22.0 °C	6% CHO-E (SUC: Coca-Cola, HK), 150 mL at 0 km and every 2.5 km (CHO ingestion rate ~36 g·h^−1^)	0.47 [−0.38, 1.32]
Heesch et al., 2014 i [81]	CS	8/M	34.5	24.8	56.8	Lab	cycling at 60% of the Wmax for 120 min + a 10 km cycling performance trial, TA: 21.5 °C	3% CHO solution (MD), 250 mL every 15 min during the 2-hr cycling bout	0.28 [−0.71, 1.26]
Heesch et al., 2014 ii [81]	CS	8/M	34.5	24.8	56.8	Lab	cycling at 60% of the Wmax for 120 min + a 10 km cycling performance trial, TA: 21.5 °C	6% CHO solution (MD), 250 mL every 15 min during the 1st h, followed by 250 mL of an artificially sweetened placebo beverage every 15 min during the 2nd h of cycling	0.17 [−0.81, 1.15]
Heesch et al., 2014 iii [81]	CS	8/M	34.5	24.8	56.8	Lab	cycling at 60% of the Wmax for 120 min + a 10 km cycling performance trial, TA: 21.5 °C	artificially sweetened placebo beverage of a 250 mL every 15 min during the 1st h, followed by 250 mL of an 6% CHO solution (MD) every 15 min during the 2nd h of cycling	0.31 [−0.68, 1.30]
Hulston & Jeukendrup 2008 [82]	CS	10/M	27.0	#	65.7	Lab	105 min of SS cycling at 62% $\dot{V}$O_2_max followed by a TT to complete a set amount of work (688 ± 56 kJ) as fast as possible	6.4% CHO solution (GL), 5.5 mL·kg^−1^ at the onset of exercise and 2 mL·kg^−1^ at subsequent 15-min intervals until completion of the SS exercise	0.50 [−0.40, 1.39]
Hulston & Jeukendrup 2009 [83]	CS	10/M	28.0	#	61.7	Lab	120 min of SS cycling at 61% $\dot{V}$O_2_max followed by a TT to complete a set amount of work (847 ± 78 kJ) as fast as possible	6% CHO-E solution (GL:FRU in a ratio of 2:1), 600 mL at the onset of exercise and 150 mL every 15 min thereafter	1.35 [0.36, 2.35]
Ivy et al., 1983 [84]	CS	10/M	23.8	23.3	60.4	Lab	TE walking at 45% $\dot{V}$O_2_max	20% GL-polymer solution (Polycose, Ross Laboratories, Columbus, OH), (2 kcal·mL^−1^), in four equally divided dosages 60, 90, 120, and 150 min following the start of exercise, (total: GL-polymer 120 g), served at 4.0 °C	0.86 [−0.07, 1.78]
Jarvis et al., 1999 [85]	CS	10/F	30.4	22.4	47.1	Lab	50 min of cycle ergometry at 80% $\dot{V}$O_2_max + WAT	7% CHO solution (GL-polymer containing MD: Exceed), 2 mL·kg^−1^ BM at 10, 20, 30, and 40-min intervals throughout the 50 min of exercise	0.08 [−0.79, 0.96]
Jeukendrup et al., 1997 [86]	CS	19/17Μ 2 F	23.0 M and 21.0 F	31.3 M and 18.8 F	72.9 M and 64.2 F	Lab	amount of cycling work (0.75 × Wmax × 3600) as fast as possible (~60 min), TA: 20.0 °C	7.6% CHO-E solution (Isostar, Sandoz Nutrition, Switzerl&), 8 mL·kg^−1^ at the 5-min warm-up period and 2 mL·kg^−1^ upon achievement of 25%, 50% and 75% of the work set	0.54 [−0.11, 1.19]
Kang et al., 1995 [87]	CS	7/M	23.0	23.3	61.6 *	Lab	TE cycling at 71 ± 1% $\dot{V}$O_2_max	6% GL-SUC solution (Gatorade, Quaker Oats Co.) every 20 min at a rate of 0.6 g·kg^−1^·h^−1^	0.61 [−0.47, 1.69]
Kang et al., 1996 [88]	CS	7/M	23.0	23.3	61.2 *	Lab	TE cycling at 70% $\dot{V}$O_2_max	6% GL-SUC solution (Gatorade, Quaker Oats Co.) every 20 min at a rate of 0.6 g·kg^−1^·h^−1^	0.48 [−0.59, 1.55]
Khanna & Manna 2005 [89]	CS	10/M	25.8	21.0	54.4	Lab	TE running at 70% $\dot{V}$O_2_max, TA: 25.0 °C	5% CHO-E solution, 100 mL at 15-min intervals until completion of the exercise	2.10 [0.96, 3.24]
King et al., 2018 i [90]	CS	10/M	30.7	#	61.6	Lab	120 min of cycling at 77% $\dot{V}$O_2_max + a 30-min self-paced TT	CHO-E, CHO ingestion rate 60 g·h^−1^, (D-GL; Thornton & Ross Ltd., Huddersfield, UK), 250 mL solution every 15 min, starting at 15-min into the exercise protocol	0.43 [−0.46, 1.32]
King et al., 2018 ii [90]	CS	10/M	30.7	#	61.6	Lab	120 min of cycling at 77% $\dot{V}$O_2_max + a 30-min self-paced TT	CHO-E, CHO ingestion rate 75 g·h^−1^, (D-GL; Thornton & Ross Ltd., Huddersfield, UK), 250 mL solution every 15 min, starting at 15 min into the exercise protocol	0.19 [−0.69, 1.07]
King et al., 2018 iii [90]	CS	10/M	30.7	#	61.6	Lab	120 min of cycling at 77% $\dot{V}$O_2_max + a 30-min self-paced TT	CHO-E, CHO ingestion rate 90 g·h^−1^, GL:FRU in a ratio of 2:1 (GL:D-GL; Thornton & Ross Ltd., Huddersfield, UK & FRU: Danisco, Kettering, UK), 250 mL solution every 15 min, starting at 15 min into the exercise protocol	0.83 [−0.10, 1.75]
King et al., 2018 iv [90]	CS	10/M	30.7	#	61.6	Lab	120 min of cycling at 77% $\dot{V}$O_2_max + a 30-min self-paced TT	CHO-E, GL:FRU in a ratio of 2:1, CHO ingestion rate 112.5 g·h^−1^ (GL:D-GL; Thornton & Ross Ltd., Huddersfield, UK & FRU: Danisco, Kettering, UK), 250 mL solution every 15 min, starting at 15 min into the exercise protocol	0.58 [−0.32, 1.48]
King et al., 2019 i [91]	CS	11/M	30.3	24.2	60.0	Lab	180 min of cycling at 60% $\dot{V}$O_2_max + a 30-min self-paced TT	CHO-E, GL:FRU in a ratio of 2:1, CHO ingestion rate 80 g·h^−1^ (GL:D-GL; Thornton & Ross Ltd., Huddersfield, UK & FRU: Danisco, Kettering, UK), 250 mL solution every 15 min, starting at 15 min into the exercise protocol	0.92 [0.03, 1.81]
King et al., 2019 ii [91]	CS	11/M	30.3	24.2	60.0	Lab	180 min of cycling at 60% $\dot{V}$O_2_max + a 30-min self-paced TT	CHO-E, GL:FRU in a ratio of 2:1, CHO ingestion rate 90 g·h^−1^ (GL:D-GL; Thornton & Ross Ltd., Huddersfield, UK & FRU: Danisco, Kettering, UK), 250 mL solution every 15 min, starting at 15 min into the exercise protocol	1.09 [0.18, 2.00]
King et al., 2019 iii [91]	CS	11/M	30.3	24.2	60.0	Lab	180 min of cycling at 60% $\dot{V}$O_2_max + a 30-min self-paced TT	CHO-E, GL:FRU in a ratio of 2:1, CHO ingestion rate 100 g·h^−1^ (GL:D-GL; Thornton & Ross Ltd., Huddersfield, UK & FRU: Danisco, Kettering, UK), 250 mL solution every 15 min, starting at 15 min into the exercise protocol	0.58 [−0.27, 1.44]
Kingwell et al., 1989 [92]	CS	9/M	23.0	#	74.1	Lab	160 min of cycling at 65% $\dot{V}$O_2_max + 5 min rest + TE at 110% $\dot{V}$O_2_max, TA: 20.0–22.0 °C	10% GL-polymer solution (Polycose), 200 mL at the start of exercise and at 20-min intervals thereafter	0.53 [−0.42, 1.47]
Learsi et al., 2019 i [93]	CS	9/M	28.0	24.9	41.2	Lab	105 min of constant-load cycling (50% of the difference between the 1st and 2nd LT) + a 10 km TT, TA: 21.0 °C	8% CHO beverage (MD, 2 mL·kg^−1^ of BM: Neonutri, Poços de Caldas, Brazil) immediately prior to exercise, every 15 min throughout the constant-load exercise and at the 5th km point during the 10-km TT	0.89 [−0.09, 1.87]
Learsi et al., 2019 ii [93]	CS	9/M	28.0	24.9	41.2	Lab	105 min of constant-load cycling (50% of the difference between the 1st and 2nd LT) + a 10 km TT, TA: 21.0 °C	8% CHO beverage (MD, 2 mL·kg^−1^ of BM: Neonutri, Poços de Caldas, Brazil) immediately prior to exercise, every 15 min throughout the constant-load exercise and at the 5th km point during the 10-km TT (overnight fast trial)	2.31 [1.05, 3.57]
Lugo et al., 1993 [94]	CS	9/M	23.0	#	63.7 *	Lab	120 min of cycling at 70% $\dot{V}$O_2_peak + 5 min rest + TT distance (calculated as the distance traveled if 80% $\dot{V}$O_2_peak was maintained for 30 min), TA: 22.0 °C	7% CHO-E solution (Fluid Replacement Energy Drinks, Ross Laboratories, Columbus, OH), 0.4 g·kg^−1^ at 0 min and every 30 min until the 120th min	0.88 [−0.10, 1.86]
Martínez-Lagunas et al., 2010 [95]	CS	12/7 M 5 F	28.3	22.1	63.2 M and 49.2 F	Lab	cycling at varied intensities, 55–75% $\dot{V}$O_2_max for 150 min + at 80% $\dot{V}$O_2_max until fatigued, TA: 21.0–23.0 °C	6% CHO-E solution, 0.7 g·kg^−1^·h^−1^, every 20 min (255.4 ± 9.1 mL)	0.69 [−0.14, 1.52]
Maughan et al., 1989 [96]	CS	6/M	29.0	22.3	53.0	Lab	TE cycling at 70% $\dot{V}$O_2_max	4% CHO solution (GL-E: Dioralyte, Rorer Health Care Ltd., Eastbourne, UK), 100 mL at 0-min and at 10-min intervals	0.51 [−0.65, 1.66]
McConell et al., 1996 [97]	CS	8/M	23.0	#	69.2 *	Lab	120 min of cycling at 70 ± 1% $\dot{V}$O_2_peak, + 15 min all out performance, TA: 20.0–22.0 °C	7% CHO-E drink (Sport Plus, Cadbury Schweppes Pty. Ltd., Melbourne, Australia), 250 mL at 0 min and at 15-min intervals until the 120th min	1.02 [−0.04, 2.08]
McConell et al., 1999 [98]	CS	8/M	22.0	#	66.9 *	Lab	TE cycling at 69 ± 1% $\dot{V}$O_2_peak	8% CHO solution, 250 mL immediately prior to exercise and at 15-min intervals thereafter	0.97 [−0.08, 2.03]
McConell et al., 2000 [99]	CS	13/M	24.0	23.0	65.7	Lab	TE cycling at 83 ± 1% $\dot{V}$O_2_peak, TA: 19.0–22.0 °C	6% CHO (D-GL) solution, 7 mL·kg^−1^ immediately prior to exercise and 3.5 mL·kg^−1^ at 15-min intervals thereafter	−0.08 [−0.85, 0.69]
McGawley et al., 2012 [100]	CS	10/6 M 4 F	25.0 M and 26.0 F	#	62.9 * M and 61.9 * F	Lab	simulated Olympic-distance triathlon with 3-min transition period between sections (1500 m swimming + 40 km cycling at 75% of MAP were of fixed intensity while the 10 km run section was completed as a TT), TA: 15.9 °C	14.4% CHO-E solution (MD:FRU in a ratio of 2:1: EnergySource Fresh Citrus flavor, H5 Ltd., Leicestershire, UK), 202 ± 20 mL 2 min prior to completing every quarter of the cycle section	0.40 [−0.48, 1.29]
Millard-Stafford et al., 1990 [101]	CS	10/M	29.6	24.3	67.0	Lab	simulated triathlon: 1.5 km swimming + 40 km cycling + 10 km running, TA: 30.0 °C	7% CHO-E solution (5% GL-polymer + 2% FRU: Exceed, Ross Laboratories, Columbus, OH), 2 mL·kg^−1^ following the swim, at 8-km intervals during cycling, and at 3.2-km intervals during running	0.14 [−0.74, 1.02]
Millard-Stafford et al., 2005 i [102]	CS	10/M	23.7	21.2	76.9	Field	the final 5 km of a 32-km run at 25.6 °C	6% CHO-E beverage (SUC:GL: Gatorade, The Quaker Oats Co., Chicago, IL, USA), 400 mL 15 min prior to exercise and 250 mL every 5 km thereafter	0.50 [−0.39, 1.40]
Millard-Stafford et al., 2005 ii [102]	CS	10/M	23.7	21.2	76.9	Field	the final 5 km of a 32-km run at 25.6 °C	8% CHO-E beverage (3.5% FRU + 2.5%GL + 2% MD: PowerAde, The Coca-Cola Co., Atlanta, GA), 400 mL 15 min prior to exercise and 250 mL every 5 km thereafter	0.62 [−0.29, 1.52]
Millard-Stafford et al., 2007 [103]	CS	16/M	27.5	23.2	60.5	Lab	120 min of cycling at fixed intensities alternating between 60% and 75% $\dot{V}$O_2_max every 15 min + 15 min ride as hard as possible, TA: 28.5 °C	6% CHO-E beverage (Gatorade, Quaker Oats Co, Barrington, IL, USA), 6 mL·kg^−1^ 10 min before, 6 mL·kg^−1^ at onset of exercise and 3 mL·kg^−1^ at 15-min intervals	0.34 [−0.36, 1.04]
Morris et al., 2003 [104]	CS	9/M	23.3	24.7	57.3	Field	5 × 15 min set (walking and variable speed running) each separated by 4 min rest + 60 s run and 60 s rest until exhaustion, TA: 30.0 °C	6.5% CHO-E solution (DEX:MD:GL: Lucozade Sport, SmithKline Beecham), 6.5 mL·kg^−1^ prior to exercise and 4.5 mL·kg^−1^ during every exercise set and rest period (19 min)	−0.31 [−1.24, 0.62]
Murray et al., 1987 i [105]	CS	13/M	30.6	24.6	45.1	Lab	intermittent SS cycling (at 55 and 65% $\dot{V}$O_2_max) interspersed with 5 rest periods (3–15 min) and 2 brief, high-intensity performance rides (timed 240 and 480 revolution cycling task), TA: 33.0 °C	5% CHO drink (GL-polymer), 2 mL·kg^−1^ during each of the five rest periods	0.63 [−0.16, 1.42]
Murray et al., 1987 ii [105]	CS	13/M	30.6	24.6	45.1	Lab	intermittent SS cycling (at 55 and 65% $\dot{V}$O_2_max) interspersed with 5 rest periods (3–15 min) and 2 brief, high-intensity performance rides (timed 240 and 480 revolution cycling task), TA: 33.0 °C	6% CHO-E drink (4.0% SUC + 2.0% GL), 2 mL·kg^−1^ during each of the five rest periods	1.13 [0.29, 1.97]
Murray et al., 1987 iii [105]	CS	13/M	30.6	24.6	45.1	Lab	intermittent SS cycling (at 55 and 65% $\dot{V}$O_2_max) interspersed with 5 rest periods (3–15 min) and 2 brief, high-intensity performance rides (timed 240 and 480 revolution cycling task), TA: 33.0 °C	7% CHO-E drink (5.0% GL-polymer + 2.0% FRU), 2 mL·kg^−1^ during each of the five rest periods	1.49 [0.60, 2.37]
Murray et al., 1989 i [106]	CS	12/7 M 5 F	30.7	23.1	42.8	Lab	3 × 20 min of cycling at 65% $\dot{V}$O_2_max each separated by 5 min rest between each bout + time to complete 1200 pedal revolutions as fast as possible (workload: 65% $\dot{V}$O_2_max at 60 rpm), TA: 33.4 °C	6% CHO-E drink (SUC), 2.5 mL·kg^−1^ prior to exercise and during each of the three rest periods, served at 8.0 °C	0.57 [−0.25, 1.39]
Murray et al., 1989 ii [106]	CS	12/7 M 5 F	30.7	23.1	42.8	Lab	3 × 20 min of cycling at 65% $\dot{V}$O_2_max each separated by 5 min rest between each bout + time to complete 1200 pedal revolutions as fast as possible (workload: 65% $\dot{V}$O_2_max at 60 rpm), TA: 33.4 °C	8% CHO-E drink (SUC), 2.5 mL·kg^−1^ prior to exercise and during each of the three rest periods, served at 8.0 °C	0.30 [−0.50, 1.11]
Murray et al., 1989 iii [106]	CS	12/7 M 5 F	30.7	23.1	42.8	Lab	3 × 20 min of cycling at 65% $\dot{V}$O_2_max each separated by 5 min rest between each bout + time to complete 1200 pedal revolutions as fast as possible (workload: 65% $\dot{V}$O_2_max at 60 rpm), TA: 33.4 °C	10% CHO-E drink (SUC), 2.5 mL·kg^−1^ prior to exercise and during each of the three rest periods, served at 8.0 °C	0.05 [−0.75, 0.85]
Murray et al., 1991 i [107]	CS	10/8 M 2 F	32.5	#	48.3	Lab	120 min of cycling at various intensities (65–75% $\dot{V}$O_2_max) + 4.8 km performance, TA: 10.0 °C	6% CHO solution (GL, ingestion rate 26 g·h^−1^: Grain Processing Corp., Muscatine, IA) at the 12 th min and every 15 min thereafter, served at 10.0 °C	0.60 [−0.30, 1.50]
Murray et al., 1991 ii [107]	CS	10/8 M 2 F	32.5	#	48.3	Lab	120 min of cycling at various intensities (65–75% $\dot{V}$O_2_max) + 4.8 km performance, TA: 10.0 °C	12% CHO solution (8% MD + 4% DEX, CHO ingestion rate 52 g·h^−1^: Grain Processing Corp., Muscatine, IA) at the 12th min and every 15 min thereafter, served at 10.0 °C	0.40 [−0.49, 1.29]
Murray et al., 1991 iii [107]	CS	10/8 M 2 F	32.5	#	48.3	Lab	120 min of cycling at various intensities (65–75% $\dot{V}$O_2_max) + 4.8 km performance, TA: 10.0 °C	18% CHO solution (15% MD + 3% DEX, CHO ingestion rate 78 g·h^−1^: Grain Processing Corp., Muscatine, IA) at the 12th min and every 15 min thereafter, served at 10.0 °C	0.49 [−0.40, 1.38]
Naclerio et al., 2014 [108]	CS	10/M	25.0	24.0	#	Lab	90 min of intermittent repeated running sprint test involving 4 blocks of 11 sets of 3 repetitions of 60 m at 60%, 80%, and 60% maximal aerobic speed plus 15 m sprint (IRST)	13.9% CHO solution, 500 mL containing 69.5 g of CHO (MD), immediately prior to the 1st, 2nd, 3rd, and 4th blocks of the IRST	−0.01 [−0.89, 0.87]
Nassif et al., 2014 [109]	CS	10/M	26.0	22.3	70.7	Lab	60 km cycling TT punctuated by 1-km sprints (14, 29, 44, 59 km), TA: 32.0 °C	6% CHO solution (4 mL·kg^−1^ of BM) at 5 km, after each sprint (15, 30, 45 km) and at 55 km, served at 4.0 °C	−0.66 [−1.56, 0.25]
Nassis et al., 1998 [110]	CS	9/8 M 1 F	25.0	23.5	65.1	Lab	15 s bouts of fast running (at 80% $\dot{V}$O_2_max for the 1st 60 min, at 85% $\dot{V}$O_2_max from 60 to 100 min of exercise and finally at 90% $\dot{V}$O_2_max from 100 min of exercise until exhaustion) separated by 10 s of slow running (at 45% $\dot{V}$O_2_max), TA: 21.6 °C	6.9% CHO-E solution served at 8.0–9.0 °C (Lucozade-Sport, SmithKline Beecham Coleford, Glos, UK) immediately before the run (3 mL·kg^−1^) and every 20 min thereafter (2 mL·kg^−1^)	−0.10 [−1.02, 0.83]
Newell et al., 2015 i [111]	CS	20/M	34.0	23.5	62.0	Lab	120 min of constant-load ride at 95% of LT (185 ± 25 W) + a work-matched TT task (~30 min at 70% of PPO), TA: 19.0 °C	2.0% CHO-E drink (GL-monomers-polymers) at a rate of 1 L·h^−1^ (240 mL 2 min prior to exercise, subsequently 220 mL every 15 min with the final drink provided at the 120th min of exercise), served at 10.0 °C	0.34 [−0.28, 0.97]
Newell et al., 2015 ii [111]	CS	20/M	34.0	23.5	62.0	Lab	120 min of constant-load ride at 95% of LT (185 ± 25 W) + a work-matched TT task (~30 min at 70% of PPO), TA: 19.0 °C	3.9% CHO-E drink (GL-monomers-polymers) at a rate of 1 l·h^−1^ (240 mL 2 min prior to exercise, subsequently 220 mL every 15 min with the final drink provided at the 120th min of exercise), served at 10.0 °C	0.59 [−0.05, 1.22]
Newell et al., 2015 iii [111]	CS	20/M	34.0	23.5	62.0	Lab	120 min of constant-load ride at 95% of LT (185 ± 25 W) + a work-matched TT task (~30 min at 70% of PPO), TA: 19.0 °C	6.4% CHO-E drink (GL-monomers-polymers) at a rate of 1 L·h^−1^ (240 mL 2 min prior to exercise, subsequently 220 mL every 15 min with the final drink provided at the 120th min of exercise), served at 10.0 °C	0.62 [−0.02, 1.25]
Nicholas et al., 1995 [112]	CS	9/M	24.8	24.9	56.3	Field	5 × 15 min periods of intermittent running, consisting of sprinting, interspersed with periods of jogging and walking + intermittent running to fatigue (corresponding to 55 and 95% $\dot{V}$O_2_max, alternating every 20 m), TA: 20.0 °C	6.9% CHO-E solution (Lucozade-Sport, SmithKline Beecham, Brentford, UK) immediately prior to exercise (5 mL·kg^−1^) and every 15 min thereafter (2 mL·kg^−1^)	0.55 [−0.40, 1.49]
Nikolopoulos et al., 2004 [113]	CS	8/M	25.0	#	66.0	Lab	TE cycling at 84 ± 1% $\dot{V}$O_2_max	6.4% CHO-E solution, 10 min prior to exercise (8 mL·kg^−1^) and every 15 min thereafter (2 mL·kg^−1^)	0.34 [−0.65, 1.33]
Nishibata et al., 1993 [114]	CS	7/M	27.0	22.5	50.3	Lab	TE cycling at 73.4 ± 7.7% $\dot{V}$O_2_max	10% GL-drink 15 min prior to exercise and at the 15th min and 45th min of exercise equal amounts, chilled with ice (total: GL 43.1 ± 4.2 g)	−0.20 [−1.25, 0.85]
Oosthuyse et al., 2015a i [115]	CS	9/M	38.0	24.2	60.8	Lab	120 min of SS cycling (60% Wmax) + a 16 km TT, TA: 17.0–19.0 °C	7% CHO-E solution equal to 900 mL·h^−1^ and CHO ingestion rate 63 g·h^−1^ of 0.8:1 FRU:MD (Krystar 300 crystalline FRU, Tate & Lyle, Decatur, IL, USA & Glucidex 12, Roquette Frères, Lestrem Cedex, France)	0.07 [−0.86, 0.99]
Oosthuyse et al., 2015a ii [115]	CS	9/M	38.0	24.2	60.8	Lab	120 min of SS cycling (60% Wmax) + a 16 km TT, TA: 17.0–19.0 °C	7% CHO-E solution equal to 900 mL·h^−1^ and CHO ingestion rate 63 g·h^−1^ of IsoMAL (Palatinose, Beneo-Palatinit GmbH, Mannheim, Germany)	−0.31 [−1.24, 0.62]
Oosthuyse et al., 2015b [116]	CS	8/M	38.9	24.3	60.9	Lab	120 min of SS cycling (60% Wmax) + a 16 km TT, TA: 19.2 °C	7% CHO-E solution equal to 900 mL·h^−1^ and CHO ingestion rate 63 g·h^−1^ at a ratio of 0.8:1 FRU:MD (Krystar 300 crystalline FRU, Tate & Lyle, Decatur, IL, USA & Glucidex 12, Roquette Frères, Lestrem Cedex, France), 400 mL before commencing exercise, 200 mL at every 15 min	0.15 [−0.83, 1.14]
Osterberg et al., 2008 [117]	CS	13/M	31.2	22.6	56.0 *	Lab	120 min of constant-load cycling at a workload 5% below OBLA followed by a TT to complete a set amount of work (7 kJ·kg^−1^) as quickly as possible, TA: 23.0 °C	6% CHO-E beverage (2% SUC + 2% GL + 2% FRU: Gatorade Thirst Quencher, The Gatorade Company, Chicago, IL, USA), 250 mL every 15 min during the constant-load ride	0.60 [−0.19, 1.39]
O’Hara et al., 2014 i [118]	CS	10/M	31.0	#	58.1	Lab	4 × 5 min of continuous progressive workload increments corresponding to 70, 75, 80, and 85% Wmax + 10 × 90 s sprints at 90% Wmax separated by 180 s recovery at 55% Wmax + cycling to volitional exhaustion at 90% Wmax, TA: 18.0 °C	4% CHO solution, 40 g of GAL (D-GAL: Inalco, Milan, Italy) as 1 l formulation	0.34 [−0.54, 1.22]
O’Hara et al., 2014 ii [118]	CS	10/M	31.0	#	58.1	Lab	4 × 5 min of continuous progressive workload increments corresponding to 70, 75, 80, and 85% Wmax + 10 × 90 s sprints at 90% Wmax separated by 180 s recovery at 55% Wmax + cycling to volitional exhaustion at 90% Wmax, TA: 18.0 °C	4% CHO solution, 40 g of GL (D-GL: Cargill, Manchester, UK) as 1 l formulation	−0.25 [−1.13, 0.63]
O’Neal et al., 2013 [119]	CS	36/23M 13 F	23.0	23.8	#	Lab	50 min of stationary cycling at ~60–65% of heart rate reserve (146 ± 4 bpm) interspersed with 5 rest periods of 2 min each + 3 × 30 s WAT with 2.5 min rest between tests, TA: 25.0 °C	6% CHO-E beverage in 3 equal aliquots, at minutes 0, 20, and 40 during the 60-min submaximal cycling bout (mean total beverage 847 ± 368 mL: equivalent to participant’s sweat losses), served chilled	0.10 [−0.36, 0.56]
Pettersson et al., 2019 [120]	CS	12/6 M 6 F	25.6 M and 24.8 F	#	69.1 * M and 59.9 * F	Lab	120 min of submaximal diagonal-style roller-skiing (69.3 ± 2.9% of $\dot{V}$O_2_peak) at a constant incline of 5° (treadmill speed, males: 9.7 ± 0.2 km·h^−1^, females: 8.5 ± 0.3 km·h^−1^) + final 2000 m for females or 2400 m for males TT, TA: −5.0 °C	18% CHO-E solution (MD:FRU, in a ratio of 1:0.8) with additional gelling polysaccharides (Maurten AB, Gothenburg, Sweden), ~220 mL before the onset of exercise, subsequently, 220 mL every 20 min, CHO ingestion rate 132 g·h^−1^	−0.06 [−0.86, 0.74]
Pottier et al., 2010 [121]	CS	12/#	30.2	#	61.7	Lab	amount of cycling work (0.75 × Wmax × 3600) as fast as possible (~60 min of cycling), TA: 19.0–21.0 °C	5.86% CHO-E solution (5.4% SUC + 0.46% GL: Gatorade), before and after warm-up, 2 and 1.5 mL·kg^−1^ BM respectively and after reaching each 12.5% of the total amount of work; subjects also received 1.5 mL·kg^−1^ BM	−0.16 [−0.96, 0.64]
Rilley et al., 1988 [122]	CS	9/M	30.0	22.9	65.0	Lab	TE cycling at 70–75% $\dot{V}$O_2_max	7% CHO drink (5% GL-polymer + 2% FRU: Exceed, Ross Laboratories, Columbus, OH) at 20-min intervals beginning 20 min prior to exercise (14 g CHO each treatment)	0.16 [−0.77, 1.08]
Roberts et al., 2014 i [123]	CS	14/M	31.8	23.0	60.4	Lab	150 min of continuous cycling at 50% Wmax (176.71 ± 25.92 W) + a 60 km cycling TT, TA: 22.4 °C	10% CHO-E beverage (High 5 Ltd.), MD 1.1 g·min^−1^ + FRU 0.6 g·min^−1^, 270 mL doses at the start and every 15 min (until completion of the performance trial)	0.50 [−0.47, 1.48]
Roberts et al., 2014 ii [123]	CS	14/M	31.8	23.0	60.4	Lab	150 min of continuous cycling at 50% Wmax (176.71 ± 25.92 W) + a 60 km cycling TT, TA: 22.4 °C	10% CHO-E beverage (High 5 Ltd.), MD 1.7 g·min^−1^, 270 mL doses at the start and every 15 min (until completion of the performance trial)	−0.06 [−1.02, 0.90]
Robson-Ansley et al., 2009 [124]	CS	7/M	24.0	23.1	#	Lab	90 min run of self-paced TT on a motorized treadmill	8% CHO fluid, 8 mL·kg^−1^ 5 min prior to exercise and 2 mL·kg^−1^ every 20 min thereafter	0.44 [−0.63, 1.50]
Robson-Ansley et al., 2011 [125]	CS	9/M	26.0	22.4	58.0	Lab	120 min of continuous running at velocity ~60% $\dot{V}$O_2_max followed by a 5 km TT, TA: 20.0 °C	8% CHO solution immediately before and at 20-min intervals during the preload bout (2 mL·kg^−1^ BM)	0.16 [−0.76, 1.09]
Rollo and Williams 2009 [126]	CS	8/M	31.0	23.1	62.0 *	Lab	60 min performance run, TA: 16 °C	6.4% CHO-E solution (Lucozade Sport, Brentford, UK), 30 min before the 1-hr run (8 mL·kg^−1^ BM) and at 15-min intervals during the run 2 mL·kg^−1^ BM (total: CHO ingestion rate ~60 g·h^−1^)	0.28 [−0.71, 1.26]
Rollo and Williams 2010 [127]	CS	10/M	34.0	23.0	62.0 *	Lab	60 min run as far as possible on an automated treadmill that allowed changes in running speed without manual input, TA: 16.0 °C	6.4% CHO-E solution (8 mL·kg^−1^ BM, Lucozade Sport, Brentford, UK), 30 min before and 2 mL·kg^−1^ BM at 15-min intervals	−0.06 [−0.93, 0.82]
Rollo et al., 2011 [128]	CS	10/M	26.0	22.6	65.0 *	Lab	60 min performance run, TA: 20 °C	6.4% CHO-E solution (Lucozade Sport, Brentford, UK), 30 min before the 1-hr run (8 mL·kg^−1^ BM) and at 15-min intervals during the run 2 mL·kg^−1^ BM (total: CHO ingestion rate ~60 g·h^−1^)	0.40 [−0.49, 1.29]
Smith et al., 2010 i [129]	CS	12/M	31.7	23.4	55.3 *	Lab	120 min of constant-load ride (77% $\dot{V}$O_2_peak) + a 20 km TT, TA: 23.0 °C	1.5% CHO-E solution (GL, ingestion rate 15 g·h^−1^), 2000 mL (250 mL every 15 min, starting at min 15 and ending at min 120)	0.41 [−0.40, 1.22]
Smith et al., 2010 ii [129]	CS	12/M	31.7	23.4	55.3 *	Lab	120 min of constant-load ride (77% $\dot{V}$O_2_peak) + a 20 km TT, TA: 23.0 °C	3.0% CHO-E solution (GL, ingestion rate 30 g·h^−1^), 2000 mL (250 mL every 15 min, starting at min 15 and ending at min 120)	0.49 [−0.32, 1.31]
Smith et al., 2010 iii [129]	CS	12/M	31.7	23.4	55.3 *	Lab	120 min of constant-load ride (77% $\dot{V}$O_2_peak) + a 20 km TT, TA: 23.0 °C	6.0% CHO-E solution (GL, ingestion rate 60 g·h^−1^), 2000 mL (250 mL every 15 min, starting at min 15 and ending at min 120)	0.65 [−0.18, 1.47]
Steiner et al., 2009 [130]	CS	9/6 M 3 F	28.2 M 21.5 F	22.6	45.7 *	Lab	cycling 45 min production trial consisting of a 5-min warm-up at 50 W + by 45 min of self-selected PO at an RPE of 16 (at 70–80 rpm)	6% CHO-E solution (Gatorade Sports Science Institute, Barrington, IL, USA), 240 mL just prior to exercise and every 15 min during the production trial	0.07 [−0.85, 1.00]
Sun et al., 2015 [131]	CS	8/F	28.3	19.8	48.3	Lab	running to exhaustion at 70% $\dot{V}$O_2_max on a treadmill, TA: 20.3–21.3 °C	6% CHO-E solution (3 mL·kg^−1^ BM) every 20 min during exercise	0.62 [−0.39, 1.63]
Tokmakidis & Karamanolis 2008 [132]	CS	11/10 M 1 F	25.3	24.5	49.0	Lab	running at 60% $\dot{V}$O_2_max for 5 min, at 70% for 45 min + at 80% $\dot{V}$O_2_max until exhaustion	~18.5% CHO solution (GL, 1 g·kg^−1^, 400 g), 15 min prior to exercise	0.27 [−0.57, 1.11]
Tsintzas et al., 1993 [133]	CS	7/4 M 3 F	32.6	22.4	61.9	Field	30 km race running	5% CHO solution (1.8% GL-polymer + 2% FRU + 1.2% other CHO: Replay, Bass Ltd.), 250 mL at onset of exercise and 150 mL every 5 km thereafter	0.14 [−0.91, 1.19]
Tsintzas et al., 1995 i [134]	CS	7/M	44.0	21.3	58.4	Lab	marathon-race running, TA: 20.0 °C	6.9% CHO-E drink (3.1% MD + 3.8% SUC), 3 mL·kg^−1^ prior to exercise and 2 mL·kg^−1^ every 5 km thereafter	0.13 [−0.92, 1.17]
Tsintzas et al., 1995 ii [134]	CS	7/M	44.0	21.3	58.4	Lab	marathon-race running, TA: 20.0 °C	5.5% CHO-E drink (2.7% MD + 1.7% GL-syrup + 1.1% FRU-syrup), 3 mL·kg^−1^ prior to exercise and 2 mL·kg^−1^ every 5 km thereafter	0.31 [−0.75, 1.36]
Tsintzas et al., 1996a [135]	CS	8/M	29.7	23.4	61.8	Lab	TE running at 70% $\dot{V}$O_2_max, TA: 19.8 °C	5.5% CHO-E solution (1.7% GL + 1.1% FRU + 0.6% MAL + 2.1% saccharides), 8 mL·kg^−1^ prior to exercise and 2 mL·kg^−1^ every 20 min thereafter	0.89 [−0.16, 1.93]
Tsintzas et al., 1996b i [136]	CS	11/M	27.0	23.3	61.7	Lab	TE running at 70% $\dot{V}$O_2_max, TA: 19.5 °C	5.5% CHO-E solution (1.7% GL + 1.1% FRU + 0.6% MAL + 2.1% saccharides), 8 mL·kg^−1^ prior to exercise and 2 mL·kg^−1^ every 20 min thereafter	0.49 [−0.36, 1.34]
Tsintzas et al., 1996b ii [136]	CS	11/M	27.0	23.3	61.7	Lab	TE running at 70% $\dot{V}$O_2_max, TA: 19.5 °C	6.9% CHO-E drink (Lucozade Sport, SmithKline Beecham, UK), 8 mL·kg^−1^ prior to exercise and 2 mL·kg^−1^ every 20 min thereafter	0.37 [−0.48, 1.21]
Utter et al., 2002 [137]	PS	48/#M #F	41.2−42.7	23.5	49.7	Field	marathon-race running, TA: 19.1 °C	6% CHO-E beverage (Gatorade Sports Science Institute, Barrington, IL, USA), 650 mL ~30 min before the start of the race and 1000 mL every 60 min thereafter	0.04 [−0.36, 0.43]
Van Essen et al., 2006 [138]	CS	10/M	24.0	22.9	63.0 *	Lab	80 km cycling TT, TA: 20.0–23.0 °C	6% CHO-E beverage (SUC), 250 mL every 15 min	0.19 [−0.69, 1.07]
Walton & Rhodes 1997 [139]	CS	10/F	21.9	22.7	45.8	Lab	2 × 19 min periods of high-intensity intermittent running separated by a 10 min break, at 5 min post-exercise + performance trial (repeated 10-sec sprints at 120% $\dot{V}$O_2_max, in a 1:1 work-to-rest ratio, until exhaustion)	12.5% CHO solution, 400 mL at the start of the trial	0.68 [−0.23, 1.59]
Watson et al., 2012 i [140]	CS	12/M	22.0	22.4	54.4	Lab	cycling to volitional exhaustion at 70% $\dot{V}$O_2_max, TA: 10 °C	2% CHO-E solutions (SUC:GL:FRU in a ratio of 50:25:25: Tesco, Ltd., Cheshunt, UK) immediately prior to exercise and every 10 min during exercise, served at 21.0 °C	0.19 [−0.61, 0.99]
Watson et al., 2012 ii [140]	CS	12/M	22.0	22.4	54.4	Lab	cycling to volitional exhaustion at 70% $\dot{V}$O_2_max, TA: 10 °C	4% CHO-E solutions (SUC:GL:FRU in a ratio of 50:25:25: Tesco, Ltd., Cheshunt, UK) immediately prior to exercise and every 10 min during exercise, served at 21.0 °C	0.59 [−0.23, 1.41]
Watson et al., 2012 iii [140]	CS	12/M	22.0	22.4	54.4	Lab	cycling to volitional exhaustion at 70% $\dot{V}$O_2_max, TA: 10 °C	6% CHO-E solutions (SUC:GL:FRU in a ratio of 50:25:25: Tesco, Ltd., Cheshunt, UK) immediately prior to exercise and every 10 min during exercise, served at 21.0 °C	0.60 [−0.22, 1.42]
Watson et al., 2012 iv [140]	CS	12/M	21.0	25.0	52.4	Lab	cycling to volitional exhaustion at 60% $\dot{V}$O_2_max, TA: 30 °C	2% CHO-E solutions (SUC:GL:FRU in a ratio of 50:25:25: Tesco, Ltd., Cheshunt, UK) immediately prior to exercise and every 10 min during exercise, served at 21.0 °C	0.41 [−0.40, 1.22]
Watson et al., 2012 v [140]	CS	12/M	21.0	25.0	52.4	Lab	cycling to volitional exhaustion at 60% $\dot{V}$O_2_max, TA: 30 °C	4% CHO-E solutions (SUC:GL:FRU in a ratio of 50:25:25: Tesco, Ltd., Cheshunt, UK) immediately prior to exercise and every 10 min during exercise, served at 21.0 °C	0.41 [−0.40, 1.22]
Watson et al., 2012 vi [140]	CS	12/M	21.0	25.0	52.4	Lab	cycling to volitional exhaustion at 60% $\dot{V}$O_2_max, TA: 30 °C	6% CHO-E solutions (SUC:GL:FRU in a ratio of 50:25:25: Tesco, Ltd., Cheshunt, UK) immediately prior to exercise and every 10 min during exercise, served at 21.0 °C	0.63 [−0.19, 1.46]
Wilber et al., 1992 [141]	CS	10/M	30.0	20.9	64.9	Lab	TE running at 80% $\dot{V}$O_2_max, TA: 22.0 °C	7% CHO solution (85% GL-polymer + 15% SUC: Exceed, Ross Laboratories, Columbus, OH), 5 min prior to exercise (250 mL) and at 15-min intervals during exercise (150 mL), served at 5.0 °C	0.85 [−0.08, 1.77]
Williams et al., 1990 i [142]	CS	12/M	30.8	21.6	63.4	Lab	30 km-race running, TA: 20.2 °C	5% CHO-E solution (2% GL-polymer + 2% free-GL + 1% other CHO), 5 min prior to exercise 250 mL and every 5 km 150 mL	0.27 [−0.60, 1.14]
Williams et al., 1990 ii [142]	CS	12/M	30.8	21.6	63.4	Lab	30 km-race running, TA: 20.2 °C	5% CHO-E solution (2% GL-polymer + 2% FRU + 1% other CHO), 5 min prior to exercise 250 mL and every 5 km 150 mL	0.18 [−0.68, 1.05]

Total (95%IV) = 0.43 [0.35, 0.51]; Chi² = 172.38, df = 141 (*p* = 0.04); I² = 18%. Test overall effect: Z = 10.31 (*p* < 0.00001). † i–vi denote different intervention arms (trials) within the same study. # Insufficient data. * Data in $\dot{V}$O_2_peak. Abbreviations: BM, body mass; CHO, carbohydrate; CHO-E, carbohydrate enriched with electrolytes; CI, confidence interval; CS, crossover study; DEX, dextrose; E, electrolyte; F, female; FRU, fructose; GAL, galactose; GL, glucose; GL-polymer, glucose polymer; IV, inverse variance; LIST, Loughborough Intermittent Shuttle Test; LT, lactate threshold; Μ, male; MAL, maltose; MAP, maximal aerobic power; MD, maltodextrin; N, sample size; OBLA, onset of blood lactate accumulation; PO, power output; PPO, peak power output; PS, parallel study; RCT, respiratory compensation threshold; SMD, standardized mean difference; SS, steady-state; SSM, soccer simulation match; SUC, sucrose; TA, ambient temperature; TE, time to exhaustion; TF, time to fatigue; TT, time trial; $\dot{V}$O_2_max, maximal oxygen uptake; $\dot{V}$O_2_peak, peak oxygen uptake; Vpeak, peak running velocity; VT, ventilatory threshold; WAT, Wingate anaerobic power test; Wmax, maximal power output.

## Data Availability

All data that support the reported results can be found in the Supplements section.

## Supplementary Materials

The following are available online at https://www.mdpi.com/article/10.3390/nu13124223/s1, Figure S1: Forest plot of comparison, experimental carbohydrate supplementation vs. control on exercise outcome including raw data (in mean and SD) and risk-of-bias judgments of all studies (and trials) for 142 interventions. Abbreviations: *CI*, confidence interval; *IV*, inverse variance; *SD*, standard deviation; *Std*, standardized; *i–vi* denote different intervention arms (trials) within the same study; +, low risk-of-bias; ?, some concerns risk-of-bias; −, high risk-of-bias, Figure S2: Forest plot shows the effects of experimental carbohydrate supplementation as compared to control on exercise outcome for 142 interventions. Subgroup analyses show the results with regard to the subjects’ characteristics in three gender groups (Male, Female, Mixed). The black diamond symbol at the subgroups and at the bottom of the figure represents the standardized mean difference with the 95% confidence intervals for all interventions following random-effect meta-analyses. Studies or trials that provided insufficient data for subgroup classification were not included. Abbreviations: *CI*, confidence interval; *IV*, inverse variance; *SD*, standard deviation; *Std*, standardized; *i–vi* denote different intervention arms (trials) within the same study, Figure S3: Forest plot shows the effects of experimental carbohydrate supplementation as compared to control on exercise outcome for 140 interventions. Subgroup analyses show the results with regard to the subjects’ characteristics in three age groups [Young (18–29 years), Adults I (30–39 years), Adults II (40–49 years)]. The black diamond symbol at the subgroups and at the bottom of the figure represents the standardized mean difference with the 95% confidence intervals for all interventions following random-effect meta-analyses. Studies or trials that provided insufficient data for subgroup classification were not included. Abbreviations: *CI*, confidence interval; *IV*, inverse variance; *SD*, standard deviation; *Std*, standardized; *i–vi* denote different intervention arms (trials) within the same study, Figure S4: Forest plot shows the effects of experimental carbohydrate supplementation as compared to control on exercise outcome for 127 interventions. Subgroup analyses show the results with regards to subjects’ characteristics in four CRF groups (Superior, Excellent, Good and Fair). The black diamond symbol at the subgroups and at the bottom of the figure represents the standardized mean difference with the 95% confidence intervals for all interventions following fixed effects meta-analyses. Studies or trials that provided not sufficient data for subgroup classification were not included. Abbreviations: *CI*, confidence interval; *CRF*, cardio-respiratory fitness; *IV*, inverse variance; *SD*, standard deviation; *Std*, standardized; *i–vi* denote different intervention arms (trials) within the same study, Figure S5: Forest plot shows the effects of experimental carbohydrate supplementation as compared to control on exercise outcome for 142 interventions. Subgroup analyses show the results with regard to exercise task in three exercise mode groups [Cycling, Running, Other (triathlon, duathlon, walking, loaded marching, roller-skiing)]. The black diamond symbol at the subgroups and at the bottom of the figure represents the standardized mean difference with the 95% confidence intervals for all interventions following random-effect meta-analyses. Studies or trials that provided insufficient data for subgroup classification were not included. Abbreviations: *CI*, confidence interval; *IV*, inverse variance; *SD*, standard deviation; *Std*, standardized; *i–vi* denote different intervention arms (trials) within the same study, Figure S6: Forest plot shows the effects of experimental carbohydrate supplementation as compared to control on exercise outcome for 142 interventions. Subgroup analyses show the results with regard to exercise task in two exercise protocol test groups (Capacity, Performance). The black diamond symbol at the subgroups and at the bottom of the figure represents the standardized mean difference with the 95% confidence intervals for all interventions following random-effect meta-analyses. Studies or trials that provided insufficient data for subgroup classification were not included. Abbreviations: *CI*, confidence interval; *IV*, inverse variance; *SD*, standard deviation; *Std*, standardized; *i–vi* denote different intervention arms (trials) within the same study, Figure S7: Forest plot shows the effects of experimental carbohydrate supplementation as compared to control on exercise outcome for 142 interventions. Subgroup analyses show the results with regard to exercise task in three exercise type groups (Intermittent, Continuous, Intermittent shuttle). The black diamond symbol at the subgroups and at the bottom of the figure represents the standardized mean difference with the 95% confidence intervals for all interventions following random-effect meta-analyses. Studies or trials that provided insufficient data for subgroup classification were not included. Abbreviations: *CI*, confidence interval; *IV*, inverse variance; *SD*, standard deviation; *Std*, standardized; *i–vi* denote different intervention arms (trials) within the same study, Figure S8: Forest plot shows the effects of experimental carbohydrate supplementation as compared to control on exercise outcome for 142 interventions. Subgroup analyses show the results with regard to supplementation in seven carbohydrate concentration groups (0% < CHO ≤ 2%, 2% < CHO ≤ 4%, 4% < CHO ≤ 6%, 6% < CHO ≤ 8%, 8% < CHO ≤ 10%, 10% < CHO ≤ 15%, 15% < CHO ≤ 20%). The black diamond symbol at the subgroups and at the bottom of the figure represents the standardized mean difference with the 95% confidence intervals for all interventions following random-effect meta-analyses. Studies or trials that provided insufficient data for subgroup classification were not included. Abbreviations: *CHO*, carbohydrate; *CI*, confidence interval; *IV*, inverse variance; *SD*, standard deviation; *Std*, standardized; *i–vi* denote different intervention arms (trials) within the same study, Figure S9: Forest plot shows the effects of experimental carbohydrate supplementation as compared to control on exercise outcome for 31 interventions. Subgroup analyses show the results with regard to supplementation in five carbohydrate dose groups (CHO dose ≤ 40 g·hr−1, 40 g·hr−1 < CHO dose ≤ 60 g·hr−1, 60 g·hr−1 < CHO dose ≤ 80 g·hr−1, 80 g·hr−1 < CHO dose ≤ 100 g·hr−1, CHO dose > 100 g·hr−1). The black diamond symbol at the subgroups and at the bottom of the figure represents the standardized mean difference with the 95% confidence intervals for all interventions following random-effect meta-analyses. Studies or trials that provided insufficient data for subgroup classification were not included. Abbreviations: *CHO*, carbohydrate; *CI*, confidence interval; *IV*, inverse variance; *SD*, standard deviation; *Std*, standardized; *i–vi* denote different intervention arms (trials) within the same study, Figure S10: Forest plot shows the effects of experimental carbohydrate supplementation as compared to control on exercise outcome for 53 interventions. Subgroup analyses show the results with regard to supplementation in six single source carbohydrate only groups (GL, MD, SUC, MAL, FRU, GAL). The black diamond symbol at the subgroups and at the bottom of the figure represents the standardized mean difference with the 95% confidence intervals for all interventions following random-effect meta-analyses. Studies or trials that provided insufficient data for subgroup classification were not included. Abbreviations: *CHO*, carbohydrate; *CI*, confidence interval; *FRU*, fructose; *GAL*, galactose; *GL*, glucose; *IV*, inverse variance; *MD*, maltodextrin; *MAL*, maltose; *SD*, standard deviation; *Std*, standardized; *SUC*, sucrose; *i–vi* denote different intervention arms (trials) within the same study, Figure S11: Forest plot shows the effects of experimental carbohydrate supplementation as compared to control on exercise outcome for 103 interventions. Subgroup analyses show the results with regard to supplementation in three carbohydrate solution formulation groups (Single-source CHO solution, Double-source CHO solution, Triple-or-more-source CHO solution). The black diamond symbol at the subgroups and at the bottom of the figure represents the standardized mean difference with the 95% confidence intervals for all interventions following random-effect meta-analyses. Studies or trials that provided insufficient data for subgroup classification were not included. Abbreviations: *CHO*, carbohydrate; *CI*, confidence interval; *IV*, inverse variance; *SD*, standard deviation; *Std*, standardized; *i–vi* denote different intervention arms (trials) within the same study, Figure S12: Forest plot shows the effects of experimental carbohydrate supplementation as compared to control on exercise outcome for 142 interventions. Subgroup analyses show the results with regard to supplementation in four carbohydrate administration time groups (Prior to or at the beginning, During, Prior to or at the beginning + during, Late in exercise). The black diamond symbol at the subgroups and at the bottom of the figure represents the standardized mean difference with the 95% confidence intervals for all interventions following random-effect meta-analyses. Studies or trials that provided insufficient data for subgroup classification were not included. Abbreviations: *CHO*, carbohydrate; *CI*, confidence interval; *IV*, inverse variance; *SD*, standard deviation; *Std*, standardized; *i–vi* denote different intervention arms (trials) within the same study, Figure S13: Forest plot shows the effects of experimental carbohydrate supplementation as compared to control on exercise outcome for 21 interventions. Subgroup analyses show the results with regard to supplementation in two temperature of supplement administration groups [Cool (< 18 °C), Neutral (18–26 °C)]. The black diamond symbol at the subgroups and at the bottom of the figure represents the standardized mean difference with the 95% confidence intervals for all interventions following random-effect meta-analyses. Studies or trials that provided insufficient data for subgroup classification were not included. Abbreviations: *CHO*, carbohydrate; *CI*, confidence interval; *IV*, inverse variance; *SD*, standard deviation; *Std*, standardized; *i–vi* denote different intervention arms (trials) within the same study, Figure S14: Forest plot shows the effects of experimental carbohydrate supplementation as compared to control on exercise outcome for 50 interventions. Subgroup analyses show the results with regards to supplementation in twelve MTC groups (GL:FRU, GL:SUC, GL:MD, MD:FRU, MD:DEX, MD:SUC, GL:MD:FRU, GL:MD:DEX, GL:SUC:FRU, GL:MD:MAL:Saccharides, SUC:MD:IsoMAL, Unclear CHO substances mixture). The black diamond symbol at the subgroups and at the bottom of the figure represents the standardized mean difference with the 95% confidence intervals for all interventions following fixed effects meta-analyses. Studies or trials that provided not sufficient data for subgroup classification were not included. Abbreviations: *CHO*, carbohydrate, *CI*, confi-dence interval; *DEX*, dextrose; *FRU*, fructose; *GAL*, galactose; *GL*, glucose; *IV*, inverse variance; *MD*, maltodextrin; *MAL*, maltose; *MTC*, multiple transportable carbohydrate; *SD*, standard deviation; *Std*, standardized; *SUC*, sucrose; *i–vi* denote different intervention arms (trials) within the same study, Figure S15: Forest plot shows the effects of experimental carbohydrate supplementation as compared to control on exercise outcome for 102 interventions. Subgroup analyses show the results with regard to ambient conditions in two thermal condition groups [Cool (<18 °C), Neutral (18–26 °C), Heat (>26 °C)]. The black diamond symbol at the subgroups and at the bottom of the figure represents the standardized mean difference with the 95% confidence intervals for all interventions following random-effect meta-analyses. Studies or trials that provided insufficient data for subgroup classification were not included. Abbreviations: *CHO*, carbohydrate; *CI*, confidence interval; *IV*, inverse variance; *SD*, standard deviation; *Std*, standardized; *i–vi* denote different intervention arms (trials) within the same study, Figure S16: Funnel plot of comparison: I. in all studies (and trials); and II.–VII. in classes of different subgroups analysis.
