# Supplementary material for: Meta-Analysis of Carbohydrate Solution Intake during Prolonged Exercise in Adults: From the Last 45+ Years’ Perspective
Source: Nutrients. 2021 Nov 24;13(12):4223. doi: 10.3390/nu13124223 (PMC8704222; doi:10.3390/nu13124223)

Study or Subgroup	Experimental Mean	SD	Total	Control Mean	SD	Total	Weight	Std. Mean Difference IV, Random, 95% CI
Acker-Hewitt et al. 2012 [47]	-43.8	4.1	10	-44.2	4.5	10	0.7%	0.09 [-0.79, 0.97]
Alghannam 2011 [48]	16.49	3.25	6	11	2.8	6	0.3%	1.67 [0.28, 3.07]
Ali et al. 2007 [49]	-2.5	0.13	16	-2.53	0.13	16	1.0%	0.22 [-0.47, 0.92]
Anastasiou et al. 2004 i [50]	-67.2	2	10	-69.4	3	10	0.7%	0.83 [-0.10, 1.75]
Anastasiou et al. 2004 ii [50]	-68.6	1.7	10	-69.4	3	10	0.7%	0.31 [-0.57, 1.20]
Anastasiou et al. 2004 iii [50]	-66.7	2	10	-69.4	3	10	0.6%	1.01 [0.07, 1.96]
Angus et al. 2000 [51]	-166	7	8	-178	11	8	0.5%	1.23 [0.13, 2.33]
Bailey et al. 2000 i [52]	188	44	9	166	28	9	0.6%	0.57 [-0.38, 1.52]
Bailey et al. 2000 ii [52]	180	44	9	165	40	9	0.6%	0.34 [-0.59, 1.27]
Baldassarre et al. 2021 [53]	169	91.06	8	102.31	57.47	8	0.5%	0.83 [-0.21, 1.86]
Baur et al. 2014 i [54]	-50.4	2.2	8	-52.9	3.7	8	0.5%	0.78 [-0.25, 1.80]
Baur et al. 2014 ii [54]	-51.1	2.4	8	-52.9	3.7	8	0.6%	0.55 [-0.46, 1.55]
Baur et al. 2014 iii [54]	-52	3.7	8	-52.9	3.7	8	0.6%	0.23 [-0.75, 1.21]
Below et al. 1995 [55]	-10.23	0.79	8	-10.92	0.9	8	0.5%	0.77 [-0.26, 1.80]
Bishop et al. 2001 [56]	98	21	9	77	24	9	0.6%	0.89 [-0.09, 1.87]
Burgess et al. 1991 [57]	963.9	1,350.6	9	745.9	860	9	0.6%	0.18 [-0.74, 1.11]
Byrne et al. 2005 [58]	146	52	14	134	52	14	0.9%	0.22 [-0.52, 0.97]
Campbell et al. 2008 [59]	-17.3	2.4	16	-17.8	2.6	16	1.0%	0.19 [-0.50, 0.89]
Carter et al. 2003 i [60]	145.6	31.09	7	123.1	29.3	7	0.5%	0.70 [-0.39, 1.79]
Carter et al. 2003 ii [60]	60.6	26.65	8	50.8	21.91	8	0.6%	0.38 [-0.61, 1.37]
Carter et al. 2005 i [61]	152	51.76	8	128	39.88	8	0.6%	0.49 [-0.51, 1.49]
Carter et al. 2005 ii [61]	145.1	34.22	8	128	39.88	8	0.6%	0.44 [-0.56, 1.43]
Clark et al. 2000 [62]	-59	4	7	-59	4	7	0.5%	0.00 [-1.05, 1.05]
Clarke et al. 2011 [63]	72.1	5	12	57.1	5	12	0.4%	2.90 [1.70, 4.10]
Cole et al. 1993 i [64]	190.92	16.5	10	186.4	20.36	10	0.7%	0.23 [-0.65, 1.11]
Cole et al. 1993 ii [64]	190.48	15.36	10	186.4	20.36	10	0.7%	0.22 [-0.66, 1.10]
Cole et al. 1993 iii [64]	190.32	15.06	10	186.4	20.36	10	0.7%	0.21 [-0.67, 1.09]
Cole et al. 2018 [65]	-0.7	0.21	14	-1.78	0.31	14	0.3%	3.96 [2.62, 5.30]
Coletta et al. 2013 i [66]	-8.2	1.2	12	-8.3	1.2	12	0.8%	0.08 [-0.72, 0.88]
Coletta et al. 2013 ii [66]	-8.4	1.5	12	-8.3	1.2	12	0.8%	-0.07 [-0.87, 0.73]
Cureton et al. 2007 [67]	190	36	16	178	31	16	1.0%	0.35 [-0.35, 1.05]
Currell et al. 2008 i [68]	254	8	8	231	9	8	0.3%	2.55 [1.14, 3.97]
Currell et al. 2008 ii [68]	275	10	8	231	9	8	0.2%	4.37 [2.37, 6.37]
Davis et al. 2000 [69]	11.2	5.9	8	8.5	4.5	8	0.6%	0.49 [-0.51, 1.49]
Desbrow et al. 2004 [70]	-62.57	6.73	9	-62.73	5.58	9	0.7%	0.02 [-0.90, 0.95]
Duhamel et al. 2007 [71]	137	27.1	15	115	23.2	15	0.9%	0.85 [0.10, 1.60]
El-Sayed et al. 1995 [72]	5.58	0.53	9	5.12	0.95	9	0.6%	0.57 [-0.38, 1.52]
El-Sayed et al. 1997 [73]	41.5	3	8	41	3	8	0.6%	0.16 [-0.82, 1.14]
Finger et al. 2018 [74]	-1,270.3	130.5	13	-1,275.4	120	13	0.9%	0.04 [-0.73, 0.81]
Fulco et al. 2007 i [75]	-101	24	9	-116	24.5	6	0.5%	0.58 [-0.48, 1.64]
Fulco et al. 2007 ii [75]	-95	24	9	-107	29.4	6	0.5%	0.43 [-0.62, 1.48]
Funnell et al. 2017 i [76]	2.07	0.06	16	2.09	0.08	16	1.0%	-0.28 [-0.97, 0.42]
Funnell et al. 2017 ii [76]	2.04	0.09	16	2.06	0.08	16	1.0%	-0.23 [-0.92, 0.47]
Ganio et al. 2010 [77]	225	39	14	205	52	14	0.9%	0.42 [-0.33, 1.17]
Glace et al. 2019 i [78]	-5.55	1.39	10	-5.98	1.55	10	0.7%	0.28 [-0.60, 1.16]
Glace et al. 2019 ii [78]	-6.93	1.39	10	-7.51	1.55	10	0.7%	0.38 [-0.51, 1.26]
Glace et al. 2019 iii [78]	12.2	15.8	10	7.3	7.9	10	0.7%	0.38 [-0.51, 1.26]
Glace et al. 2019 iv [78]	18.6	10	10	18.8	11.7	10	0.7%	-0.02 [-0.89, 0.86]
Goedecke et al. 2013 [79]	381	267	22	294	159	22	1.3%	0.39 [-0.21, 0.99]
Gui et al. 2017 [80]	-129.6	8.8	11	-134.6	11.5	11	0.7%	0.47 [-0.38, 1.32]
Heesch et al. 2014 i [81]	-17.7	1.4708	8	-18.13	1.470782	8	0.6%	0.28 [-0.71, 1.26]
Heesch et al. 2014 ii [81]	-17.85	1.6405	8	-18.13	1.470782	8	0.6%	0.17 [-0.81, 1.15]
Heesch et al. 2014 iii [81]	-17.6	1.7536	8	-18.13	1.470782	8	0.6%	0.31 [-0.68, 1.30]
Hulston & Jeukendrup 2008 [82]	-45.45	3.38	10	-47.4	4.11	10	0.7%	0.50 [-0.40, 1.39]
Hulston & Jeukendrup 2009 [83]	-59.69	2.87	10	-65.94	5.56	10	0.6%	1.35 [0.36, 2.35]
Ivy et al. 1983 [84]	299	31	10	268.3	37.3	10	0.6%	0.86 [-0.07, 1.78]
Jarvis et al. 1999 [85]	431.7	63.3	10	425.7	72.7	10	0.7%	0.08 [-0.79, 0.96]
Jeukendrup et al. 1997 [86]	-58.74	2.27	19	-60.15	2.83	19	1.1%	0.54 [-0.11, 1.19]
Kang et al. 1995 [87]	189	60.8	7	154	45	7	0.5%	0.61 [-0.47, 1.69]
Kang et al. 1996 [88]	187	47.6	7	164	42.3	7	0.5%	0.48 [-0.59, 1.55]
Khanna & Manna 2005 [89]	94.1	17.7	10	62.3	10.4	10	0.5%	2.10 [0.96, 3.24]
King et al. 2018 i [90]	206	41	10	187	43	10	0.7%	0.43 [-0.46, 1.32]
King et al. 2018 ii [90]	196	46	10	187	43	10	0.7%	0.19 [-0.69, 1.07]
King et al. 2018 iii [90]	225	45	10	187	43	10	0.7%	0.83 [-0.10, 1.75]
King et al. 2018 iv [90]	213	43	10	187	43	10	0.7%	0.58 [-0.32, 1.48]
King et al. 2019 i [91]	219	32	11	186	37	11	0.7%	0.92 [0.03, 1.81]
King et al. 2019 ii [91]	228	37	11	186	37	11	0.7%	1.09 [0.18, 2.00]
King et al. 2019 iii [91]	212	48	11	186	37	11	0.7%	0.58 [-0.27, 1.44]
Kingwell et al. 1989 [92]	36.1	4.9	9	33.8	3.2	9	0.6%	0.53 [-0.42, 1.47]
Learsi et al. 2019 i [93]	-18.5	0.9	9	-20.2	2.4	9	0.6%	0.89 [-0.09, 1.87]
Learsi et al. 2019 ii [93]	-18.7	0.4	9	-21.2	1.4	9	0.4%	2.31 [1.05, 3.57]
Lugo et al. 1993 [94]	-33.45	7.65	9	-49.77	23.82	9	0.6%	0.88 [-0.10, 1.86]
Martínez-Lagunas et al. 2010 [95]	26.9	21.131	12	14.7	11.77795	12	0.8%	0.69 [-0.14, 1.52]
Maughan et al. 1989 [96]	90.8	30.4	6	76.2	22.3	6	0.4%	0.51 [-0.65, 1.66]
McConell et al. 1996 [97]	268	22.6	8	242	25.5	8	0.5%	1.02 [-0.04, 2.08]
McConell et al. 1999 [98]	199	59.4	8	152	25.5	8	0.5%	0.97 [-0.08, 2.03]
McConell et al. 2000 [99]	68.1	14.8	13	69.6	19.8	13	0.9%	-0.08 [-0.85, 0.69]
McGawley et al. 2012 [100]	-38.72	3.6999	10	-40.37	4.110961	10	0.7%	0.40 [-0.48, 1.29]
Millard-Stafford et al. 1990 [101]	-141.9	8.8	10	-143.1	7.5	10	0.7%	0.14 [-0.74, 1.02]
Millard-Stafford et al. 2005 i [102]	-1,078	102	10	-1,154	177	10	0.7%	0.50 [-0.39, 1.40]
Millard-Stafford et al. 2005 ii [102]	-1,062	98	10	-1,154	177	10	0.7%	0.62 [-0.29, 1.52]
Millard-Stafford et al. 2007 [103]	79	14.1	16	74.5	11.7	16	1.0%	0.34 [-0.36, 1.04]
Morris et al. 2003 [104]	7,099	1,941	9	7,786	2,223	9	0.6%	-0.31 [-1.24, 0.62]
Murray et al. 1987 i [105]	-401	52	13	-432	43	13	0.8%	0.63 [-0.16, 1.42]
Murray et al. 1987 ii [105]	-384	39	13	-432	43	13	0.8%	1.13 [0.29, 1.97]
Murray et al. 1987 iii [105]	-375	30	13	-432	43	13	0.7%	1.49 [0.60, 2.37]
Murray et al. 1989 i [106]	-13.03	0.83	12	-13.62	1.14	12	0.8%	0.57 [-0.25, 1.39]
Murray et al. 1989 ii [106]	-13.3	0.87	12	-13.62	1.14	12	0.8%	0.30 [-0.50, 1.11]
Murray et al. 1989 iii [106]	-13.57	0.76	12	-13.62	1.14	12	0.8%	0.05 [-0.75, 0.85]
Murray et al. 1991 i [107]	-476	27.8	10	-505	59.1	10	0.7%	0.60 [-0.30, 1.50]
Murray et al. 1991 ii [107]	-483.8	40.2	10	-505	59.1	10	0.7%	0.40 [-0.49, 1.29]
Murray et al. 1991 iii [107]	-474.3	60.4	10	-505	59.1	10	0.7%	0.49 [-0.40, 1.38]
Naclerio et al. 2014 [108]	-107.58	7.59	10	-107.5	8.41	10	0.7%	-0.01 [-0.89, 0.87]
Nassif et al. 2014 [109]	-140.5	18.1	10	-130.2	11.2	10	0.7%	-0.66 [-1.56, 0.25]
Nassis et al. 1998 [110]	110.2	21.4	9	112.5	23.3	9	0.7%	-0.10 [-1.02, 0.83]
Newell et al. 2015 i [111]	-35.3	4.27	20	-37.03	5.58	20	1.2%	0.34 [-0.28, 0.97]
Newell et al. 2015 ii [111]	-34.32	3.12	20	-37.03	5.58	20	1.2%	0.59 [-0.05, 1.22]
Newell et al. 2015 iii [111]	-34.18	3.13	20	-37.03	5.58	20	1.2%	0.62 [-0.02, 1.25]
Nicholas et al. 1995 [112]	8.9	4.5	9	6.7	3	9	0.6%	0.55 [-0.40, 1.49]
Nikolopoulos et al. 2004 [113]	58.97	24.89	8	51.3	16.89	8	0.6%	0.34 [-0.65, 1.33]
Nishibata et al. 1993 [114]	92.05	29.55	7	98.07	27.33	7	0.5%	-0.20 [-1.25, 0.85]
Oosthuyse et al. 2015a i [115]	-28.45	2.68	9	-28.65	2.82	9	0.7%	0.07 [-0.86, 0.99]
Oosthuyse et al. 2015a ii [115]	-29.65	3.28	9	-28.65	2.82	9	0.6%	-0.31 [-1.24, 0.62]
Oosthuyse et al. 2015b [116]	-28.2	2.88	8	-28.68	3.04	8	0.6%	0.15 [-0.83, 1.14]
Osterberg et al. 2008 [117]	-37.1	3.8	13	-39.7	4.6	13	0.8%	0.60 [-0.19, 1.39]
O’Hara et al. 2014 i [118]	68.7	10.2	10	63.9	16.2	10	0.7%	0.34 [-0.54, 1.22]
O’Hara et al. 2014 ii [118]	58.5	24.9	10	63.9	16.2	10	0.7%	-0.25 [-1.13, 0.63]
O’Neal et al. 2013 [119]	423	82	36	414	92	36	1.7%	0.10 [-0.36, 0.56]
Pettersson et al. 2019 [120]	-239	16	12	-238	16	12	0.8%	-0.06 [-0.86, 0.74]
Pottier et al. 2010 [121]	-63.6	5.5	12	-62.6	6.6	12	0.8%	-0.16 [-0.96, 0.64]
Rilley et al. 1988 [122]	106	24	9	102	24	9	0.6%	0.16 [-0.77, 1.08]
Roberts et al. 2014 i [123]	-5,722.8	695.9	14	-6,117.5	876.9	6	0.6%	0.50 [-0.47, 1.48]
Roberts et al. 2014 ii [123]	-6,165	695.9	14	-6,117.5	876.9	6	0.6%	-0.06 [-1.02, 0.90]
Robson-Ansley et al. 2009 [124]	19.13	1.7	7	18.29	1.9	7	0.5%	0.44 [-0.63, 1.50]
Robson-Ansley et al. 2011 [125]	-143	12.9	9	-145	10.2	9	0.6%	0.16 [-0.76, 1.09]
Rollo & Williams 2009 [126]	14,046	1,104	8	13,715	1,143	8	0.6%	0.28 [-0.71, 1.26]
Rollo & Williams 2010 [127]	13,589	1,635	10	13,680	1,525	10	0.7%	-0.06 [-0.93, 0.82]
Rollo et al. 2011 [128]	14,515	756	10	14,190	800	10	0.7%	0.40 [-0.49, 1.29]
Smith et al. 2010 i [129]	-35.2	2.8	12	-36.4	2.9	12	0.8%	0.41 [-0.40, 1.22]
Smith et al. 2010 ii [129]	-35	2.6	12	-36.4	2.9	12	0.8%	0.49 [-0.32, 1.31]
Smith et al. 2010 iii [129]	-34.7	2.1	12	-36.4	2.9	12	0.8%	0.65 [-0.18, 1.47]
Steiner et al. 2009 [130]	241.67	25.74	9	240	18.37	9	0.7%	0.07 [-0.85, 1.00]
Sun et al. 2015 [131]	106.2	26.59	8	91.6	16.69	8	0.6%	0.62 [-0.39, 1.63]
Tokmakidis & Karamanolis 2008 [132]	40.1	4.3	11	38.8	5	11	0.8%	0.27 [-0.57, 1.11]
Tsintzas et al. 1993 [133]	-128.3	19.9	7	-131.2	18.7	7	0.5%	0.14 [-0.91, 1.19]
Tsintzas et al. 1995 i [134]	-192.4	8.7	7	-193.9	13.2	7	0.5%	0.13 [-0.92, 1.17]
Tsintzas et al. 1995 ii [134]	-190	10.3	7	-193.9	13.2	7	0.5%	0.31 [-0.75, 1.36]
Tsintzas et al. 1996a [135]	132.4	34.8	8	104.3	24.3	8	0.5%	0.89 [-0.16, 1.93]
Tsintzas et al. 1996b i [136]	124.5	26.6	11	109.6	31.8	11	0.7%	0.49 [-0.36, 1.34]
Tsintzas et al. 1996b ii [136]	121.4	29.7	11	109.6	31.8	11	0.8%	0.37 [-0.48, 1.21]
Utter et al. 2002 [137]	-258	252	48	-268.2	294	50	2.1%	0.04 [-0.36, 0.43]
Van Essen et al. 2006 [138]	-135	28.5	10	-141	31.6	10	0.7%	0.19 [-0.69, 1.07]
Walton & Rhodes 1997 [139]	648	358	10	436	223	10	0.7%	0.68 [-0.23, 1.59]
Watson et al. 2012 i [140]	109.2	33.9	12	102.6	33.9	12	0.8%	0.19 [-0.61, 0.99]
Watson et al. 2012 ii [140]	121	25.7	12	102.6	33.9	12	0.8%	0.59 [-0.23, 1.41]
Watson et al. 2012 iii [140]	122.4	29.9	12	102.6	33.9	12	0.8%	0.60 [-0.22, 1.42]
Watson et al. 2012 iv [140]	104.1	20.1	12	94.5	24.5	12	0.8%	0.41 [-0.40, 1.22]
Watson et al. 2012 v [140]	105.5	26.7	12	94.5	24.5	12	0.8%	0.41 [-0.40, 1.22]
Watson et al. 2012 vi [140]	112	28.7	12	94.5	24.5	12	0.8%	0.63 [-0.19, 1.46]
Wilber et al. 1992 [141]	115	25	10	92	27	10	0.7%	0.85 [-0.08, 1.77]
Williams et al. 1990 i [142]	-124.8	14.9	12	-129.3	17.7	9	0.7%	0.27 [-0.60, 1.14]
Williams et al. 1990 ii [142]	-125.9	17.9	12	-129.3	17.7	9	0.7%	0.18 [-0.68, 1.05]
**Total (95% CI)**			**1560**			**1534**	**100.0%**	**0.43 [0.35, 0.51]**

Heterogeneity: $Tau^2 = 0.04$; $Chi^2 = 172.38$, df = 141 (P = 0.04); $I^2 = 18\%$

Test for overall effect: Z = 10.31 (P < 0.00001)

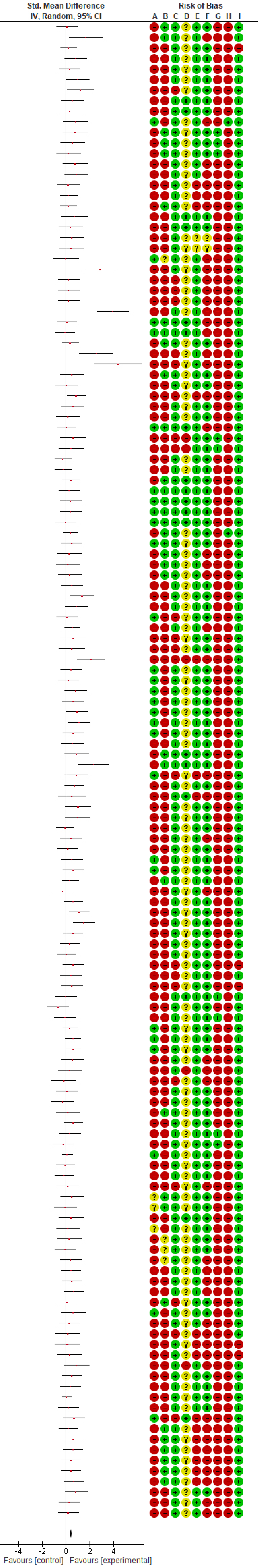

Risk of bias legend
(**A**) Bias arising from the subjects' inclusion process
(**B**) Bias arising from the statistical power calculation process
(**C**) Bias arising from the subjects' familiarization process
(**D**) Bias arising from the generation of the random interventions (trials) allocation sequence
(**E**) Bias arising from the blind subjects' to their treatment allocation
(**F**) Bias arising from the blind researchers' to treatment allocations
(**G**) Bias due to lack of mentioning or pure reliability of measures
(**H**) Bias due to lack of mentioning or pure validity of measures
(**I**) Bias due to the subjects' complication or dropout ≥ 15%

Figure S1: Forest plot of comparison, experimental carbohydrate supplementation vs control on exercise outcome including raw data (in mean and SD) and risk-of-bias judgments of all studies (and trials) for 142 interventions. Abbreviations: CI, confidence interval; IV, inverse variance; SD, standard deviation; Std, standardized; i-vi denote different intervention arms (trials) within the same study; +, low risk-of-bias; ?, some concerns risk-of-bias; –, high risk-of-bias

Study or Subgroup	Weight	Std. Mean Difference IV, Random, 95% CI
**Gender of the subjects: Male**		
Acker-Hewitt et al. 2012 [47]	0.7%	0.09 [-0.79, 0.97]
Alghannam 2011 [48]	0.3%	1.67 [0.28, 3.07]
Ali et al. 2007 [49]	1.0%	0.22 [-0.47, 0.92]
Anastasiou et al. 2004 i [50]	0.7%	0.83 [-0.10, 1.75]
Anastasiou et al. 2004 ii [50]	0.7%	0.31 [-0.57, 1.20]
Anastasiou et al. 2004 iii [50]	0.6%	1.01 [0.07, 1.96]
Angus et al. 2000 [51]	0.5%	1.23 [0.13, 2.33]
Baur et al. 2014 i [54]	0.5%	0.78 [-0.25, 1.80]
Baur et al. 2014 ii [54]	0.6%	0.55 [-0.46, 1.55]
Baur et al. 2014 iii [54]	0.6%	0.23 [-0.75, 1.21]
Below et al. 1995 [55]	0.5%	0.77 [-0.26, 1.80]
Bishop et al. 2001 [56]	0.6%	0.89 [-0.09, 1.87]
Burgess et al. 1991 [57]	0.7%	0.18 [-0.74, 1.11]
Byrne et al. 2005 [58]	0.9%	0.22 [-0.52, 0.97]
Carter et al. 2003 i [60]	0.5%	0.70 [-0.39, 1.79]
Carter et al. 2003 ii [60]	0.6%	0.38 [-0.61, 1.37]
Carter et al. 2005 i [61]	0.6%	0.49 [-0.51, 1.49]
Carter et al. 2005 ii [61]	0.6%	0.44 [-0.56, 1.43]
Clarke et al. 2011 [63]	0.4%	2.90 [1.70, 4.10]
Cole et al. 1993 i [64]	0.7%	0.23 [-0.65, 1.11]
Cole et al. 1993 ii [64]	0.7%	0.22 [-0.66, 1.10]
Cole et al. 1993 iii [64]	0.7%	0.21 [-0.67, 1.09]
Cole et al. 2018 [65]	0.3%	3.96 [2.62, 5.30]
Coletta et al. 2013 i [66]	0.8%	0.08 [-0.72, 0.88]
Coletta et al. 2013 ii [66]	0.8%	-0.07 [-0.87, 0.73]
Cureton et al. 2007 [67]	1.0%	0.35 [-0.35, 1.05]
Currell et al. 2008 i [68]	0.3%	2.55 [1.14, 3.97]
Currell et al. 2008 ii [68]	0.2%	4.37 [2.37, 6.37]
Davis et al. 2000 [69]	0.6%	0.49 [-0.51, 1.49]
Desbrow et al. 2004 [70]	0.7%	0.02 [-0.90, 0.95]
El-Sayed et al. 1995 [72]	0.6%	0.57 [-0.38, 1.52]
El-Sayed et al. 1997 [73]	0.6%	0.16 [-0.82, 1.14]
Finger et al. 2018 [74]	0.9%	0.04 [-0.73, 0.81]
Funnell et al. 2017 i [76]	1.0%	-0.28 [-0.97, 0.42]
Funnell et al. 2017 ii [76]	1.0%	-0.23 [-0.92, 0.47]
Ganio et al. 2010 [77]	0.9%	0.42 [-0.33, 1.17]
Glace et al. 2019 i [78]	0.7%	0.28 [-0.60, 1.16]
Glace et al. 2019 iii [78]	0.7%	0.38 [-0.51, 1.26]
Goedecke et al. 2013 [79]	1.3%	0.39 [-0.21, 0.99]
Heesch et al. 2014 i [81]	0.6%	0.28 [-0.71, 1.26]
Heesch et al. 2014 ii [81]	0.6%	0.17 [-0.81, 1.15]
Heesch et al. 2014 iii [81]	0.6%	0.31 [-0.68, 1.30]
Hulston & Jeukendrup 2008 [82]	0.7%	0.50 [-0.40, 1.39]
Hulston & Jeukendrup 2009 [83]	0.6%	1.35 [0.36, 2.35]
Ivy et al. 1983 [84]	0.7%	0.86 [-0.07, 1.78]
Kang et al. 1995 [87]	0.5%	0.61 [-0.47, 1.69]
Kang et al. 1996 [88]	0.5%	0.48 [-0.59, 1.55]
Khanna & Manna 2005 [89]	0.5%	2.10 [0.96, 3.24]
King et al. 2018 i [90]	0.7%	0.43 [-0.46, 1.32]
King et al. 2018 ii [90]	0.7%	0.19 [-0.69, 1.07]
King et al. 2018 iii [90]	0.7%	0.83 [-0.10, 1.75]
King et al. 2018 iv [90]	0.7%	0.58 [-0.32, 1.48]
King et al. 2019 i [91]	0.7%	0.92 [0.03, 1.81]
King et al. 2019 ii [91]	0.7%	1.09 [0.18, 2.00]
King et al. 2019 iii [91]	0.7%	0.58 [-0.27, 1.44]
Kingwell et al. 1989 [92]	0.6%	0.53 [-0.42, 1.47]
Learsi et al. 2019 i [93]	0.6%	0.89 [-0.09, 1.87]
Learsi et al. 2019 ii [93]	0.4%	2.31 [1.05, 3.57]
Lugo et al. 1993 [94]	0.6%	0.88 [-0.10, 1.86]
Maughan et al. 1989 [96]	0.4%	0.51 [-0.65, 1.66]
McConell et al. 1996 [97]	0.5%	1.02 [-0.04, 2.08]
McConell et al. 1999 [98]	0.5%	0.97 [-0.08, 2.03]
McConell et al. 2000 [99]	0.9%	-0.08 [-0.85, 0.69]
Millard-Stafford et al. 1990 [101]	0.7%	0.14 [-0.74, 1.02]
Millard-Stafford et al. 2005 i [102]	0.7%	0.50 [-0.39, 1.40]
Millard-Stafford et al. 2005 ii [102]	0.7%	0.62 [-0.29, 1.52]
Millard-Stafford et al. 2007 [103]	1.0%	0.34 [-0.36, 1.04]
Morris et al. 2003 [104]	0.6%	-0.31 [-1.24, 0.62]
Murray et al. 1987 i [105]	0.8%	0.63 [-0.16, 1.42]
Murray et al. 1987 ii [105]	0.8%	1.13 [0.29, 1.97]
Murray et al. 1987 iii [105]	0.7%	1.49 [0.60, 2.37]
Naclerio et al. 2014 [108]	0.7%	-0.01 [-0.89, 0.87]
Nassif et al. 2014 [109]	0.7%	-0.66 [-1.56, 0.25]
Newell et al. 2015 i [111]	1.2%	0.34 [-0.28, 0.97]
Newell et al. 2015 ii [111]	1.2%	0.59 [-0.05, 1.22]
Newell et al. 2015 iii [111]	1.2%	0.62 [-0.02, 1.25]
Nicholas et al. 1995 [112]	0.6%	0.55 [-0.40, 1.49]
Nikolopoulos et al. 2004 [113]	0.6%	0.34 [-0.65, 1.33]
Nishibata et al. 1993 [114]	0.5%	-0.20 [-1.25, 0.85]
Oosthuyse et al. 2015a i [115]	0.7%	0.07 [-0.86, 0.99]
Oosthuyse et al. 2015a ii [115]	0.6%	-0.31 [-1.24, 0.62]
Oosthuyse et al. 2015b [116]	0.6%	0.15 [-0.83, 1.14]
Osterberg et al. 2008 [117]	0.8%	0.60 [-0.19, 1.39]
O'Hara et al. 2014 i [118]	0.7%	0.34 [-0.54, 1.22]
O'Hara et al. 2014 ii [118]	0.7%	-0.25 [-1.13, 0.63]
Rilley et al. 1988 [122]	0.7%	0.16 [-0.77, 1.08]
Roberts et al. 2014 i [123]	0.6%	0.50 [-0.47, 1.48]
Roberts et al. 2014 ii [123]	0.6%	-0.06 [-1.02, 0.90]
Robson-Ansley et al. 2009 [124]	0.5%	0.44 [-0.63, 1.50]
Robson-Ansley et al. 2011 [125]	0.7%	0.16 [-0.76, 1.09]
Rollo & Williams 2009 [126]	0.6%	0.28 [-0.71, 1.26]
Rollo & Williams 2010 [127]	0.7%	-0.06 [-0.93, 0.82]
Rollo et al. 2011 [128]	0.7%	0.40 [-0.49, 1.29]
Smith et al. 2010 i [129]	0.8%	0.41 [-0.40, 1.22]
Smith et al. 2010 ii [129]	0.8%	0.49 [-0.32, 1.31]
Smith et al. 2010 iii [129]	0.8%	0.65 [-0.18, 1.47]
Tsintzas et al. 1995 i [134]	0.5%	0.13 [-0.92, 1.17]
Tsintzas et al. 1995 ii [134]	0.5%	0.31 [-0.75, 1.36]
Tsintzas et al. 1996a [135]	0.5%	0.89 [-0.16, 1.93]
Tsintzas et al. 1996b i [136]	0.8%	0.49 [-0.36, 1.34]
Tsintzas et al. 1996b ii [136]	0.8%	0.37 [-0.48, 1.21]
Van Essen et al. 2006 [138]	0.7%	0.19 [-0.69, 1.07]
Watson et al. 2012 i [140]	0.8%	0.19 [-0.61, 0.99]
Watson et al. 2012 ii [140]	0.8%	0.59 [-0.23, 1.41]
Watson et al. 2012 iii [140]	0.8%	0.60 [-0.22, 1.42]
Watson et al. 2012 iv [140]	0.8%	0.41 [-0.40, 1.22]
Watson et al. 2012 v [140]	0.8%	0.41 [-0.40, 1.22]
Watson et al. 2012 vi [140]	0.8%	0.63 [-0.19, 1.46]
Wilber et al. 1992 [141]	0.7%	0.85 [-0.08, 1.77]
Williams et al. 1990 i [142]	0.7%	0.27 [-0.60, 1.14]
Williams et al. 1990 ii [142]	0.7%	0.18 [-0.68, 1.05]
**Subtotal (95% CI)**	**76.1%**	**0.48 [0.38, 0.58]**
Heterogeneity: Tau² = 0.08; Chi² = 153.86, df = 110 (P = 0.004); I² = 29%		
Test for overall effect: Z = 9.23 (P < 0.00001)		
**Gender of the subjects: Female**		
Bailey et al. 2000 i [52]	0.6%	0.57 [-0.38, 1.52]
Bailey et al. 2000 ii [52]	0.6%	0.34 [-0.59, 1.27]
Glace et al. 2019 ii [78]	0.7%	0.38 [-0.51, 1.26]
Glace et al. 2019 iv [78]	0.7%	-0.02 [-0.89, 0.86]
Gui et al. 2017 [80]	0.8%	0.47 [-0.38, 1.32]
Jarvis et al. 1999 [85]	0.7%	0.08 [-0.79, 0.96]
Sun et al. 2015 [131]	0.6%	0.62 [-0.39, 1.63]
Walton & Rhodes 1997 [139]	0.7%	0.68 [-0.23, 1.59]
**Subtotal (95% CI)**	**5.4%**	**0.38 [0.06, 0.70]**
Heterogeneity: Tau² = 0.00; Chi² = 2.07, df = 7 (P = 0.96); I² = 0%		
Test for overall effect: Z = 2.31 (P = 0.02)		
**Gender of the subjects: Mixed**		
Baldassarre et al. 2021 [53]	0.5%	0.83 [-0.21, 1.86]
Campbell et al. 2008 [59]	1.0%	0.19 [-0.50, 0.89]
Clark et al. 2000 [62]	0.5%	0.00 [-1.05, 1.05]
Duhamel et al. 2007 [71]	0.9%	0.85 [0.10, 1.60]
Fulco et al. 2007 i [75]	0.5%	0.58 [-0.48, 1.64]
Fulco et al. 2007 ii [75]	0.5%	0.43 [-0.62, 1.48]
Jeukendrup et al. 1997 [86]	1.1%	0.54 [-0.11, 1.19]
Martínez-Lagunas et al. 2010 [95]	0.8%	0.69 [-0.14, 1.52]
McGawley et al. 2012 [100]	0.7%	0.40 [-0.48, 1.29]
Murray et al. 1989 i [106]	0.8%	0.57 [-0.25, 1.39]
Murray et al. 1989 ii [106]	0.8%	0.30 [-0.50, 1.11]
Murray et al. 1989 iii [106]	0.8%	0.05 [-0.75, 0.85]
Murray et al. 1991 i [107]	0.7%	0.60 [-0.30, 1.50]
Murray et al. 1991 ii [107]	0.7%	0.40 [-0.49, 1.29]
Murray et al. 1991 iii [107]	0.7%	0.49 [-0.40, 1.38]
Nassis et al. 1998 [110]	0.7%	-0.10 [-1.02, 0.83]
O'Neal et al. 2013 [119]	1.8%	0.10 [-0.36, 0.56]
Pettersson et al. 2019 [120]	0.8%	-0.06 [-0.86, 0.74]
Steiner et al. 2009 [130]	0.7%	0.07 [-0.85, 1.00]
Tokmakidis & Karamanolis 2008 [132]	0.8%	0.27 [-0.57, 1.11]
Tsintzas et al. 1993 [133]	0.5%	0.14 [-0.91, 1.19]
Utter et al. 2002 [137]	2.1%	0.04 [-0.36, 0.43]
**Subtotal (95% CI)**	**18.5%**	**0.28 [0.12, 0.44]**
Heterogeneity: Tau² = 0.00; Chi² = 10.82, df = 21 (P = 0.97); I² = 0%		
Test for overall effect: Z = 3.41 (P = 0.0007)		
**Total (95% CI)**	**100.0%**	**0.43 [0.35, 0.52]**
Heterogeneity: Tau² = 0.04; Chi² = 170.41, df = 140 (P = 0.04); I² = 18%		
Test for overall effect: Z = 10.40 (P < 0.00001)		
Test for subgroup differences: Chi² = 4.10, df = 2 (P = 0.13), I² = 51.3%		

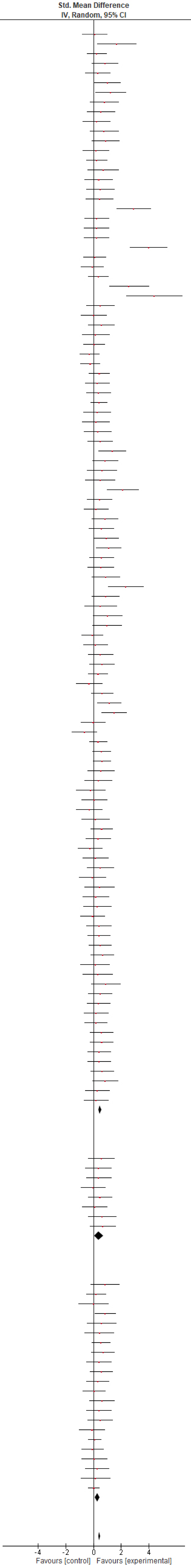

Figure S2: Forest plot shows the effects of experimental carbohydrate supplementation as compared to control on exercise outcome for 142 interventions. Subgroup analyses show the results with regard to the subjects' characteristics in three gender groups (Male, Female, Mixed). The black diamond symbol at the subgroups and at the bottom of the figure represents the standardized mean difference with the 95% confidence intervals for all interventions following random-effect meta-analyses. Studies or trials that provided insufficient data for subgroup classification were not included. Abbreviations: CI, confidence interval; IV, inverse variance; SD, standard deviation; Std, standardized; i-vi denote different intervention arms (trials) within the same study

Study or Subgroup	Weight	Std. Mean Difference IV, Random, 95% CI
**Age of the subjects: Young (18–29 years)**		
Acker-Hewitt et al. 2012 [47]	0.7%	0.09 [-0.79, 0.97]
Alghannam 2011 [48]	0.3%	1.67 [0.28, 3.07]
Ali et al. 2007 [49]	1.0%	0.22 [-0.47, 0.92]
Anastasiou et al. 2004 i [50]	0.7%	0.83 [-0.10, 1.75]
Anastasiou et al. 2004 ii [50]	0.7%	0.31 [-0.57, 1.20]
Anastasiou et al. 2004 iii [50]	0.6%	1.01 [0.07, 1.96]
Angus et al. 2000 [51]	0.5%	1.23 [0.13, 2.33]
Bailey et al. 2000 i [52]	0.6%	0.57 [-0.38, 1.52]
Bailey et al. 2000 ii [52]	0.7%	0.34 [-0.59, 1.27]
Baldassarre et al. 2021 [53]	0.5%	0.83 [-0.21, 1.86]
Baur et al. 2014 i [54]	0.6%	0.78 [-0.25, 1.80]
Baur et al. 2014 ii [54]	0.6%	0.55 [-0.46, 1.55]
Baur et al. 2014 iii [54]	0.6%	0.23 [-0.75, 1.21]
Below et al. 1995 [55]	0.6%	0.77 [-0.26, 1.80]
Bishop et al. 2001 [56]	0.6%	0.89 [-0.09, 1.87]
Burgess et al. 1991 [57]	0.7%	0.18 [-0.74, 1.11]
Byrne et al. 2005 [58]	0.9%	0.22 [-0.52, 0.97]
Carter et al. 2003 i [60]	0.5%	0.70 [-0.39, 1.79]
Carter et al. 2003 ii [60]	0.6%	0.38 [-0.61, 1.37]
Carter et al. 2005 i [61]	0.6%	0.49 [-0.51, 1.49]
Carter et al. 2005 ii [61]	0.6%	0.44 [-0.56, 1.43]
Clark et al. 2000 [62]	0.5%	0.00 [-1.05, 1.05]
Clarke et al. 2011 [63]	0.4%	2.90 [1.70, 4.10]
Cole et al. 1993 i [64]	0.7%	0.23 [-0.65, 1.11]
Cole et al. 1993 ii [64]	0.7%	0.22 [-0.66, 1.10]
Cole et al. 1993 iii [64]	0.7%	0.21 [-0.67, 1.09]
Cureton et al. 2007 [67]	1.0%	0.35 [-0.35, 1.05]
Davis et al. 2000 [69]	0.6%	0.49 [-0.51, 1.49]
Duhamel et al. 2007 [71]	0.9%	0.85 [0.10, 1.60]
El-Sayed et al. 1995 [72]	0.6%	0.57 [-0.38, 1.52]
El-Sayed et al. 1997 [73]	0.6%	0.16 [-0.82, 1.14]
Finger et al. 2018 [74]	0.9%	0.04 [-0.73, 0.81]
Funnell et al. 2017 i [76]	1.0%	-0.28 [-0.97, 0.42]
Funnell et al. 2017 ii [76]	1.0%	-0.23 [-0.92, 0.47]
Ganio et al. 2010 [77]	0.9%	0.42 [-0.33, 1.17]
Goedecke et al. 2013 [79]	1.3%	0.39 [-0.21, 0.99]
Hulston & Jeukendrup 2008 [82]	0.7%	0.50 [-0.40, 1.39]
Hulston & Jeukendrup 2009 [83]	0.6%	1.35 [0.36, 2.35]
Ivy et al. 1983 [84]	0.7%	0.86 [-0.07, 1.78]
Jeukendrup et al. 1997 [86]	1.1%	0.54 [-0.11, 1.19]
Kang et al. 1995 [87]	0.5%	0.61 [-0.47, 1.69]
Kang et al. 1996 [88]	0.5%	0.48 [-0.59, 1.55]
Khanna & Manna 2005 [89]	0.5%	2.10 [0.96, 3.24]
Kingwell et al. 1989 [92]	0.6%	0.53 [-0.42, 1.47]
Learsi et al. 2019 i [93]	0.6%	0.89 [-0.09, 1.87]
Learsi et al. 2019 ii [93]	0.4%	2.31 [1.05, 3.57]
Lugo et al. 1993 [94]	0.6%	0.88 [-0.10, 1.86]
Martínez-Lagunas et al. 2010 [95]	0.8%	0.69 [-0.14, 1.52]
Maughan et al. 1989 [96]	0.4%	0.51 [-0.65, 1.66]
McConell et al. 1996 [97]	0.5%	1.02 [-0.04, 2.08]
McConell et al. 1999 [98]	0.5%	0.97 [-0.08, 2.03]
McConell et al. 2000 [99]	0.9%	-0.08 [-0.85, 0.69]
McGawley et al. 2012 [100]	0.7%	0.40 [-0.48, 1.29]
Millard-Stafford et al. 1990 [101]	0.7%	0.14 [-0.74, 1.02]
Millard-Stafford et al. 2005 i [102]	0.7%	0.50 [-0.39, 1.40]
Millard-Stafford et al. 2005 ii [102]	0.7%	0.62 [-0.29, 1.52]
Millard-Stafford et al. 2007 [103]	1.0%	0.34 [-0.36, 1.04]
Morris et al. 2003 [104]	0.7%	-0.31 [-1.24, 0.62]
Naclerio et al. 2014 [108]	0.7%	-0.01 [-0.89, 0.87]
Nassif et al. 2014 [109]	0.7%	-0.66 [-1.56, 0.25]
Nassis et al. 1998 [110]	0.7%	-0.10 [-1.02, 0.83]
Nicholas et al. 1995 [112]	0.6%	0.55 [-0.40, 1.49]
Nikolopoulos et al. 2004 [113]	0.6%	0.34 [-0.65, 1.33]
Nishibata et al. 1993 [114]	0.5%	-0.20 [-1.25, 0.85]
O'Neal et al. 2013 [119]	1.8%	0.10 [-0.36, 0.56]
Pettersson et al. 2019 [120]	0.8%	-0.06 [-0.86, 0.74]
Robson-Ansley et al. 2009 [124]	0.5%	0.44 [-0.63, 1.50]
Robson-Ansley et al. 2011 [125]	0.7%	0.16 [-0.76, 1.09]
Rollo et al. 2011 [128]	0.7%	0.40 [-0.49, 1.29]
Steiner et al. 2009 [130]	0.7%	0.07 [-0.85, 1.00]
Sun et al. 2015 [131]	0.6%	0.62 [-0.39, 1.63]
Tokmakidis & Karamanolis 2008 [132]	0.8%	0.27 [-0.57, 1.11]
Tsintzas et al. 1996a [135]	0.5%	0.89 [-0.16, 1.93]
Tsintzas et al. 1996b i [136]	0.8%	0.49 [-0.36, 1.34]
Tsintzas et al. 1996b ii [136]	0.8%	0.37 [-0.48, 1.21]
Van Essen et al. 2006 [138]	0.7%	0.19 [-0.69, 1.07]
Walton & Rhodes 1997 [139]	0.7%	0.68 [-0.23, 1.59]
Watson et al. 2012 i [140]	0.8%	0.19 [-0.61, 0.99]
Watson et al. 2012 ii [140]	0.8%	0.59 [-0.23, 1.41]
Watson et al. 2012 iii [140]	0.8%	0.60 [-0.22, 1.42]
Watson et al. 2012 iv [140]	0.8%	0.41 [-0.40, 1.22]
Watson et al. 2012 v [140]	0.8%	0.41 [-0.40, 1.22]
Watson et al. 2012 vi [140]	0.8%	0.63 [-0.19, 1.46]
**Subtotal (95% CI)**	**58.4%**	**0.43 [0.33, 0.53]**
Heterogeneity: $Tau^2 = 0.01$; $Chi^2 = 85.54$, df = 82 (P = 0.37); $I^2 = 4\%$		
Test for overall effect: Z = 8.49 (P < 0.00001)		
**Age of the subjects: Adults I (30–39 years)**		
Campbell et al. 2008 [59]	1.0%	0.19 [-0.50, 0.89]
Currell et al. 2008 i [68]	0.3%	2.55 [1.14, 3.97]
Currell et al. 2008 ii [68]	0.2%	4.37 [2.37, 6.37]
Desbrow et al. 2004 [70]	0.7%	0.02 [-0.90, 0.95]
Fulco et al. 2007 i [75]	0.5%	0.58 [-0.48, 1.64]
Fulco et al. 2007 ii [75]	0.5%	0.43 [-0.62, 1.48]
Glace et al. 2019 i [78]	0.7%	0.28 [-0.60, 1.16]
Glace et al. 2019 iii [78]	0.7%	0.38 [-0.51, 1.26]
Gui et al. 2017 [80]	0.8%	0.47 [-0.38, 1.32]
Heesch et al. 2014 i [81]	0.6%	0.28 [-0.71, 1.26]
Heesch et al. 2014 ii [81]	0.6%	0.17 [-0.81, 1.15]
Heesch et al. 2014 iii [81]	0.6%	0.31 [-0.68, 1.30]
Jarvis et al. 1999 [85]	0.7%	0.08 [-0.79, 0.96]
King et al. 2018 i [90]	0.7%	0.43 [-0.46, 1.32]
King et al. 2018 ii [90]	0.7%	0.19 [-0.69, 1.07]
King et al. 2018 iii [90]	0.7%	0.83 [-0.10, 1.75]
King et al. 2018 iv [90]	0.7%	0.58 [-0.32, 1.48]
King et al. 2019 i [91]	0.7%	0.92 [0.03, 1.81]
King et al. 2019 ii [91]	0.7%	1.09 [0.18, 2.00]
King et al. 2019 iii [91]	0.7%	0.58 [-0.27, 1.44]
Murray et al. 1987 i [105]	0.9%	0.63 [-0.16, 1.42]
Murray et al. 1987 ii [105]	0.8%	1.13 [0.29, 1.97]
Murray et al. 1987 iii [105]	0.7%	1.49 [0.60, 2.37]
Murray et al. 1989 i [106]	0.8%	0.57 [-0.25, 1.39]
Murray et al. 1989 ii [106]	0.8%	0.30 [-0.50, 1.11]
Murray et al. 1989 iii [106]	0.8%	0.05 [-0.75, 0.85]
Murray et al. 1991 i [107]	0.7%	0.60 [-0.30, 1.50]
Murray et al. 1991 ii [107]	0.7%	0.40 [-0.49, 1.29]
Murray et al. 1991 iii [107]	0.7%	0.49 [-0.40, 1.38]
Newell et al. 2015 i [111]	1.2%	0.34 [-0.28, 0.97]
Newell et al. 2015 ii [111]	1.2%	0.59 [-0.05, 1.22]
Newell et al. 2015 iii [111]	1.2%	0.62 [-0.02, 1.25]
Oosthuyse et al. 2015a i [115]	0.7%	0.07 [-0.86, 0.99]
Oosthuyse et al. 2015a ii [115]	0.7%	-0.31 [-1.24, 0.62]
Oosthuyse et al. 2015b [116]	0.6%	0.15 [-0.83, 1.14]
Osterberg et al. 2008 [117]	0.9%	0.60 [-0.19, 1.39]
O'Hara et al. 2014 i [118]	0.7%	0.34 [-0.54, 1.22]
O'Hara et al. 2014 ii [118]	0.7%	-0.25 [-1.13, 0.63]
Pottier et al. 2010 [121]	0.8%	-0.16 [-0.96, 0.64]
Rilley et al. 1988 [122]	0.7%	0.16 [-0.77, 1.08]
Roberts et al. 2014 i [123]	0.6%	0.50 [-0.47, 1.48]
Roberts et al. 2014 ii [123]	0.6%	-0.06 [-1.02, 0.90]
Rollo & Williams 2009 [126]	0.6%	0.28 [-0.71, 1.26]
Rollo & Williams 2010 [127]	0.7%	-0.06 [-0.93, 0.82]
Smith et al. 2010 i [129]	0.8%	0.41 [-0.40, 1.22]
Smith et al. 2010 ii [129]	0.8%	0.49 [-0.32, 1.31]
Smith et al. 2010 iii [129]	0.8%	0.65 [-0.18, 1.47]
Tsintzas et al. 1993 [133]	0.5%	0.14 [-0.91, 1.19]
Wilber et al. 1992 [141]	0.7%	0.85 [-0.08, 1.77]
Williams et al. 1990 i [142]	0.7%	0.27 [-0.60, 1.14]
Williams et al. 1990 ii [142]	0.7%	0.18 [-0.68, 1.05]
**Subtotal (95% CI)**	**36.7%**	**0.43 [0.31, 0.56]**
Heterogeneity: $Tau^2 = 0.01$; $Chi^2 = 53.04$, df = 50 (P = 0.36); $I^2 = 6\%$		
Test for overall effect: Z = 6.76 (P < 0.00001)		
**Age of the subjects: Adults II (40–49 years)**		
Cole et al. 2018 [65]	0.3%	3.96 [2.62, 5.30]
Glace et al. 2019 ii [78]	0.7%	0.38 [-0.51, 1.26]
Glace et al. 2019 iv [78]	0.7%	-0.02 [-0.89, 0.86]
Tsintzas et al. 1995 i [134]	0.5%	0.13 [-0.92, 1.17]
Tsintzas et al. 1995 ii [134]	0.5%	0.31 [-0.75, 1.36]
Utter et al. 2002 [137]	2.1%	0.04 [-0.36, 0.43]
**Subtotal (95% CI)**	**4.9%**	**0.69 [-0.18, 1.55]**
Heterogeneity: $Tau^2 = 0.93$; $Chi^2 = 30.91$, df = 5 (P < 0.00001); $I^2 = 84\%$		
Test for overall effect: Z = 1.56 (P = 0.12)		
**Total (95% CI)**	**100.0%**	**0.44 [0.35, 0.52]**
Heterogeneity: $Tau^2 = 0.04$; $Chi^2 = 170.28$, df = 139 (P = 0.04); $I^2 = 18\%$		
Test for overall effect: Z = 10.39 (P < 0.00001)		
Test for subgroup differences: $Chi^2 = 0.34$, df = 2 (P = 0.84), $I^2 = 0\%$		

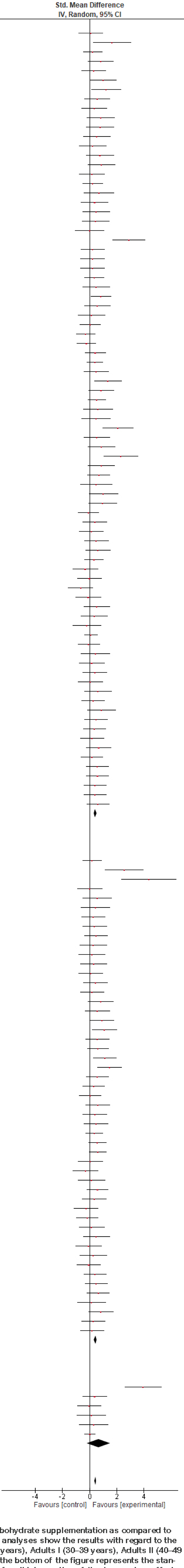

Figure S3: Forest plot shows the effects of experimental carbohydrate supplementation as compared to control on exercise outcome for 140 interventions. Subgroup analyses show the results with regard to the subjects' characteristics in three age groups [Young (18–29 years), Adults I (30–39 years), Adults II (40–49 years)]. The black diamond symbol at the subgroups and at the bottom of the figure represents the standardized mean difference with the 95% confidence intervals for all interventions following random-effect meta-analyses. Studies or trials that provided insufficient data for subgroup classification were not included. Abbreviations: CI, confidence interval; IV, inverse variance; SD, standard deviation; Std, standardized; i-vi denote different intervention arms (trials) within the same study

Study or Subgroup	Weight	Std. Mean Difference IV, Fixed, 95% CI
**CRF of the subjects: Superior**		
Acker-Hewitt et al. 2012 [47]	0.8%	0.09 [-0.79, 0.97]
Ali et al. 2007 [49]	1.2%	0.22 [-0.47, 0.92]
Anastasiou et al. 2004 i [50]	0.7%	0.83 [-0.10, 1.75]
Anastasiou et al. 2004 ii [50]	0.8%	0.31 [-0.57, 1.20]
Anastasiou et al. 2004 iii [50]	0.7%	1.01 [0.07, 1.96]
Angus et al. 2000 [51]	0.5%	1.23 [0.13, 2.33]
Bailey et al. 2000 i [52]	0.7%	0.57 [-0.38, 1.52]
Bailey et al. 2000 ii [52]	0.7%	0.34 [-0.59, 1.27]
Baur et al. 2014 i [54]	0.6%	0.78 [-0.25, 1.80]
Baur et al. 2014 ii [54]	0.6%	0.55 [-0.46, 1.55]
Baur et al. 2014 iii [54]	0.6%	0.23 [-0.75, 1.21]
Below et al. 1995 [55]	0.6%	0.77 [-0.26, 1.80]
Burgess et al. 1991 [57]	0.7%	0.18 [-0.74, 1.11]
Campbell et al. 2008 [59]	1.2%	0.19 [-0.50, 0.89]
Carter et al. 2003 i [60]	0.5%	0.70 [-0.39, 1.79]
Carter et al. 2003 ii [60]	0.6%	0.38 [-0.61, 1.37]
Carter et al. 2005 i [61]	0.6%	0.49 [-0.51, 1.49]
Carter et al. 2005 ii [61]	0.6%	0.44 [-0.56, 1.43]
Clark et al. 2000 [62]	0.5%	0.00 [-1.05, 1.05]
Clarke et al. 2011 [63]	0.4%	2.90 [1.70, 4.10]
Cole et al. 1993 i [64]	0.8%	0.23 [-0.65, 1.11]
Cole et al. 1993 ii [64]	0.8%	0.22 [-0.66, 1.10]
Cole et al. 1993 iii [64]	0.8%	0.21 [-0.67, 1.09]
Cole et al. 2018 [65]	0.3%	3.96 [2.62, 5.30]
Cureton et al. 2007 [67]	1.2%	0.35 [-0.35, 1.05]
Currell et al. 2008 i [68]	0.3%	2.55 [1.14, 3.97]
Currell et al. 2008 ii [68]	0.1%	4.37 [2.37, 6.37]
Desbrow et al. 2004 [70]	0.7%	0.02 [-0.90, 0.95]
El-Sayed et al. 1995 [72]	0.7%	0.57 [-0.38, 1.52]
El-Sayed et al. 1997 [73]	0.6%	0.16 [-0.82, 1.14]
Finger et al. 2018 [74]	1.0%	0.04 [-0.73, 0.81]
Ganio et al. 2010 [77]	1.1%	0.42 [-0.33, 1.17]
Glace et al. 2019 i [78]	0.8%	0.28 [-0.60, 1.16]
Glace et al. 2019 ii [78]	0.8%	0.38 [-0.51, 1.26]
Glace et al. 2019 iii [78]	0.8%	0.38 [-0.51, 1.26]
Glace et al. 2019 iv [78]	0.8%	-0.02 [-0.89, 0.86]
Gui et al. 2017 [80]	0.8%	0.47 [-0.38, 1.32]
Heesch et al. 2014 i [81]	0.6%	0.28 [-0.71, 1.26]
Heesch et al. 2014 ii [81]	0.6%	0.17 [-0.81, 1.15]
Heesch et al. 2014 iii [81]	0.6%	0.31 [-0.68, 1.30]
Hulston & Jeukendrup 2008 [82]	0.8%	0.50 [-0.40, 1.39]
Hulston & Jeukendrup 2009 [83]	0.6%	1.35 [0.36, 2.35]
Ivy et al. 1983 [84]	0.7%	0.86 [-0.07, 1.78]
Jarvis et al. 1999 [85]	0.8%	0.08 [-0.79, 0.96]
Jeukendrup et al. 1997 [86]	1.4%	0.54 [-0.11, 1.19]
Kang et al. 1995 [87]	0.5%	0.61 [-0.47, 1.69]
Kang et al. 1996 [88]	0.5%	0.48 [-0.59, 1.55]
King et al. 2018 i [90]	0.8%	0.43 [-0.46, 1.32]
King et al. 2018 ii [90]	0.8%	0.19 [-0.69, 1.07]
King et al. 2018 iii [90]	0.7%	0.83 [-0.10, 1.75]
King et al. 2018 iv [90]	0.7%	0.58 [-0.32, 1.48]
King et al. 2019 i [91]	0.8%	0.92 [0.03, 1.81]
King et al. 2019 ii [91]	0.7%	1.09 [0.18, 2.00]
King et al. 2019 iii [91]	0.8%	0.58 [-0.27, 1.44]
Kingwell et al. 1989 [92]	0.7%	0.53 [-0.42, 1.47]
Lugo et al. 1993 [94]	0.6%	0.88 [-0.10, 1.86]
Martínez-Lagunas et al. 2010 [95]	0.9%	0.69 [-0.14, 1.52]
McConell et al. 1996 [97]	0.5%	1.02 [-0.04, 2.08]
McConell et al. 1999 [98]	0.5%	0.97 [-0.08, 2.03]
McConell et al. 2000 [99]	1.0%	-0.08 [-0.85, 0.69]
McGawley et al. 2012 [100]	0.8%	0.40 [-0.48, 1.29]
Millard-Stafford et al. 1990 [101]	0.8%	0.14 [-0.74, 1.02]
Millard-Stafford et al. 2005 i [102]	0.8%	0.50 [-0.39, 1.40]
Millard-Stafford et al. 2005 ii [102]	0.7%	0.62 [-0.29, 1.52]
Millard-Stafford et al. 2007 [103]	1.2%	0.34 [-0.36, 1.04]
Morris et al. 2003 [104]	0.7%	-0.31 [-1.24, 0.62]
Nassif et al. 2014 [109]	0.7%	-0.66 [-1.56, 0.25]
Nassis et al. 1998 [110]	0.7%	-0.10 [-1.02, 0.83]
Newell et al. 2015 i [111]	1.5%	0.34 [-0.28, 0.97]
Newell et al. 2015 ii [111]	1.5%	0.59 [-0.05, 1.22]
Newell et al. 2015 iii [111]	1.5%	0.62 [-0.02, 1.25]
Nicholas et al. 1995 [112]	0.7%	0.55 [-0.40, 1.49]
Nikolopoulos et al. 2004 [113]	0.6%	0.34 [-0.65, 1.33]
Oosthuyse et al. 2015a i [115]	0.7%	0.07 [-0.86, 0.99]
Oosthuyse et al. 2015a ii [115]	0.7%	-0.31 [-1.24, 0.62]
Oosthuyse et al. 2015b [116]	0.6%	0.15 [-0.83, 1.14]
Osterberg et al. 2008 [117]	1.0%	0.60 [-0.19, 1.39]
O'Hara et al. 2014 i [118]	0.8%	0.34 [-0.54, 1.22]
O'Hara et al. 2014 ii [118]	0.8%	-0.25 [-1.13, 0.63]
Pettersson et al. 2019 [120]	0.9%	-0.06 [-0.86, 0.74]
Pottier et al. 2010 [121]	0.9%	-0.16 [-0.96, 0.64]
Rilley et al. 1988 [122]	0.7%	0.16 [-0.77, 1.08]
Roberts et al. 2014 i [123]	0.6%	0.50 [-0.47, 1.48]
Roberts et al. 2014 ii [123]	0.7%	-0.06 [-1.02, 0.90]
Robson-Ansley et al. 2011 [125]	0.7%	0.16 [-0.76, 1.09]
Rollo & Williams 2009 [126]	0.6%	0.28 [-0.71, 1.26]
Rollo & Williams 2010 [127]	0.8%	-0.06 [-0.93, 0.82]
Rollo et al. 2011 [128]	0.8%	0.40 [-0.49, 1.29]
Smith et al. 2010 i [129]	0.9%	0.41 [-0.40, 1.22]
Smith et al. 2010 ii [129]	0.9%	0.49 [-0.32, 1.31]
Smith et al. 2010 iii [129]	0.9%	0.65 [-0.18, 1.47]
Tsintzas et al. 1993 [133]	0.5%	0.14 [-0.91, 1.19]
Tsintzas et al. 1995 i [134]	0.5%	0.13 [-0.92, 1.17]
Tsintzas et al. 1995 ii [134]	0.5%	0.31 [-0.75, 1.36]
Tsintzas et al. 1996a [135]	0.6%	0.89 [-0.16, 1.93]
Tsintzas et al. 1996b i [136]	0.8%	0.49 [-0.36, 1.34]
Tsintzas et al. 1996b ii [136]	0.8%	0.37 [-0.48, 1.21]
Utter et al. 2002 [137]	3.8%	0.04 [-0.36, 0.43]
Van Essen et al. 2006 [138]	0.8%	0.19 [-0.69, 1.07]
Wilber et al. 1992 [141]	0.7%	0.85 [-0.08, 1.77]
Williams et al. 1990 i [142]	0.8%	0.27 [-0.60, 1.14]
Williams et al. 1990 ii [142]	0.8%	0.18 [-0.68, 1.05]
**Subtotal (95% CI)**	**79.5%**	**0.40 [0.31, 0.49]**
Heterogeneity: Chi² = 122.78, df = 101 (P = 0.07); I² = 18%		
Test for overall effect: Z = 9.04 (P < 0.00001)		
**CRF of the subjects: Excellent**		
Alghannam 2011 [48]	0.3%	1.67 [0.28, 3.07]
Bishop et al. 2001 [56]	0.6%	0.89 [-0.09, 1.87]
Byrne et al. 2005 [58]	1.1%	0.22 [-0.52, 0.97]
Davis et al. 2000 [69]	0.6%	0.49 [-0.51, 1.49]
Funnell et al. 2017 i [76]	1.2%	-0.28 [-0.97, 0.42]
Funnell et al. 2017 ii [76]	1.2%	-0.23 [-0.92, 0.47]
Goedecke et al. 2013 [79]	1.7%	0.39 [-0.21, 0.99]
Khanna & Manna 2005 [89]	0.5%	2.10 [0.96, 3.24]
Maughan et al. 1989 [96]	0.4%	0.51 [-0.65, 1.66]
Sun et al. 2015 [131]	0.6%	0.62 [-0.39, 1.63]
Walton & Rhodes 1997 [139]	0.7%	0.68 [-0.23, 1.59]
Watson et al. 2012 i [140]	0.9%	0.19 [-0.61, 0.99]
Watson et al. 2012 ii [140]	0.9%	0.59 [-0.23, 1.41]
Watson et al. 2012 iii [140]	0.9%	0.60 [-0.22, 1.42]
Watson et al. 2012 iv [140]	0.9%	0.41 [-0.40, 1.22]
Watson et al. 2012 v [140]	0.9%	0.41 [-0.40, 1.22]
Watson et al. 2012 vi [140]	0.9%	0.63 [-0.19, 1.46]
**Subtotal (95% CI)**	**14.4%**	**0.43 [0.23, 0.64]**
Heterogeneity: Chi² = 21.19, df = 16 (P = 0.17); I² = 24%		
Test for overall effect: Z = 4.16 (P < 0.0001)		
**CRF of the subjects: Good**		
Murray et al. 1987 i [105]	1.0%	0.63 [-0.16, 1.42]
Murray et al. 1987 ii [105]	0.9%	1.13 [0.29, 1.97]
Murray et al. 1987 iii [105]	0.8%	1.49 [0.60, 2.37]
Nishibata et al. 1993 [114]	0.5%	-0.20 [-1.25, 0.85]
Tokmakidis & Karamanolis 2008 [132]	0.9%	0.27 [-0.57, 1.11]
**Subtotal (95% CI)**	**4.0%**	**0.71 [0.32, 1.10]**
Heterogeneity: Chi² = 7.92, df = 4 (P = 0.09); I² = 49%		
Test for overall effect: Z = 3.59 (P = 0.0003)		
**CRF of the subjects: Fair**		
Duhamel et al. 2007 [71]	1.1%	0.85 [0.10, 1.60]
Learsi et al. 2019 i [93]	0.6%	0.89 [-0.09, 1.87]
Learsi et al. 2019 ii [93]	0.4%	2.31 [1.05, 3.57]
**Subtotal (95% CI)**	**2.1%**	**1.13 [0.59, 1.67]**
Heterogeneity: Chi² = 4.15, df = 2 (P = 0.13); I² = 52%		
Test for overall effect: Z = 4.11 (P < 0.0001)		
**Total (95% CI)**	**100.0%**	**0.43 [0.36, 0.51]**
Heterogeneity: Chi² = 164.99, df = 126 (P = 0.01); I² = 24%		
Test for overall effect: Z = 10.95 (P < 0.00001)		
Test for subgroup differences: Chi² = 8.96, df = 3 (P = 0.03), I² = 66.5%		

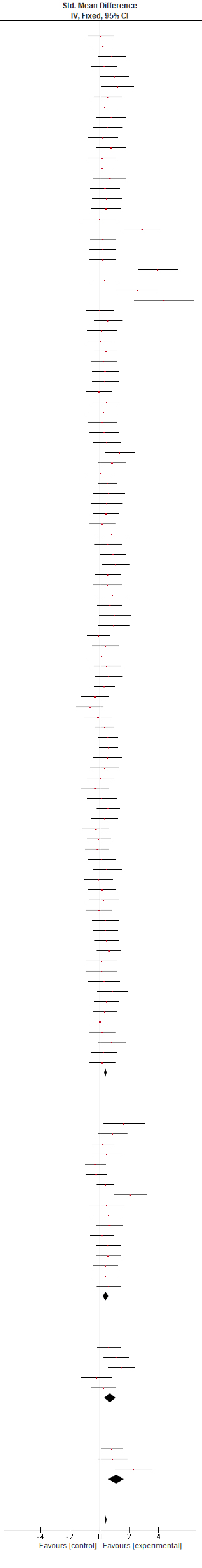

Figure S4: Forest plot shows the effects of experimental carbohydrate supplementation as compared to control on exercise outcome for 127 interventions. Subgroup analyses show the results with regards to subjects' characteristics in four CRF groups (Superior, Excellent, Good and Fair). The black diamond symbol at the subgroups and at the bottom of the figure represents the standardized mean difference with the 95% confidence intervals for all interventions following fixed effects meta-analyses. Studies or trials that provided not sufficient data for subgroup classification were not included. Abbreviations: CI, confidence interval; CRF, cardio-respiratory fitness; IV, inverse variance; SD, standard deviation; Std, standardized; i-vi denote different intervention arms (trials) within the same study

Study or Subgroup	Weight	Std. Mean Difference IV, Random, 95% CI
**Exercise mode: Cycling**		
Acker-Hewitt et al. 2012 [47]	0.7%	0.09 [-0.79, 0.97]
Anastasiou et al. 2004 i [50]	0.7%	0.83 [-0.10, 1.75]
Anastasiou et al. 2004 ii [50]	0.7%	0.31 [-0.57, 1.20]
Anastasiou et al. 2004 iii [50]	0.6%	1.01 [0.07, 1.96]
Angus et al. 2000 [51]	0.5%	1.23 [0.13, 2.33]
Bailey et al. 2000 i [52]	0.6%	0.57 [-0.38, 1.52]
Bailey et al. 2000 ii [52]	0.6%	0.34 [-0.59, 1.27]
Baur et al. 2014 i [54]	0.5%	0.78 [-0.25, 1.80]
Baur et al. 2014 ii [54]	0.6%	0.55 [-0.46, 1.55]
Baur et al. 2014 iii [54]	0.6%	0.23 [-0.75, 1.21]
Below et al. 1995 [55]	0.5%	0.77 [-0.26, 1.80]
Bishop et al. 2001 [56]	0.6%	0.89 [-0.09, 1.87]
Burgess et al. 1991 [57]	0.6%	0.18 [-0.74, 1.11]
Campbell et al. 2008 [59]	1.0%	0.19 [-0.50, 0.89]
Carter et al. 2003 i [60]	0.5%	0.70 [-0.39, 1.79]
Carter et al. 2003 ii [60]	0.6%	0.38 [-0.61, 1.37]
Carter et al. 2005 i [61]	0.6%	0.49 [-0.51, 1.49]
Carter et al. 2005 ii [61]	0.6%	0.44 [-0.56, 1.43]
Clark et al. 2000 [62]	0.5%	0.00 [-1.05, 1.05]
Cole et al. 1993 i [64]	0.7%	0.23 [-0.65, 1.11]
Cole et al. 1993 ii [64]	0.7%	0.22 [-0.66, 1.10]
Cole et al. 1993 iii [64]	0.7%	0.21 [-0.67, 1.09]
Cole et al. 2018 [65]	0.3%	3.96 [2.62, 5.30]
Cureton et al. 2007 [67]	1.0%	0.35 [-0.35, 1.05]
Currell et al. 2008 i [68]	0.3%	2.55 [1.14, 3.97]
Currell et al. 2008 ii [68]	0.2%	4.37 [2.37, 6.37]
Desbrow et al. 2004 [70]	0.7%	0.02 [-0.90, 0.95]
Duhamel et al. 2007 [71]	0.9%	0.85 [0.10, 1.60]
El-Sayed et al. 1995 [72]	0.6%	0.57 [-0.38, 1.52]
El-Sayed et al. 1997 [73]	0.6%	0.16 [-0.82, 1.14]
Fulco et al. 2007 i [75]	0.5%	0.58 [-0.48, 1.64]
Fulco et al. 2007 ii [75]	0.5%	0.43 [-0.62, 1.48]
Ganio et al. 2010 [77]	0.9%	0.42 [-0.33, 1.17]
Glace et al. 2019 i [78]	0.7%	0.28 [-0.60, 1.16]
Glace et al. 2019 ii [78]	0.7%	0.38 [-0.51, 1.26]
Glace et al. 2019 iii [78]	0.7%	0.38 [-0.51, 1.26]
Glace et al. 2019 iv [78]	0.7%	-0.02 [-0.89, 0.86]
Heesch et al. 2014 i [81]	0.6%	0.28 [-0.71, 1.26]
Heesch et al. 2014 ii [81]	0.6%	0.17 [-0.81, 1.15]
Heesch et al. 2014 iii [81]	0.6%	0.31 [-0.68, 1.30]
Hulston & Jeukendrup 2008 [82]	0.7%	0.50 [-0.40, 1.39]
Hulston & Jeukendrup 2009 [83]	0.6%	1.35 [0.36, 2.35]
Jarvis et al. 1999 [85]	0.7%	0.08 [-0.79, 0.96]
Jeukendrup et al. 1997 [86]	1.1%	0.54 [-0.11, 1.19]
Kang et al. 1995 [87]	0.5%	0.61 [-0.47, 1.69]
Kang et al. 1996 [88]	0.5%	0.48 [-0.59, 1.55]
King et al. 2018 i [90]	0.7%	0.43 [-0.46, 1.32]
King et al. 2018 ii [90]	0.7%	0.19 [-0.69, 1.07]
King et al. 2018 iii [90]	0.7%	0.83 [-0.10, 1.75]
King et al. 2018 iv [90]	0.7%	0.58 [-0.32, 1.48]
King et al. 2019 i [91]	0.7%	0.92 [0.03, 1.81]
King et al. 2019 ii [91]	0.7%	1.09 [0.18, 2.00]
King et al. 2019 iii [91]	0.7%	0.58 [-0.27, 1.44]
Kingwell et al. 1989 [92]	0.6%	0.53 [-0.42, 1.47]
Learsi et al. 2019 i [93]	0.6%	0.89 [-0.09, 1.87]
Learsi et al. 2019 ii [93]	0.4%	2.31 [1.05, 3.57]
Lugo et al. 1993 [94]	0.6%	0.88 [-0.10, 1.86]
Martínez-Lagunas et al. 2010 [95]	0.8%	0.69 [-0.14, 1.52]
Maughan et al. 1989 [96]	0.4%	0.51 [-0.65, 1.66]
McConell et al. 1996 [97]	0.5%	1.02 [-0.04, 2.08]
McConell et al. 1999 [98]	0.5%	0.97 [-0.08, 2.03]
McConell et al. 2000 [99]	0.9%	-0.08 [-0.85, 0.69]
Millard-Stafford et al. 2007 [103]	1.0%	0.34 [-0.36, 1.04]
Murray et al. 1987 i [105]	0.8%	0.63 [-0.16, 1.42]
Murray et al. 1987 ii [105]	0.8%	1.13 [0.29, 1.97]
Murray et al. 1987 iii [105]	0.7%	1.49 [0.60, 2.37]
Murray et al. 1989 i [106]	0.8%	0.57 [-0.25, 1.39]
Murray et al. 1989 ii [106]	0.8%	0.30 [-0.50, 1.11]
Murray et al. 1989 iii [106]	0.8%	0.05 [-0.75, 0.85]
Murray et al. 1991 i [107]	0.7%	0.60 [-0.30, 1.50]
Murray et al. 1991 ii [107]	0.7%	0.40 [-0.49, 1.29]
Murray et al. 1991 iii [107]	0.7%	0.49 [-0.40, 1.38]
Nassif et al. 2014 [109]	0.7%	-0.66 [-1.56, 0.25]
Newell et al. 2015 i [111]	1.2%	0.34 [-0.28, 0.97]
Newell et al. 2015 ii [111]	1.2%	0.59 [-0.05, 1.22]
Newell et al. 2015 iii [111]	1.2%	0.62 [-0.02, 1.25]
Nikolopoulos et al. 2004 [113]	0.6%	0.34 [-0.65, 1.33]
Nishibata et al. 1993 [114]	0.5%	-0.20 [-1.25, 0.85]
Oosthuyse et al. 2015a i [115]	0.7%	0.07 [-0.86, 0.99]
Oosthuyse et al. 2015a ii [115]	0.6%	-0.31 [-1.24, 0.62]
Oosthuyse et al. 2015b [116]	0.6%	0.15 [-0.83, 1.14]
Osterberg et al. 2008 [117]	0.8%	0.60 [-0.19, 1.39]
O'Hara et al. 2014 i [118]	0.7%	0.34 [-0.54, 1.22]
O'Hara et al. 2014 ii [118]	0.7%	-0.25 [-1.13, 0.63]
O'Neal et al. 2013 [119]	1.7%	0.10 [-0.36, 0.56]
Pottier et al. 2010 [121]	0.8%	-0.16 [-0.96, 0.64]
Rilley et al. 1988 [122]	0.6%	0.16 [-0.77, 1.08]
Roberts et al. 2014 i [123]	0.6%	0.50 [-0.47, 1.48]
Roberts et al. 2014 ii [123]	0.6%	-0.06 [-1.02, 0.90]
Smith et al. 2010 i [129]	0.8%	0.41 [-0.40, 1.22]
Smith et al. 2010 ii [129]	0.8%	0.49 [-0.32, 1.31]
Smith et al. 2010 iii [129]	0.8%	0.65 [-0.18, 1.47]
Steiner et al. 2009 [130]	0.7%	0.07 [-0.85, 1.00]
Van Essen et al. 2006 [138]	0.7%	0.19 [-0.69, 1.07]
Watson et al. 2012 i [140]	0.8%	0.19 [-0.61, 0.99]
Watson et al. 2012 ii [140]	0.8%	0.59 [-0.23, 1.41]
Watson et al. 2012 iii [140]	0.8%	0.60 [-0.22, 1.42]
Watson et al. 2012 iv [140]	0.8%	0.41 [-0.40, 1.22]
Watson et al. 2012 v [140]	0.8%	0.41 [-0.40, 1.22]
Watson et al. 2012 vi [140]	0.8%	0.63 [-0.19, 1.46]
**Subtotal (95% CI)**	**69.4%**	**0.47 [0.38, 0.57]**
Heterogeneity: $Tau^2 = 0.04$; $Chi^2 = 116.49$, df = 99 (P = 0.11); $I^2 = 15\%$		
Test for overall effect: Z = 9.62 (P < 0.00001)		
**Exercise mode: Running**		
Alghannam 2011 [48]	0.3%	1.67 [0.28, 3.07]
Ali et al. 2007 [49]	1.0%	0.22 [-0.47, 0.92]
Clarke et al. 2011 [63]	0.4%	2.90 [1.70, 4.10]
Coletta et al. 2013 i [66]	0.8%	0.08 [-0.72, 0.88]
Coletta et al. 2013 ii [66]	0.8%	-0.07 [-0.87, 0.73]
Davis et al. 2000 [69]	0.6%	0.49 [-0.51, 1.49]
Funnell et al. 2017 i [76]	1.0%	-0.28 [-0.97, 0.42]
Funnell et al. 2017 ii [76]	1.0%	-0.23 [-0.92, 0.47]
Goedecke et al. 2013 [79]	1.3%	0.39 [-0.21, 0.99]
Gui et al. 2017 [80]	0.7%	0.47 [-0.38, 1.32]
Khanna & Manna 2005 [89]	0.5%	2.10 [0.96, 3.24]
Millard-Stafford et al. 2005 i [102]	0.7%	0.50 [-0.39, 1.40]
Millard-Stafford et al. 2005 ii [102]	0.7%	0.62 [-0.29, 1.52]
Morris et al. 2003 [104]	0.6%	-0.31 [-1.24, 0.62]
Naclerio et al. 2014 [108]	0.7%	-0.01 [-0.89, 0.87]
Nassis et al. 1998 [110]	0.7%	-0.10 [-1.02, 0.83]
Nicholas et al. 1995 [112]	0.6%	0.55 [-0.40, 1.49]
Robson-Ansley et al. 2009 [124]	0.5%	0.44 [-0.63, 1.50]
Robson-Ansley et al. 2011 [125]	0.6%	0.16 [-0.76, 1.09]
Rollo & Williams 2009 [126]	0.6%	0.28 [-0.71, 1.26]
Rollo & Williams 2010 [127]	0.7%	-0.06 [-0.93, 0.82]
Rollo et al. 2011 [128]	0.7%	0.40 [-0.49, 1.29]
Sun et al. 2015 [131]	0.6%	0.62 [-0.39, 1.63]
Tokmakidis & Karamanolis 2008 [132]	0.8%	0.27 [-0.57, 1.11]
Tsintzas et al. 1993 [133]	0.5%	0.14 [-0.91, 1.19]
Tsintzas et al. 1995 i [134]	0.5%	0.13 [-0.92, 1.17]
Tsintzas et al. 1995 ii [134]	0.5%	0.31 [-0.75, 1.36]
Tsintzas et al. 1996a [135]	0.5%	0.89 [-0.16, 1.93]
Tsintzas et al. 1996b i [136]	0.7%	0.49 [-0.36, 1.34]
Tsintzas et al. 1996b ii [136]	0.8%	0.37 [-0.48, 1.21]
Utter et al. 2002 [137]	2.1%	0.04 [-0.36, 0.43]
Walton & Rhodes 1997 [139]	0.7%	0.68 [-0.23, 1.59]
Wilber et al. 1992 [141]	0.7%	0.85 [-0.08, 1.77]
Williams et al. 1990 i [142]	0.7%	0.27 [-0.60, 1.14]
Williams et al. 1990 ii [142]	0.7%	0.18 [-0.68, 1.05]
**Subtotal (95% CI)**	**25.4%**	**0.35 [0.17, 0.52]**
Heterogeneity: $Tau^2 = 0.08$; $Chi^2 = 48.04$, df = 34 (P = 0.06); $I^2 = 29\%$		
Test for overall effect: Z = 3.89 (P < 0.0001)		
**Exercise mode: Other**		
Baldassarre et al. 2021 [53]	0.5%	0.83 [-0.21, 1.86]
Byrne et al. 2005 [58]	0.9%	0.22 [-0.52, 0.97]
Finger et al. 2018 [74]	0.9%	0.04 [-0.73, 0.81]
Ivy et al. 1983 [84]	0.6%	0.86 [-0.07, 1.78]
McGawley et al. 2012 [100]	0.7%	0.40 [-0.48, 1.29]
Millard-Stafford et al. 1990 [101]	0.7%	0.14 [-0.74, 1.02]
Pettersson et al. 2019 [120]	0.8%	-0.06 [-0.86, 0.74]
**Subtotal (95% CI)**	**5.2%**	**0.29 [-0.03, 0.61]**
Heterogeneity: $Tau^2 = 0.00$; $Chi^2 = 3.83$, df = 6 (P = 0.70); $I^2 = 0\%$		
Test for overall effect: Z = 1.79 (P = 0.07)		
**Total (95% CI)**	**100.0%**	**0.43 [0.35, 0.51]**
Heterogeneity: $Tau^2 = 0.04$; $Chi^2 = 172.38$, df = 141 (P = 0.04); $I^2 = 18\%$		
Test for overall effect: Z = 10.31 (P < 0.00001)		
Test for subgroup differences: $Chi^2 = 2.32$, df = 2 (P = 0.31), $I^2 = 13.9\%$		

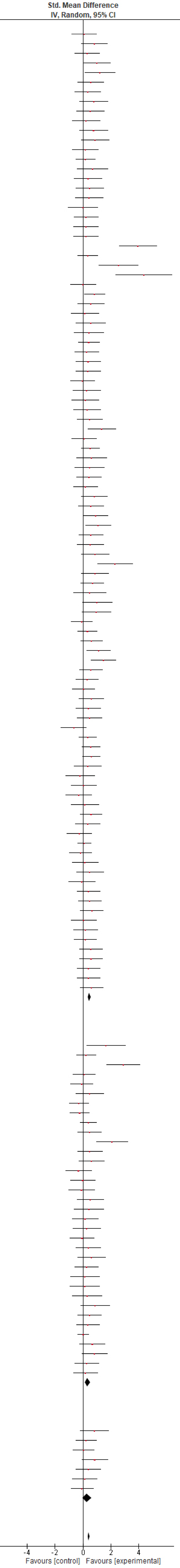

Figure S5: Forest plot shows the effects of experimental carbohydrate supplementation as compared to control on exercise outcome for 142 interventions. Subgroup analyses show the results with regard to exercise task in three exercise mode groups [Cycling, Running, Other (triathlon, duathlon, walking, loaded marching, roller-skiing)]. The black diamond symbol at the subgroups and at the bottom of the figure represents the standardized mean difference with the 95% confidence intervals for all interventions following random-effect meta-analyses. Studies or trials that provided insufficient data for subgroup classification were not included. Abbreviations: CI, confidence interval; IV, inverse variance; SD, standard deviation; Std, standardized; i-vi denote different intervention arms (trials) within the same study

Study or Subgroup	Weight	Std. Mean Difference IV, Random, 95% CI
**Capacity exercise test**		
Alghannam 2011 [48]	0.3%	1.67 [0.28, 3.07]
Bailey et al. 2000 i [52]	0.6%	0.57 [-0.38, 1.52]
Bailey et al. 2000 ii [52]	0.6%	0.34 [-0.59, 1.27]
Baldassarre et al. 2021 [53]	0.5%	0.83 [-0.21, 1.86]
Bishop et al. 2001 [56]	0.6%	0.89 [-0.09, 1.87]
Burgess et al. 1991 [57]	0.6%	0.18 [-0.74, 1.11]
Byrne et al. 2005 [58]	0.9%	0.22 [-0.52, 0.97]
Carter et al. 2003 i [60]	0.5%	0.70 [-0.39, 1.79]
Carter et al. 2003 ii [60]	0.6%	0.38 [-0.61, 1.37]
Carter et al. 2005 i [61]	0.6%	0.49 [-0.51, 1.49]
Carter et al. 2005 ii [61]	0.6%	0.44 [-0.56, 1.43]
Clarke et al. 2011 [63]	0.4%	2.90 [1.70, 4.10]
Cole et al. 1993 i [64]	0.7%	0.23 [-0.65, 1.11]
Cole et al. 1993 ii [64]	0.7%	0.22 [-0.66, 1.10]
Cole et al. 1993 iii [64]	0.7%	0.21 [-0.67, 1.09]
Cole et al. 2018 [65]	0.3%	3.96 [2.62, 5.30]
Cureton et al. 2007 [67]	1.0%	0.35 [-0.35, 1.05]
Currell et al. 2008 i [68]	0.3%	2.55 [1.14, 3.97]
Currell et al. 2008 ii [68]	0.2%	4.37 [2.37, 6.37]
Davis et al. 2000 [69]	0.6%	0.49 [-0.51, 1.49]
Duhamel et al. 2007 [71]	0.9%	0.85 [0.10, 1.60]
El-Sayed et al. 1995 [72]	0.6%	0.57 [-0.38, 1.52]
El-Sayed et al. 1997 [73]	0.6%	0.16 [-0.82, 1.14]
Funnell et al. 2017 i [76]	1.0%	-0.28 [-0.97, 0.42]
Funnell et al. 2017 ii [76]	1.0%	-0.23 [-0.92, 0.47]
Ganio et al. 2010 [77]	0.9%	0.42 [-0.33, 1.17]
Glace et al. 2019 iii [78]	0.7%	0.38 [-0.51, 1.26]
Glace et al. 2019 iv [78]	0.7%	-0.02 [-0.89, 0.86]
Goedecke et al. 2013 [79]	1.3%	0.39 [-0.21, 0.99]
Ivy et al. 1983 [84]	0.6%	0.86 [-0.07, 1.78]
Jarvis et al. 1999 [85]	0.7%	0.08 [-0.79, 0.96]
Kang et al. 1995 [87]	0.5%	0.61 [-0.47, 1.69]
Kang et al. 1996 [88]	0.5%	0.48 [-0.59, 1.55]
Khanna & Manna 2005 [89]	0.5%	2.10 [0.96, 3.24]
King et al. 2018 i [90]	0.7%	0.43 [-0.46, 1.32]
King et al. 2018 ii [90]	0.7%	0.19 [-0.69, 1.07]
King et al. 2018 iii [90]	0.7%	0.83 [-0.10, 1.75]
King et al. 2018 iv [90]	0.7%	0.58 [-0.32, 1.48]
King et al. 2019 i [91]	0.7%	0.92 [0.03, 1.81]
King et al. 2019 ii [91]	0.7%	1.09 [0.18, 2.00]
King et al. 2019 iii [91]	0.7%	0.58 [-0.27, 1.44]
Kingwell et al. 1989 [92]	0.6%	0.53 [-0.42, 1.47]
Martínez-Lagunas et al. 2010 [95]	0.8%	0.69 [-0.14, 1.52]
Maughan et al. 1989 [96]	0.4%	0.51 [-0.65, 1.66]
McConell et al. 1996 [97]	0.5%	1.02 [-0.04, 2.08]
McConell et al. 1999 [98]	0.5%	0.97 [-0.08, 2.03]
McConell et al. 2000 [99]	0.9%	-0.08 [-0.85, 0.69]
Millard-Stafford et al. 2007 [103]	1.0%	0.34 [-0.36, 1.04]
Morris et al. 2003 [104]	0.6%	-0.31 [-1.24, 0.62]
Nassis et al. 1998 [110]	0.7%	-0.10 [-1.02, 0.83]
Nicholas et al. 1995 [112]	0.6%	0.55 [-0.40, 1.49]
Nikolopoulos et al. 2004 [113]	0.6%	0.34 [-0.65, 1.33]
Nishibata et al. 1993 [114]	0.5%	-0.20 [-1.25, 0.85]
O'Hara et al. 2014 i [118]	0.7%	0.34 [-0.54, 1.22]
O'Hara et al. 2014 ii [118]	0.7%	-0.25 [-1.13, 0.63]
O'Neal et al. 2013 [119]	1.7%	0.10 [-0.36, 0.56]
Rilley et al. 1988 [122]	0.6%	0.16 [-0.77, 1.08]
Robson-Ansley et al. 2009 [124]	0.5%	0.44 [-0.63, 1.50]
Rollo & Williams 2009 [126]	0.6%	0.28 [-0.71, 1.26]
Rollo & Williams 2010 [127]	0.7%	-0.06 [-0.93, 0.82]
Rollo et al. 2011 [128]	0.7%	0.40 [-0.49, 1.29]
Steiner et al. 2009 [130]	0.7%	0.07 [-0.85, 1.00]
Sun et al. 2015 [131]	0.6%	0.62 [-0.39, 1.63]
Tokmakidis & Karamanolis 2008 [132]	0.8%	0.27 [-0.57, 1.11]
Tsintzas et al. 1996a [135]	0.5%	0.89 [-0.16, 1.93]
Tsintzas et al. 1996b i [136]	0.7%	0.49 [-0.36, 1.34]
Tsintzas et al. 1996b ii [136]	0.8%	0.37 [-0.48, 1.21]
Walton & Rhodes 1997 [139]	0.7%	0.68 [-0.23, 1.59]
Watson et al. 2012 i [140]	0.8%	0.19 [-0.61, 0.99]
Watson et al. 2012 ii [140]	0.8%	0.59 [-0.23, 1.41]
Watson et al. 2012 iii [140]	0.8%	0.60 [-0.22, 1.42]
Watson et al. 2012 iv [140]	0.8%	0.41 [-0.40, 1.22]
Watson et al. 2012 v [140]	0.8%	0.41 [-0.40, 1.22]
Watson et al. 2012 vi [140]	0.8%	0.63 [-0.19, 1.46]
Wilber et al. 1992 [141]	0.7%	0.85 [-0.08, 1.77]
**Subtotal (95% CI)**	**51.1%**	**0.49 [0.36, 0.62]**
Heterogeneity: Tau² = 0.12; Chi² = 115.44, df = 74 (P = 0.001); I² = 36%		
Test for overall effect: Z = 7.45 (P < 0.00001)		
**Performance exercise test**		
Acker-Hewitt et al. 2012 [47]	0.7%	0.09 [-0.79, 0.97]
Ali et al. 2007 [49]	1.0%	0.22 [-0.47, 0.92]
Anastasiou et al. 2004 i [50]	0.7%	0.83 [-0.10, 1.75]
Anastasiou et al. 2004 ii [50]	0.7%	0.31 [-0.57, 1.20]
Anastasiou et al. 2004 iii [50]	0.6%	1.01 [0.07, 1.96]
Angus et al. 2000 [51]	0.5%	1.23 [0.13, 2.33]
Baur et al. 2014 i [54]	0.5%	0.78 [-0.25, 1.80]
Baur et al. 2014 ii [54]	0.6%	0.55 [-0.46, 1.55]
Baur et al. 2014 iii [54]	0.6%	0.23 [-0.75, 1.21]
Below et al. 1995 [55]	0.5%	0.77 [-0.26, 1.80]
Campbell et al. 2008 [59]	1.0%	0.19 [-0.50, 0.89]
Clark et al. 2000 [62]	0.5%	0.00 [-1.05, 1.05]
Coletta et al. 2013 i [66]	0.8%	0.08 [-0.72, 0.88]
Coletta et al. 2013 ii [66]	0.8%	-0.07 [-0.87, 0.73]
Desbrow et al. 2004 [70]	0.7%	0.02 [-0.90, 0.95]
Finger et al. 2018 [74]	0.9%	0.04 [-0.73, 0.81]
Fulco et al. 2007 i [75]	0.5%	0.58 [-0.48, 1.64]
Fulco et al. 2007 ii [75]	0.5%	0.43 [-0.62, 1.48]
Glace et al. 2019 i [78]	0.7%	0.28 [-0.60, 1.16]
Glace et al. 2019 ii [78]	0.7%	0.38 [-0.51, 1.26]
Gui et al. 2017 [80]	0.7%	0.47 [-0.38, 1.32]
Heesch et al. 2014 i [81]	0.6%	0.28 [-0.71, 1.26]
Heesch et al. 2014 ii [81]	0.6%	0.17 [-0.81, 1.15]
Heesch et al. 2014 iii [81]	0.6%	0.31 [-0.68, 1.30]
Hulston & Jeukendrup 2008 [82]	0.7%	0.50 [-0.40, 1.39]
Hulston & Jeukendrup 2009 [83]	0.6%	1.35 [0.36, 2.35]
Jeukendrup et al. 1997 [86]	1.1%	0.54 [-0.11, 1.19]
Learsi et al. 2019 i [93]	0.6%	0.89 [-0.09, 1.87]
Learsi et al. 2019 ii [93]	0.4%	2.31 [1.05, 3.57]
Lugo et al. 1993 [94]	0.6%	0.88 [-0.10, 1.86]
McGawley et al. 2012 [100]	0.7%	0.40 [-0.48, 1.29]
Millard-Stafford et al. 1990 [101]	0.7%	0.14 [-0.74, 1.02]
Millard-Stafford et al. 2005 i [102]	0.7%	0.50 [-0.39, 1.40]
Millard-Stafford et al. 2005 ii [102]	0.7%	0.62 [-0.29, 1.52]
Murray et al. 1987 i [105]	0.8%	0.63 [-0.16, 1.42]
Murray et al. 1987 ii [105]	0.8%	1.13 [0.29, 1.97]
Murray et al. 1987 iii [105]	0.7%	1.49 [0.60, 2.37]
Murray et al. 1989 i [106]	0.8%	0.57 [-0.25, 1.39]
Murray et al. 1989 ii [106]	0.8%	0.30 [-0.50, 1.11]
Murray et al. 1989 iii [106]	0.8%	0.05 [-0.75, 0.85]
Murray et al. 1991 i [107]	0.7%	0.60 [-0.30, 1.50]
Murray et al. 1991 ii [107]	0.7%	0.40 [-0.49, 1.29]
Murray et al. 1991 iii [107]	0.7%	0.49 [-0.40, 1.38]
Naclerio et al. 2014 [108]	0.7%	-0.01 [-0.89, 0.87]
Nassif et al. 2014 [109]	0.7%	-0.66 [-1.56, 0.25]
Newell et al. 2015 i [111]	1.2%	0.34 [-0.28, 0.97]
Newell et al. 2015 ii [111]	1.2%	0.59 [-0.05, 1.22]
Newell et al. 2015 iii [111]	1.2%	0.62 [-0.02, 1.25]
Oosthuyse et al. 2015a i [115]	0.7%	0.07 [-0.86, 0.99]
Oosthuyse et al. 2015a ii [115]	0.6%	-0.31 [-1.24, 0.62]
Oosthuyse et al. 2015b [116]	0.6%	0.15 [-0.83, 1.14]
Osterberg et al. 2008 [117]	0.8%	0.60 [-0.19, 1.39]
Pettersson et al. 2019 [120]	0.8%	-0.06 [-0.86, 0.74]
Pottier et al. 2010 [121]	0.8%	-0.16 [-0.96, 0.64]
Roberts et al. 2014 i [123]	0.6%	0.50 [-0.47, 1.48]
Roberts et al. 2014 ii [123]	0.6%	-0.06 [-1.02, 0.90]
Robson-Ansley et al. 2011 [125]	0.6%	0.16 [-0.76, 1.09]
Smith et al. 2010 i [129]	0.8%	0.41 [-0.40, 1.22]
Smith et al. 2010 ii [129]	0.8%	0.49 [-0.32, 1.31]
Smith et al. 2010 iii [129]	0.8%	0.65 [-0.18, 1.47]
Tsintzas et al. 1993 [133]	0.5%	0.14 [-0.91, 1.19]
Tsintzas et al. 1995 i [134]	0.5%	0.13 [-0.92, 1.17]
Tsintzas et al. 1995 ii [134]	0.5%	0.31 [-0.75, 1.36]
Utter et al. 2002 [137]	2.1%	0.04 [-0.36, 0.43]
Van Essen et al. 2006 [138]	0.7%	0.19 [-0.69, 1.07]
Williams et al. 1990 i [142]	0.7%	0.27 [-0.60, 1.14]
Williams et al. 1990 ii [142]	0.7%	0.18 [-0.68, 1.05]
**Subtotal (95% CI)**	**48.9%**	**0.37 [0.27, 0.47]**
Heterogeneity: Tau² = 0.00; Chi² = 55.63, df = 66 (P = 0.81); I² = 0%		
Test for overall effect: Z = 7.01 (P < 0.00001)		
**Total (95% CI)**	**100.0%**	**0.43 [0.35, 0.51]**
Heterogeneity: Tau² = 0.04; Chi² = 172.38, df = 141 (P = 0.04); I² = 18%		
Test for overall effect: Z = 10.31 (P < 0.00001)		
Test for subgroup differences: Chi² = 2.16, df = 1 (P = 0.14), I² = 53.6%		

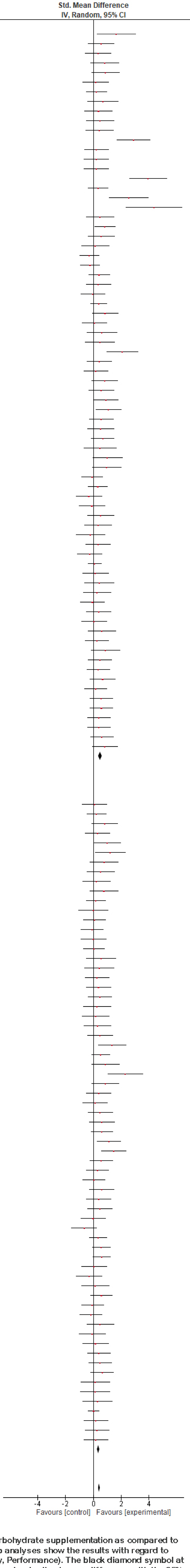

Figure S6: Forest plot shows the effects of experimental carbohydrate supplementation as compared to control on exercise outcome for 142 interventions. Subgroup analyses show the results with regard to exercise task in two exercise protocol test groups (Capacity, Performance). The black diamond symbol at the subgroups and at the bottom of the figure represents the standardized mean difference with the 95% confidence intervals for all interventions following random-effect meta-analyses. Studies or trials that provided insufficient data for subgroup classification were not included. Abbreviations: CI, confidence interval; IV, inverse variance; SD, standard deviation; Std, standardized; i-vi denote different intervention arms (trials) within the same study

Study or Subgroup	Weight	Std. Mean Difference IV, Random, 95% CI
**Continuous exercise**		
Acker-Hewitt et al. 2012 [47]	0.7%	0.09 [-0.79, 0.97]
Anastasiou et al. 2004 i [50]	0.7%	0.83 [-0.10, 1.75]
Anastasiou et al. 2004 ii [50]	0.7%	0.31 [-0.57, 1.20]
Anastasiou et al. 2004 iii [50]	0.6%	1.01 [0.07, 1.96]
Angus et al. 2000 [51]	0.5%	1.23 [0.13, 2.33]
Bailey et al. 2000 i [52]	0.6%	0.57 [-0.38, 1.52]
Bailey et al. 2000 ii [52]	0.6%	0.34 [-0.59, 1.27]
Baldassarre et al. 2021 [53]	0.5%	0.83 [-0.21, 1.86]
Baur et al. 2014 i [54]	0.5%	0.78 [-0.25, 1.80]
Baur et al. 2014 ii [54]	0.6%	0.55 [-0.46, 1.55]
Baur et al. 2014 iii [54]	0.6%	0.23 [-0.75, 1.21]
Below et al. 1995 [55]	0.5%	0.77 [-0.26, 1.80]
Bishop et al. 2001 [56]	0.6%	0.89 [-0.09, 1.87]
Burgess et al. 1991 [57]	0.6%	0.18 [-0.74, 1.11]
Campbell et al. 2008 [59]	1.0%	0.19 [-0.50, 0.89]
Carter et al. 2003 i [60]	0.5%	0.70 [-0.39, 1.79]
Carter et al. 2003 ii [60]	0.6%	0.38 [-0.61, 1.37]
Carter et al. 2005 i [61]	0.6%	0.49 [-0.51, 1.49]
Carter et al. 2005 ii [61]	0.6%	0.44 [-0.56, 1.43]
Clark et al. 2000 [62]	0.5%	0.00 [-1.05, 1.05]
Cole et al. 1993 i [64]	0.7%	0.23 [-0.65, 1.11]
Cole et al. 1993 ii [64]	0.7%	0.22 [-0.66, 1.10]
Cole et al. 1993 iii [64]	0.7%	0.21 [-0.67, 1.09]
Cole et al. 2018 [65]	0.3%	3.96 [2.62, 5.30]
Coletta et al. 2013 i [66]	0.8%	0.08 [-0.72, 0.88]
Coletta et al. 2013 ii [66]	0.8%	-0.07 [-0.87, 0.73]
Cureton et al. 2007 [67]	1.0%	0.35 [-0.35, 1.05]
Currell et al. 2008 i [68]	0.3%	2.55 [1.14, 3.97]
Currell et al. 2008 ii [68]	0.2%	4.37 [2.37, 6.37]
Desbrow et al. 2004 [70]	0.7%	0.02 [-0.90, 0.95]
Duhamel et al. 2007 [71]	0.9%	0.85 [0.10, 1.60]
El-Sayed et al. 1995 [72]	0.6%	0.57 [-0.38, 1.52]
El-Sayed et al. 1997 [73]	0.6%	0.16 [-0.82, 1.14]
Finger et al. 2018 [74]	0.9%	0.04 [-0.73, 0.81]
Fulco et al. 2007 i [75]	0.5%	0.58 [-0.48, 1.64]
Fulco et al. 2007 ii [75]	0.5%	0.43 [-0.62, 1.48]
Ganio et al. 2010 [77]	0.9%	0.42 [-0.33, 1.17]
Glace et al. 2019 i [78]	0.7%	0.28 [-0.60, 1.16]
Glace et al. 2019 ii [78]	0.7%	0.38 [-0.51, 1.26]
Glace et al. 2019 iii [78]	0.7%	0.38 [-0.51, 1.26]
Glace et al. 2019 iv [78]	0.7%	-0.02 [-0.89, 0.86]
Gui et al. 2017 [80]	0.7%	0.47 [-0.38, 1.32]
Heesch et al. 2014 i [81]	0.6%	0.28 [-0.71, 1.26]
Heesch et al. 2014 ii [81]	0.6%	0.17 [-0.81, 1.15]
Heesch et al. 2014 iii [81]	0.6%	0.31 [-0.68, 1.30]
Hulston & Jeukendrup 2008 [82]	0.7%	0.50 [-0.40, 1.39]
Hulston & Jeukendrup 2009 [83]	0.6%	1.35 [0.36, 2.35]
Ivy et al. 1983 [84]	0.6%	0.86 [-0.07, 1.78]
Jarvis et al. 1999 [85]	0.7%	0.08 [-0.79, 0.96]
Jeukendrup et al. 1997 [86]	1.1%	0.54 [-0.11, 1.19]
Kang et al. 1995 [87]	0.5%	0.61 [-0.47, 1.69]
Kang et al. 1996 [88]	0.5%	0.48 [-0.59, 1.55]
Khanna & Manna 2005 [89]	0.5%	2.10 [0.96, 3.24]
King et al. 2019 i [91]	0.7%	0.92 [0.03, 1.81]
King et al. 2019 ii [91]	0.7%	1.09 [0.18, 2.00]
King et al. 2019 iii [91]	0.7%	0.58 [-0.27, 1.44]
Kingwell et al. 1989 [92]	0.6%	0.53 [-0.42, 1.47]
Learsi et al. 2019 i [93]	0.6%	0.89 [-0.09, 1.87]
Learsi et al. 2019 ii [93]	0.4%	2.31 [1.05, 3.57]
Lugo et al. 1993 [94]	0.6%	0.88 [-0.10, 1.86]
Martínez-Lagunas et al. 2010 [95]	0.8%	0.69 [-0.14, 1.52]
Maughan et al. 1989 [96]	0.4%	0.51 [-0.65, 1.66]
McConell et al. 1996 [97]	0.5%	1.02 [-0.04, 2.08]
McConell et al. 1999 [98]	0.5%	0.97 [-0.08, 2.03]
McConell et al. 2000 [99]	0.9%	-0.08 [-0.85, 0.69]
McGawley et al. 2012 [100]	0.7%	0.40 [-0.48, 1.29]
Millard-Stafford et al. 1990 [101]	0.7%	0.14 [-0.74, 1.02]
Millard-Stafford et al. 2005 i [102]	0.7%	0.50 [-0.39, 1.40]
Millard-Stafford et al. 2005 ii [102]	0.7%	0.62 [-0.29, 1.52]
Millard-Stafford et al. 2007 [103]	1.0%	0.34 [-0.36, 1.04]
Murray et al. 1991 i [107]	0.7%	0.60 [-0.30, 1.50]
Murray et al. 1991 ii [107]	0.7%	0.40 [-0.49, 1.29]
Murray et al. 1991 iii [107]	0.7%	0.49 [-0.40, 1.38]
Nassif et al. 2014 [109]	0.7%	-0.66 [-1.56, 0.25]
Newell et al. 2015 i [111]	1.2%	0.34 [-0.28, 0.97]
Newell et al. 2015 ii [111]	1.2%	0.59 [-0.05, 1.22]
Newell et al. 2015 iii [111]	1.2%	0.62 [-0.02, 1.25]
Nikolopoulos et al. 2004 [113]	0.6%	0.34 [-0.65, 1.33]
Nishibata et al. 1993 [114]	0.5%	-0.20 [-1.25, 0.85]
Oosthuyse et al. 2015a i [115]	0.7%	0.07 [-0.86, 0.99]
Oosthuyse et al. 2015a ii [115]	0.6%	-0.31 [-1.24, 0.62]
Oosthuyse et al. 2015b [116]	0.6%	0.15 [-0.83, 1.14]
Osterberg et al. 2008 [117]	0.8%	0.60 [-0.19, 1.39]
Pettersson et al. 2019 [120]	0.8%	-0.06 [-0.86, 0.74]
Pottier et al. 2010 [121]	0.8%	-0.16 [-0.96, 0.64]
Rilley et al. 1988 [122]	0.6%	0.16 [-0.77, 1.08]
Roberts et al. 2014 i [123]	0.6%	0.50 [-0.47, 1.48]
Roberts et al. 2014 ii [123]	0.6%	-0.06 [-1.02, 0.90]
Robson-Ansley et al. 2009 [124]	0.5%	0.44 [-0.63, 1.50]
Robson-Ansley et al. 2011 [125]	0.6%	0.16 [-0.76, 1.09]
Rollo & Williams 2009 [126]	0.6%	0.28 [-0.71, 1.26]
Rollo & Williams 2010 [127]	0.7%	-0.06 [-0.93, 0.82]
Rollo et al. 2011 [128]	0.7%	0.40 [-0.49, 1.29]
Smith et al. 2010 i [129]	0.8%	0.41 [-0.40, 1.22]
Smith et al. 2010 ii [129]	0.8%	0.49 [-0.32, 1.31]
Smith et al. 2010 iii [129]	0.8%	0.65 [-0.18, 1.47]
Steiner et al. 2009 [130]	0.7%	0.07 [-0.85, 1.00]
Sun et al. 2015 [131]	0.6%	0.62 [-0.39, 1.63]
Tokmakidis & Karamanolis 2008 [132]	0.8%	0.27 [-0.57, 1.11]
Tsintzas et al. 1993 [133]	0.5%	0.14 [-0.91, 1.19]
Tsintzas et al. 1995 i [134]	0.5%	0.13 [-0.92, 1.17]
Tsintzas et al. 1995 ii [134]	0.5%	0.31 [-0.75, 1.36]
Tsintzas et al. 1996a [135]	0.5%	0.89 [-0.16, 1.93]
Tsintzas et al. 1996b i [136]	0.7%	0.49 [-0.36, 1.34]
Tsintzas et al. 1996b ii [136]	0.8%	0.37 [-0.48, 1.21]
Utter et al. 2002 [137]	2.1%	0.04 [-0.36, 0.43]
Van Essen et al. 2006 [138]	0.7%	0.19 [-0.69, 1.07]
Watson et al. 2012 i [140]	0.8%	0.19 [-0.61, 0.99]
Watson et al. 2012 ii [140]	0.8%	0.59 [-0.23, 1.41]
Watson et al. 2012 iii [140]	0.8%	0.60 [-0.22, 1.42]
Watson et al. 2012 iv [140]	0.8%	0.41 [-0.40, 1.22]
Watson et al. 2012 v [140]	0.8%	0.41 [-0.40, 1.22]
Watson et al. 2012 vi [140]	0.8%	0.63 [-0.19, 1.46]
Wilber et al. 1992 [141]	0.7%	0.85 [-0.08, 1.77]
Williams et al. 1990 i [142]	0.7%	0.27 [-0.60, 1.14]
Williams et al. 1990 ii [142]	0.7%	0.18 [-0.68, 1.05]
**Subtotal (95% CI)**	**79.5%**	**0.44 [0.35, 0.52]**
Heterogeneity: Tau² = 0.02; Chi² = 125.45, df = 115 (P = 0.24); I² = 8%		
Test for overall effect: Z = 9.90 (P < 0.00001)		
**Intermittent exercise**		
Byrne et al. 2005 [58]	0.9%	0.22 [-0.52, 0.97]
Davis et al. 2000 [69]	0.6%	0.49 [-0.51, 1.49]
King et al. 2018 i [90]	0.7%	0.43 [-0.46, 1.32]
King et al. 2018 ii [90]	0.7%	0.19 [-0.69, 1.07]
King et al. 2018 iii [90]	0.7%	0.83 [-0.10, 1.75]
King et al. 2018 iv [90]	0.7%	0.58 [-0.32, 1.48]
Morris et al. 2003 [104]	0.6%	-0.31 [-1.24, 0.62]
Murray et al. 1987 i [105]	0.8%	0.63 [-0.16, 1.42]
Murray et al. 1987 ii [105]	0.8%	1.13 [0.29, 1.97]
Murray et al. 1987 iii [105]	0.7%	1.49 [0.60, 2.37]
Murray et al. 1989 i [106]	0.8%	0.57 [-0.25, 1.39]
Murray et al. 1989 ii [106]	0.8%	0.30 [-0.50, 1.11]
Murray et al. 1989 iii [106]	0.8%	0.05 [-0.75, 0.85]
Nassis et al. 1998 [110]	0.7%	-0.10 [-1.02, 0.83]
Nicholas et al. 1995 [112]	0.6%	0.55 [-0.40, 1.49]
O'Hara et al. 2014 i [118]	0.7%	0.34 [-0.54, 1.22]
O'Hara et al. 2014 ii [118]	0.7%	-0.25 [-1.13, 0.63]
O'Neal et al. 2013 [119]	1.7%	0.10 [-0.36, 0.56]
Walton & Rhodes 1997 [139]	0.7%	0.68 [-0.23, 1.59]
**Subtotal (95% CI)**	**14.7%**	**0.38 [0.19, 0.58]**
Heterogeneity: Tau² = 0.01; Chi² = 18.93, df = 18 (P = 0.40); I² = 5%		
Test for overall effect: Z = 3.90 (P < 0.0001)		
**Intermittent shuttle exercise**		
Alghannam 2011 [48]	0.3%	1.67 [0.28, 3.07]
Ali et al. 2007 [49]	1.0%	0.22 [-0.47, 0.92]
Clarke et al. 2011 [63]	0.4%	2.90 [1.70, 4.10]
Funnell et al. 2017 i [76]	1.0%	-0.28 [-0.97, 0.42]
Funnell et al. 2017 ii [76]	1.0%	-0.23 [-0.92, 0.47]
Goedecke et al. 2013 [79]	1.3%	0.39 [-0.21, 0.99]
Naclerio et al. 2014 [108]	0.7%	-0.01 [-0.89, 0.87]
**Subtotal (95% CI)**	**5.8%**	**0.53 [-0.13, 1.18]**
Heterogeneity: Tau² = 0.58; Chi² = 27.10, df = 6 (P = 0.0001); I² = 78%		
Test for overall effect: Z = 1.58 (P = 0.11)		
**Total (95% CI)**	**100.0%**	**0.43 [0.35, 0.51]**
Heterogeneity: Tau² = 0.04; Chi² = 172.38, df = 141 (P = 0.04); I² = 18%		
Test for overall effect: Z = 10.31 (P < 0.00001)		
Test for subgroup differences: Chi² = 0.31, df = 2 (P = 0.85), I² = 0%		

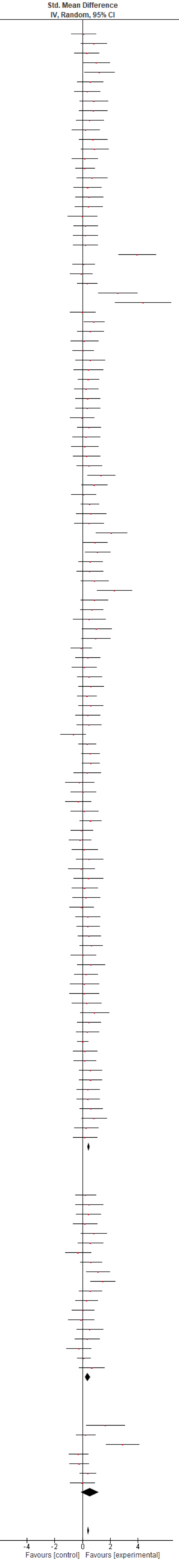

Figure S7: Forest plot shows the effects of experimental carbohydrate supplementation as compared to control on exercise outcome for 142 interventions. Subgroup analyses show the results with regard to exercise task in three exercise type groups (Intermittent, Continuous, Intermittent shuttle). The black diamond symbol at the subgroups and at the bottom of the figure represents the standardized mean difference with the 95% confidence intervals for all interventions following random-effect meta-analyses. Studies or trials that provided insufficient data for subgroup classification were not included. Abbreviations: CI, confidence interval; IV, inverse variance; SD, standard deviation; Std, standardized; i-vi denote different intervention arms (trials) within the same study

Study or Subgroup	Weight	Std. Mean Difference IV, Random, 95% CI
**Concentration: 0% < CHO ≤ 2%**		
Burgess et al. 1991 [57]	0.6%	0.18 [-0.74, 1.11]
Newell et al. 2015 i [111]	1.2%	0.34 [-0.28, 0.97]
Smith et al. 2010 i [129]	0.8%	0.41 [-0.40, 1.22]
Watson et al. 2012 i [140]	0.8%	0.19 [-0.61, 0.99]
Watson et al. 2012 iv [140]	0.8%	0.41 [-0.40, 1.22]
**Subtotal (95% CI)**	**4.3%**	**0.32 [-0.03, 0.66]**
Heterogeneity: $Tau^2 = 0.00$; $Chi^2 = 0.29$, df = 4 (P = 0.99); $I^2 = 0\%$		
Test for overall effect: Z = 1.79 (P = 0.07)		
**Concentration: 2% < CHO ≤ 4%**		
Heesch et al. 2014 i [81]	0.6%	0.28 [-0.71, 1.26]
Maughan et al. 1989 [96]	0.4%	0.51 [-0.65, 1.66]
Newell et al. 2015 ii [111]	1.2%	0.59 [-0.05, 1.22]
O'Hara et al. 2014 i [118]	0.7%	0.34 [-0.54, 1.22]
O'Hara et al. 2014 ii [118]	0.7%	-0.25 [-1.13, 0.63]
Watson et al. 2012 ii [140]	0.8%	0.59 [-0.23, 1.41]
Watson et al. 2012 v [140]	0.8%	0.41 [-0.40, 1.22]
**Subtotal (95% CI)**	**5.2%**	**0.38 [0.06, 0.70]**
Heterogeneity: $Tau^2 = 0.00$; $Chi^2 = 2.71$, df = 6 (P = 0.84); $I^2 = 0\%$		
Test for overall effect: Z = 2.34 (P = 0.02)		
**Concentration: 4% < CHO ≤ 6%**		
Angus et al. 2000 [51]	0.5%	1.23 [0.13, 2.33]
Bailey et al. 2000 i [52]	0.6%	0.57 [-0.38, 1.52]
Bailey et al. 2000 ii [52]	0.6%	0.34 [-0.59, 1.27]
Below et al. 1995 [55]	0.5%	0.77 [-0.26, 1.80]
Bishop et al. 2001 [56]	0.6%	0.89 [-0.09, 1.87]
Byrne et al. 2005 [58]	0.9%	0.22 [-0.52, 0.97]
Campbell et al. 2008 [59]	1.0%	0.19 [-0.50, 0.89]
Cole et al. 1993 i [64]	0.7%	0.23 [-0.65, 1.11]
Cole et al. 2018 [65]	0.3%	3.96 [2.62, 5.30]
Coletta et al. 2013 i [66]	0.8%	0.08 [-0.72, 0.88]
Cureton et al. 2007 [67]	1.0%	0.35 [-0.35, 1.05]
Davis et al. 2000 [69]	0.6%	0.49 [-0.51, 1.49]
Desbrow et al. 2004 [70]	0.7%	0.02 [-0.90, 0.95]
Duhamel et al. 2007 [71]	0.9%	0.85 [0.10, 1.60]
Ganio et al. 2010 [77]	0.9%	0.42 [-0.33, 1.17]
Glace et al. 2019 i [78]	0.7%	0.28 [-0.60, 1.16]
Glace et al. 2019 ii [78]	0.7%	0.38 [-0.51, 1.26]
Glace et al. 2019 iii [78]	0.7%	0.38 [-0.51, 1.26]
Glace et al. 2019 iv [78]	0.7%	-0.02 [-0.89, 0.86]
Gui et al. 2017 [80]	0.7%	0.47 [-0.38, 1.32]
Heesch et al. 2014 ii [81]	0.6%	0.17 [-0.81, 1.15]
Heesch et al. 2014 iii [81]	0.6%	0.31 [-0.68, 1.30]
Hulston & Jeukendrup 2009 [83]	0.6%	1.35 [0.36, 2.35]
Kang et al. 1995 [87]	0.5%	0.61 [-0.47, 1.69]
Kang et al. 1996 [88]	0.5%	0.48 [-0.59, 1.55]
Khanna & Manna 2005 [89]	0.5%	2.10 [0.96, 3.24]
King et al. 2018 i [90]	0.7%	0.43 [-0.46, 1.32]
Martínez-Lagunas et al. 2010 [95]	0.8%	0.69 [-0.14, 1.52]
McConell et al. 2000 [99]	0.9%	-0.08 [-0.85, 0.69]
Millard-Stafford et al. 2005 i [102]	0.7%	0.50 [-0.39, 1.40]
Millard-Stafford et al. 2007 [103]	1.0%	0.34 [-0.36, 1.04]
Murray et al. 1987 i [105]	0.8%	0.63 [-0.16, 1.42]
Murray et al. 1987 ii [105]	0.8%	1.13 [0.29, 1.97]
Murray et al. 1989 i [106]	0.8%	0.57 [-0.25, 1.39]
Murray et al. 1991 i [107]	0.7%	0.60 [-0.30, 1.50]
Nassif et al. 2014 [109]	0.7%	-0.66 [-1.56, 0.25]
Osterberg et al. 2008 [117]	0.8%	0.60 [-0.19, 1.39]
O'Neal et al. 2013 [119]	1.7%	0.10 [-0.36, 0.56]
Pottier et al. 2010 [121]	0.8%	-0.16 [-0.96, 0.64]
Smith et al. 2010 ii [129]	0.8%	0.49 [-0.32, 1.31]
Smith et al. 2010 iii [129]	0.8%	0.65 [-0.18, 1.47]
Steiner et al. 2009 [130]	0.7%	0.07 [-0.85, 1.00]
Sun et al. 2015 [131]	0.6%	0.62 [-0.39, 1.63]
Tsintzas et al. 1993 [133]	0.5%	0.14 [-0.91, 1.19]
Tsintzas et al. 1995 ii [134]	0.5%	0.31 [-0.75, 1.36]
Tsintzas et al. 1996a [135]	0.5%	0.89 [-0.16, 1.93]
Tsintzas et al. 1996b i [136]	0.7%	0.49 [-0.36, 1.34]
Utter et al. 2002 [137]	2.1%	0.04 [-0.36, 0.43]
Van Essen et al. 2006 [138]	0.7%	0.19 [-0.69, 1.07]
Watson et al. 2012 iii [140]	0.8%	0.60 [-0.22, 1.42]
Watson et al. 2012 vi [140]	0.8%	0.63 [-0.19, 1.46]
Williams et al. 1990 i [142]	0.7%	0.27 [-0.60, 1.14]
Williams et al. 1990 ii [142]	0.7%	0.18 [-0.68, 1.05]
**Subtotal (95% CI)**	**39.6%**	**0.44 [0.31, 0.57]**
Heterogeneity: $Tau^2 = 0.06$; $Chi^2 = 68.76$, df = 52 (P = 0.06); $I^2 = 24\%$		
Test for overall effect: Z = 6.42 (P < 0.00001)		
**Concentration: 6% < CHO ≤ 8%**		
Acker-Hewitt et al. 2012 [47]	0.7%	0.09 [-0.79, 0.97]
Alghannam 2011 [48]	0.3%	1.67 [0.28, 3.07]
Ali et al. 2007 [49]	1.0%	0.22 [-0.47, 0.92]
Anastasiou et al. 2004 i [50]	0.7%	0.83 [-0.10, 1.75]
Anastasiou et al. 2004 ii [50]	0.7%	0.31 [-0.57, 1.20]
Anastasiou et al. 2004 iii [50]	0.6%	1.01 [0.07, 1.96]
Baldassarre et al. 2021 [53]	0.5%	0.83 [-0.21, 1.86]
Baur et al. 2014 ii [54]	0.6%	0.55 [-0.46, 1.55]
Carter et al. 2003 i [60]	0.5%	0.70 [-0.39, 1.79]
Carter et al. 2003 ii [60]	0.6%	0.38 [-0.61, 1.37]
Carter et al. 2005 i [61]	0.6%	0.49 [-0.51, 1.49]
Carter et al. 2005 ii [61]	0.6%	0.44 [-0.56, 1.43]
Clark et al. 2000 [62]	0.5%	0.00 [-1.05, 1.05]
Clarke et al. 2011 [63]	0.4%	2.90 [1.70, 4.10]
Coletta et al. 2013 ii [66]	0.8%	-0.07 [-0.87, 0.73]
El-Sayed et al. 1995 [72]	0.6%	0.57 [-0.38, 1.52]
El-Sayed et al. 1997 [73]	0.6%	0.16 [-0.82, 1.14]
Goedecke et al. 2013 [79]	1.3%	0.39 [-0.21, 0.99]
Hulston & Jeukendrup 2008 [82]	0.7%	0.50 [-0.40, 1.39]
Jarvis et al. 1999 [85]	0.7%	0.08 [-0.79, 0.96]
Jeukendrup et al. 1997 [86]	1.1%	0.54 [-0.11, 1.19]
King et al. 2018 ii [90]	0.7%	0.19 [-0.69, 1.07]
King et al. 2019 i [91]	0.7%	0.92 [0.03, 1.81]
Learsi et al. 2019 i [93]	0.6%	0.89 [-0.09, 1.87]
Learsi et al. 2019 ii [93]	0.4%	2.31 [1.05, 3.57]
Lugo et al. 1993 [94]	0.6%	0.88 [-0.10, 1.86]
McConell et al. 1996 [97]	0.5%	1.02 [-0.04, 2.08]
McConell et al. 1999 [98]	0.5%	0.97 [-0.08, 2.03]
Millard-Stafford et al. 1990 [101]	0.7%	0.14 [-0.74, 1.02]
Millard-Stafford et al. 2005 ii [102]	0.7%	0.62 [-0.29, 1.52]
Morris et al. 2003 [104]	0.6%	-0.31 [-1.24, 0.62]
Murray et al. 1987 iii [105]	0.7%	1.49 [0.60, 2.37]
Murray et al. 1989 ii [106]	0.8%	0.30 [-0.50, 1.11]
Nassis et al. 1998 [110]	0.7%	-0.10 [-1.02, 0.83]
Newell et al. 2015 iii [111]	1.2%	0.62 [-0.02, 1.25]
Nicholas et al. 1995 [112]	0.6%	0.55 [-0.40, 1.49]
Nikolopoulos et al. 2004 [113]	0.6%	0.34 [-0.65, 1.33]
Oosthuyse et al. 2015a i [115]	0.7%	0.07 [-0.86, 0.99]
Oosthuyse et al. 2015a ii [115]	0.6%	-0.31 [-1.24, 0.62]
Oosthuyse et al. 2015b [116]	0.6%	0.15 [-0.83, 1.14]
Rilley et al. 1988 [122]	0.6%	0.16 [-0.77, 1.08]
Robson-Ansley et al. 2009 [124]	0.5%	0.44 [-0.63, 1.50]
Robson-Ansley et al. 2011 [125]	0.6%	0.16 [-0.76, 1.09]
Rollo & Williams 2009 [126]	0.6%	0.28 [-0.71, 1.26]
Rollo & Williams 2010 [127]	0.7%	-0.06 [-0.93, 0.82]
Rollo et al. 2011 [128]	0.7%	0.40 [-0.49, 1.29]
Tsintzas et al. 1995 i [134]	0.5%	0.13 [-0.92, 1.17]
Tsintzas et al. 1996b ii [136]	0.8%	0.37 [-0.48, 1.21]
Wilber et al. 1992 [141]	0.7%	0.85 [-0.08, 1.77]
**Subtotal (95% CI)**	**32.3%**	**0.47 [0.33, 0.62]**
Heterogeneity: $Tau^2 = 0.04$; $Chi^2 = 56.77$, df = 48 (P = 0.18); $I^2 = 15\%$		
Test for overall effect: Z = 6.54 (P < 0.00001)		
**Concentration: 8% < CHO ≤ 10%**		
Cole et al. 1993 ii [64]	0.7%	0.22 [-0.66, 1.10]
Cole et al. 1993 iii [64]	0.7%	0.21 [-0.67, 1.09]
Fulco et al. 2007 i [75]	0.5%	0.58 [-0.48, 1.64]
Fulco et al. 2007 ii [75]	0.5%	0.43 [-0.62, 1.48]
King et al. 2018 iii [90]	0.7%	0.83 [-0.10, 1.75]
King et al. 2019 ii [91]	0.7%	1.09 [0.18, 2.00]
King et al. 2019 iii [91]	0.7%	0.58 [-0.27, 1.44]
Kingwell et al. 1989 [92]	0.6%	0.53 [-0.42, 1.47]
Murray et al. 1989 iii [106]	0.8%	0.05 [-0.75, 0.85]
Nishibata et al. 1993 [114]	0.5%	-0.20 [-1.25, 0.85]
Roberts et al. 2014 i [123]	0.6%	0.50 [-0.47, 1.48]
Roberts et al. 2014 ii [123]	0.6%	-0.06 [-1.02, 0.90]
**Subtotal (95% CI)**	**7.7%**	**0.40 [0.13, 0.67]**
Heterogeneity: $Tau^2 = 0.00$; $Chi^2 = 6.68$, df = 11 (P = 0.82); $I^2 = 0\%$		
Test for overall effect: Z = 2.90 (P = 0.004)		
**Concentration: 10% < CHO ≤ 15%**		
Baur et al. 2014 i [54]	0.5%	0.78 [-0.25, 1.80]
Baur et al. 2014 iii [54]	0.6%	0.23 [-0.75, 1.21]
Currell et al. 2008 i [68]	0.3%	2.55 [1.14, 3.97]
Currell et al. 2008 ii [68]	0.2%	4.37 [2.37, 6.37]
Finger et al. 2018 [74]	0.9%	0.04 [-0.73, 0.81]
Funnell et al. 2017 i [76]	1.0%	-0.28 [-0.97, 0.42]
Funnell et al. 2017 ii [76]	1.0%	-0.23 [-0.92, 0.47]
King et al. 2018 iv [90]	0.7%	0.58 [-0.32, 1.48]
McGawley et al. 2012 [100]	0.7%	0.40 [-0.48, 1.29]
Murray et al. 1991 ii [107]	0.7%	0.40 [-0.49, 1.29]
Naclerio et al. 2014 [108]	0.7%	-0.01 [-0.89, 0.87]
Walton & Rhodes 1997 [139]	0.7%	0.68 [-0.23, 1.59]
**Subtotal (95% CI)**	**8.0%**	**0.53 [0.07, 1.00]**
Heterogeneity: $Tau^2 = 0.44$; $Chi^2 = 33.37$, df = 11 (P = 0.0005); $I^2 = 67\%$		
Test for overall effect: Z = 2.24 (P = 0.02)		
**Concentration: 15% < CHO ≤ 20%**		
Ivy et al. 1983 [84]	0.6%	0.86 [-0.07, 1.78]
Murray et al. 1991 iii [107]	0.7%	0.49 [-0.40, 1.38]
Pettersson et al. 2019 [120]	0.8%	-0.06 [-0.86, 0.74]
Tokmakidis & Karamanolis 2008 [132]	0.8%	0.27 [-0.57, 1.11]
**Subtotal (95% CI)**	**2.9%**	**0.35 [-0.08, 0.78]**
Heterogeneity: $Tau^2 = 0.00$; $Chi^2 = 2.30$, df = 3 (P = 0.51); $I^2 = 0\%$		
Test for overall effect: Z = 1.61 (P = 0.11)		
**Total (95% CI)**	**100.0%**	**0.43 [0.35, 0.51]**
Heterogeneity: $Tau^2 = 0.04$; $Chi^2 = 172.38$, df = 141 (P = 0.04); $I^2 = 18\%$		
Test for overall effect: Z = 10.31 (P < 0.00001)		
Test for subgroup differences: $Chi^2 = 1.26$, df = 6 (P = 0.97), $I^2 = 0\%$		

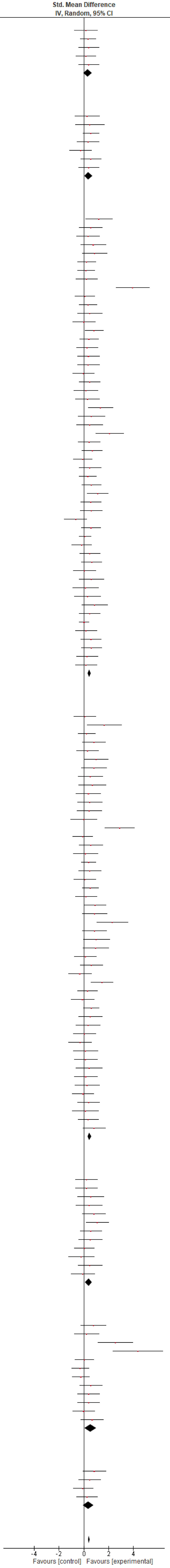

Figure S8: Forest plot shows the effects of experimental carbohydrate supplementation as compared to control on exercise outcome for 142 interventions. Subgroup analyses show the results with regard to supplementation in seven carbohydrate concentration groups (0% < CHO ≤ 2%, 2% < CHO ≤ 4%, 4% < CHO ≤ 6%, 6% < CHO ≤ 8%, 8% < CHO ≤ 10%, 10% < CHO ≤ 15%, 15% < CHO ≤ 20%). The black diamond symbol at the subgroups and at the bottom of the figure represents the standardized mean difference with the 95% confidence intervals for all interventions following random-effect meta-analyses. Studies or trials that provided insufficient data for subgroup classification were not included. Abbreviations: CHO, carbohydrate; CI, confidence interval; IV, inverse variance; SD, standard deviation; Std, standardized; i-vi denote different intervention arms (trials) within the same study

Study or Subgroup	Weight	Std. Mean Difference IV, Random, 95% CI
**CHO dose ≤ 40 g·hr⁻¹**		
Funnell et al. 2017 i [76]	4.5%	-0.28 [-0.97, 0.42]
Funnell et al. 2017 ii [76]	4.5%	-0.23 [-0.92, 0.47]
Goedecke et al. 2013 [79]	6.2%	0.39 [-0.21, 0.99]
Murray et al. 1991 i [107]	2.7%	0.60 [-0.30, 1.50]
Newell et al. 2015 i [111]	5.6%	0.34 [-0.28, 0.97]
Newell et al. 2015 ii [111]	5.5%	0.59 [-0.05, 1.22]
O'Hara et al. 2014 i [118]	2.8%	0.34 [-0.54, 1.22]
O'Hara et al. 2014 ii [118]	2.8%	-0.25 [-1.13, 0.63]
Smith et al. 2010 i [129]	3.4%	0.41 [-0.40, 1.22]
Smith et al. 2010 ii [129]	3.3%	0.49 [-0.32, 1.31]
**Subtotal (95% CI)**	**41.4%**	**0.24 [0.01, 0.48]**
Heterogeneity: Tau² = 0.00; Chi² = 7.71, df = 9 (P = 0.56); I² = 0%		
Test for overall effect: Z = 2.08 (P = 0.04)		
**40 g·hr⁻¹ < CHO dose ≤ 60 g·hr⁻¹**		
Baldassarre et al. 2021 [53]	2.1%	0.83 [-0.21, 1.86]
King et al. 2018 i [90]	2.8%	0.43 [-0.46, 1.32]
Murray et al. 1991 ii [107]	2.8%	0.40 [-0.49, 1.29]
Rilley et al. 1988 [122]	2.6%	0.16 [-0.77, 1.08]
Smith et al. 2010 iii [129]	3.2%	0.65 [-0.18, 1.47]
**Subtotal (95% CI)**	**13.4%**	**0.49 [0.08, 0.89]**
Heterogeneity: Tau² = 0.00; Chi² = 1.10, df = 4 (P = 0.89); I² = 0%		
Test for overall effect: Z = 2.36 (P = 0.02)		
**60 g·hr⁻¹ < CHO dose ≤ 80 g·hr⁻¹**		
King et al. 2018 ii [90]	2.8%	0.19 [-0.69, 1.07]
King et al. 2019 i [91]	2.8%	0.92 [0.03, 1.81]
McConell et al. 1996 [97]	2.0%	1.02 [-0.04, 2.08]
Murray et al. 1991 iii [107]	2.8%	0.49 [-0.40, 1.38]
Newell et al. 2015 iii [111]	5.4%	0.62 [-0.02, 1.25]
Oosthuyse et al. 2015a i [115]	2.6%	0.07 [-0.86, 0.99]
Oosthuyse et al. 2015a ii [115]	2.5%	-0.31 [-1.24, 0.62]
Oosthuyse et al. 2015b [116]	2.3%	0.15 [-0.83, 1.14]
**Subtotal (95% CI)**	**23.2%**	**0.41 [0.10, 0.72]**
Heterogeneity: Tau² = 0.00; Chi² = 6.29, df = 7 (P = 0.51); I² = 0%		
Test for overall effect: Z = 2.62 (P = 0.009)		
**80 g·hr⁻¹ < CHO dose ≤ 100 g·hr⁻¹**		
King et al. 2018 iii [90]	2.6%	0.83 [-0.10, 1.75]
King et al. 2019 ii [91]	2.7%	1.09 [0.18, 2.00]
King et al. 2019 iii [91]	3.0%	0.58 [-0.27, 1.44]
**Subtotal (95% CI)**	**8.2%**	**0.82 [0.31, 1.34]**
Heterogeneity: Tau² = 0.00; Chi² = 0.64, df = 2 (P = 0.73); I² = 0%		
Test for overall effect: Z = 3.13 (P = 0.002)		
**CHO dose > 100 g·hr⁻¹**		
King et al. 2018 iv [90]	2.7%	0.58 [-0.32, 1.48]
Naclerio et al. 2014 [108]	2.9%	-0.01 [-0.89, 0.87]
Pettersson et al. 2019 [120]	3.4%	-0.06 [-0.86, 0.74]
Roberts et al. 2014 i [123]	2.3%	0.50 [-0.47, 1.48]
Roberts et al. 2014 ii [123]	2.4%	-0.06 [-1.02, 0.90]
**Subtotal (95% CI)**	**13.8%**	**0.17 [-0.23, 0.57]**
Heterogeneity: Tau² = 0.00; Chi² = 1.95, df = 4 (P = 0.74); I² = 0%		
Test for overall effect: Z = 0.84 (P = 0.40)		
**Total (95% CI)**	**100.0%**	**0.35 [0.21, 0.50]**
Heterogeneity: Tau² = 0.00; Chi² = 23.08, df = 30 (P = 0.81); I² = 0%		
Test for overall effect: Z = 4.68 (P < 0.00001)		
Test for subgroup differences: Chi² = 5.39, df = 4 (P = 0.25), I² = 25.8%		

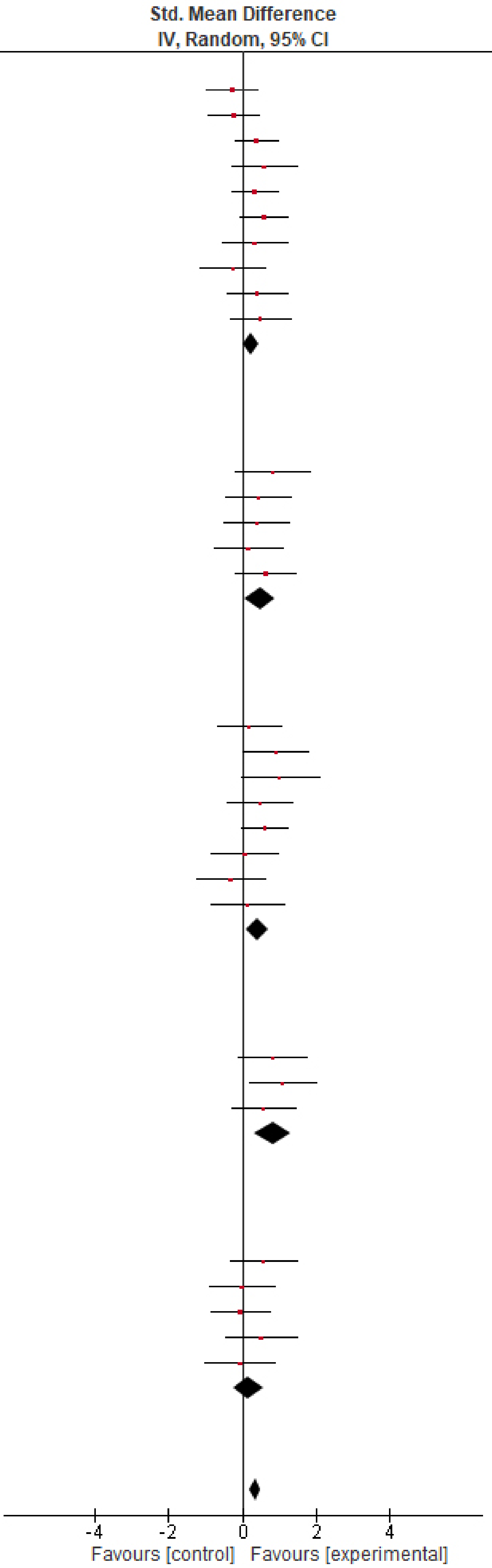

Figure S9: Forest plot shows the effects of experimental carbohydrate supplementation as compared to control on exercise outcome for 31 interventions. Subgroup analyses show the results with regard to supplementation in five carbohydrate dose groups (CHO dose ≤ 40 g·hr⁻¹, 40 g·hr⁻¹ < CHO dose ≤ 60 g·hr⁻¹, 60 g·hr⁻¹ < CHO dose ≤ 80 g·hr⁻¹, 80 g·hr⁻¹ < CHO dose ≤ 100 g·hr⁻¹, CHO dose > 100 g·hr⁻¹). The black diamond symbol at the subgroups and at the bottom of the figure represents the standardized mean difference with the 95% confidence intervals for all interventions following random-effect meta-analyses. Studies or trials that provided insufficient data for subgroup classification were not included. Abbreviations: CHO, carbohydrate; CI, confidence interval; IV, inverse variance; SD, standard deviation; Std, standardized; i-vi denote different intervention arms (trials) within the same study

Study or Subgroup	Weight	Std. Mean Difference IV, Random, 95% CI
**GL-only solution**		
Anastasiou et al. 2004 i [50]	1.8%	0.83 [-0.10, 1.75]
Baur et al. 2014 ii [54]	1.6%	0.55 [-0.46, 1.55]
Baur et al. 2014 iii [54]	1.6%	0.23 [-0.75, 1.21]
Bishop et al. 2001 [56]	1.6%	0.89 [-0.09, 1.87]
Byrne et al. 2005 [58]	2.5%	0.22 [-0.52, 0.97]
Clark et al. 2000 [62]	1.5%	0.00 [-1.05, 1.05]
Currell et al. 2008 i [68]	0.9%	2.55 [1.14, 3.97]
Duhamel et al. 2007 [71]	2.5%	0.85 [0.10, 1.60]
El-Sayed et al. 1995 [72]	1.7%	0.57 [-0.38, 1.52]
El-Sayed et al. 1997 [73]	1.6%	0.16 [-0.82, 1.14]
Hulston & Jeukendrup 2008 [82]	1.9%	0.50 [-0.40, 1.39]
Ivy et al. 1983 [84]	1.8%	0.86 [-0.07, 1.78]
King et al. 2018 i [90]	1.9%	0.43 [-0.46, 1.32]
King et al. 2018 ii [90]	2.0%	0.19 [-0.69, 1.07]
Kingwell et al. 1989 [92]	1.7%	0.53 [-0.42, 1.47]
Maughan et al. 1989 [96]	1.2%	0.51 [-0.65, 1.66]
Murray et al. 1987 i [105]	2.3%	0.63 [-0.16, 1.42]
Murray et al. 1991 i [107]	1.9%	0.60 [-0.30, 1.50]
Newell et al. 2015 i [111]	3.2%	0.34 [-0.28, 0.97]
Newell et al. 2015 ii [111]	3.1%	0.59 [-0.05, 1.22]
Newell et al. 2015 iii [111]	3.1%	0.62 [-0.02, 1.25]
Nishibata et al. 1993 [114]	1.5%	-0.20 [-1.25, 0.85]
O'Hara et al. 2014 ii [118]	1.9%	-0.25 [-1.13, 0.63]
Smith et al. 2010 i [129]	2.2%	0.41 [-0.40, 1.22]
Smith et al. 2010 ii [129]	2.2%	0.49 [-0.32, 1.31]
Smith et al. 2010 iii [129]	2.2%	0.65 [-0.18, 1.47]
Tokmakidis & Karamanolis 2008 [132]	2.1%	0.27 [-0.57, 1.11]
**Subtotal (95% CI)**	**53.5%**	**0.49 [0.32, 0.65]**
Heterogeneity: Tau² = 0.00; Chi² = 18.76, df = 26 (P = 0.85); I² = 0%		
Test for overall effect: Z = 5.78 (P < 0.00001)		
**MD-only solution**		
Alghannam 2011 [48]	0.9%	1.67 [0.28, 3.07]
Carter et al. 2003 i [60]	1.4%	0.70 [-0.39, 1.79]
Carter et al. 2003 ii [60]	1.6%	0.38 [-0.61, 1.37]
Carter et al. 2005 i [61]	1.6%	0.49 [-0.51, 1.49]
Carter et al. 2005 ii [61]	1.6%	0.44 [-0.56, 1.43]
Cole et al. 2018 [65]	1.0%	3.96 [2.62, 5.30]
Finger et al. 2018 [74]	2.4%	0.04 [-0.73, 0.81]
Heesch et al. 2014 i [81]	1.6%	0.28 [-0.71, 1.26]
Heesch et al. 2014 ii [81]	1.6%	0.17 [-0.81, 1.15]
Heesch et al. 2014 iii [81]	1.6%	0.31 [-0.68, 1.30]
Learsi et al. 2019 i [93]	1.6%	0.89 [-0.09, 1.87]
Learsi et al. 2019 ii [93]	1.1%	2.31 [1.05, 3.57]
Naclerio et al. 2014 [108]	2.0%	-0.01 [-0.89, 0.87]
Roberts et al. 2014 ii [123]	1.7%	-0.06 [-1.02, 0.90]
Robson-Ansley et al. 2009 [124]	1.4%	0.44 [-0.63, 1.50]
**Subtotal (95% CI)**	**23.1%**	**0.71 [0.25, 1.16]**
Heterogeneity: Tau² = 0.53; Chi² = 41.46, df = 14 (P = 0.0002); I² = 66%		
Test for overall effect: Z = 3.06 (P = 0.002)		
**SUC-only solution**		
Burgess et al. 1991 [57]	1.8%	0.18 [-0.74, 1.11]
Goedecke et al. 2013 [79]	3.4%	0.39 [-0.21, 0.99]
Gui et al. 2017 [80]	2.1%	0.47 [-0.38, 1.32]
Murray et al. 1989 i [106]	2.2%	0.57 [-0.25, 1.39]
Murray et al. 1989 ii [106]	2.2%	0.30 [-0.50, 1.11]
Murray et al. 1989 iii [106]	2.3%	0.05 [-0.75, 0.85]
Van Essen et al. 2006 [138]	2.0%	0.19 [-0.69, 1.07]
**Subtotal (95% CI)**	**15.8%**	**0.32 [0.02, 0.62]**
Heterogeneity: Tau² = 0.00; Chi² = 1.14, df = 6 (P = 0.98); I² = 0%		
Test for overall effect: Z = 2.10 (P = 0.04)		
**MAL-only solution**		
Anastasiou et al. 2004 ii [50]	1.9%	0.31 [-0.57, 1.20]
Oosthuyse et al. 2015a ii [115]	1.8%	-0.31 [-1.24, 0.62]
**Subtotal (95% CI)**	**3.7%**	**0.02 [-0.62, 0.66]**
Heterogeneity: Tau² = 0.00; Chi² = 0.91, df = 1 (P = 0.34); I² = 0%		
Test for overall effect: Z = 0.05 (P = 0.96)		
**FRU-only solution**		
Cole et al. 1993 ii [64]	1.9%	0.22 [-0.66, 1.10]
**Subtotal (95% CI)**	**1.9%**	**0.22 [-0.66, 1.10]**
Heterogeneity: Not applicable		
Test for overall effect: Z = 0.48 (P = 0.63)		
**GAL-only solution**		
O'Hara et al. 2014 i [118]	1.9%	0.34 [-0.54, 1.22]
**Subtotal (95% CI)**	**1.9%**	**0.34 [-0.54, 1.22]**
Heterogeneity: Not applicable		
Test for overall effect: Z = 0.75 (P = 0.45)		
**Total (95% CI)**	**100.0%**	**0.46 [0.32, 0.60]**
Heterogeneity: Tau² = 0.06; Chi² = 66.20, df = 52 (P = 0.09); I² = 21%		
Test for overall effect: Z = 6.54 (P < 0.00001)		
Test for subgroup differences: Chi² = 4.23, df = 5 (P = 0.52), I² = 0%		

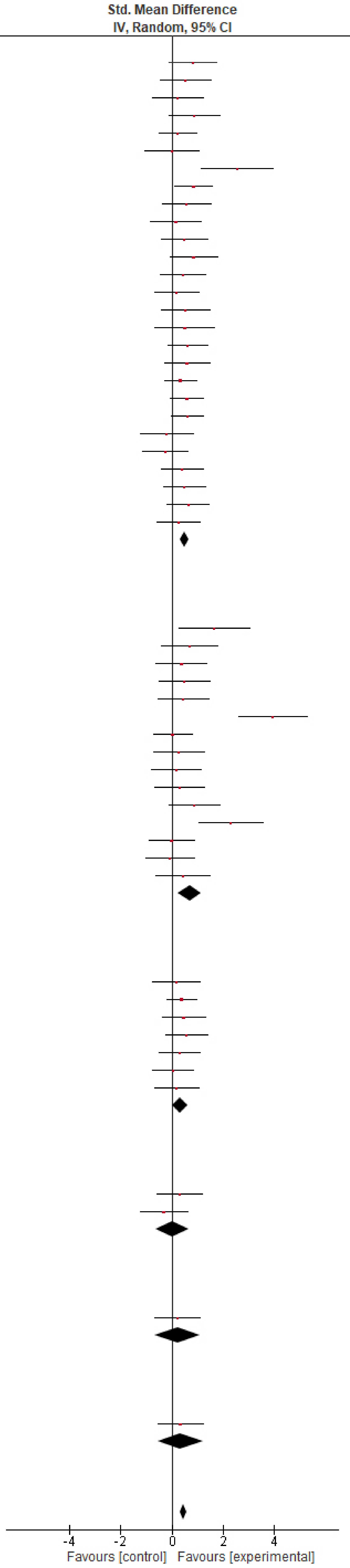

Figure S10: Forest plot shows the effects of experimental carbohydrate supplementation as compared to control on exercise outcome for 53 interventions. Subgroup analyses show the results with regard to supplementation in six single source carbohydrate only groups (GL, MD, SUC, MAL, FRU, GAL). The black diamond symbol at the subgroups and at the bottom of the figure represents the standardized mean difference with the 95% confidence intervals for all interventions following random-effect meta-analyses. Studies or trials that provided insufficient data for subgroup classification were not included. Abbreviations: CHO, carbohydrate; CI, confidence in-terval; FRU, fructose; GAL, galactose; GL, glucose; IV, inverse variance; MD, maltodextrin; MAL, maltose; SD, standard deviation; Std, standardized; SUC, sucrose; i-vi denote different intervention arms (trials) within the same study

Study or Subgroup	Weight	Std. Mean Difference IV, Random, 95% CI
**Single-source CHO solution**		
Alghannam 2011 [48]	0.4%	1.67 [0.28, 3.07]
Anastasiou et al. 2004 i [50]	0.9%	0.83 [-0.10, 1.75]
Anastasiou et al. 2004 ii [50]	1.0%	0.31 [-0.57, 1.20]
Baur et al. 2014 ii [54]	0.8%	0.55 [-0.46, 1.55]
Baur et al. 2014 iii [54]	0.8%	0.23 [-0.75, 1.21]
Bishop et al. 2001 [56]	0.8%	0.89 [-0.09, 1.87]
Burgess et al. 1991 [57]	0.9%	0.18 [-0.74, 1.11]
Byrne et al. 2005 [58]	1.3%	0.22 [-0.52, 0.97]
Carter et al. 2003 i [60]	0.7%	0.70 [-0.39, 1.79]
Carter et al. 2003 ii [60]	0.8%	0.38 [-0.61, 1.37]
Carter et al. 2005 i [61]	0.8%	0.49 [-0.51, 1.49]
Carter et al. 2005 ii [61]	0.8%	0.44 [-0.56, 1.43]
Clark et al. 2000 [62]	0.7%	0.00 [-1.05, 1.05]
Cole et al. 1993 ii [64]	1.0%	0.22 [-0.66, 1.10]
Cole et al. 2018 [65]	0.5%	3.96 [2.62, 5.30]
Currell et al. 2008 i [68]	0.4%	2.55 [1.14, 3.97]
Duhamel et al. 2007 [71]	1.3%	0.85 [0.10, 1.60]
El-Sayed et al. 1995 [72]	0.9%	0.57 [-0.38, 1.52]
El-Sayed et al. 1997 [73]	0.8%	0.16 [-0.82, 1.14]
Finger et al. 2018 [74]	1.3%	0.04 [-0.73, 0.81]
Goedecke et al. 2013 [79]	1.8%	0.39 [-0.21, 0.99]
Gui et al. 2017 [80]	1.1%	0.47 [-0.38, 1.32]
Heesch et al. 2014 i [81]	0.8%	0.28 [-0.71, 1.26]
Heesch et al. 2014 ii [81]	0.8%	0.17 [-0.81, 1.15]
Heesch et al. 2014 iii [81]	0.8%	0.31 [-0.68, 1.30]
Hulston & Jeukendrup 2008 [82]	1.0%	0.50 [-0.40, 1.39]
Ivy et al. 1983 [84]	0.9%	0.86 [-0.07, 1.78]
King et al. 2018 i [90]	1.0%	0.43 [-0.46, 1.32]
King et al. 2018 ii [90]	1.0%	0.19 [-0.69, 1.07]
Kingwell et al. 1989 [92]	0.9%	0.53 [-0.42, 1.47]
Learsi et al. 2019 i [93]	0.8%	0.89 [-0.09, 1.87]
Learsi et al. 2019 ii [93]	0.5%	2.31 [1.05, 3.57]
Maughan et al. 1989 [96]	0.6%	0.51 [-0.65, 1.66]
Murray et al. 1987 i [105]	1.2%	0.63 [-0.16, 1.42]
Murray et al. 1989 i [106]	1.1%	0.57 [-0.25, 1.39]
Murray et al. 1989 ii [106]	1.2%	0.30 [-0.50, 1.11]
Murray et al. 1989 iii [106]	1.2%	0.05 [-0.75, 0.85]
Murray et al. 1991 i [107]	1.0%	0.60 [-0.30, 1.50]
Naclerio et al. 2014 [108]	1.0%	-0.01 [-0.89, 0.87]
Newell et al. 2015 i [111]	1.7%	0.34 [-0.28, 0.97]
Newell et al. 2015 ii [111]	1.7%	0.59 [-0.05, 1.22]
Newell et al. 2015 iii [111]	1.7%	0.62 [-0.02, 1.25]
Nishibata et al. 1993 [114]	0.7%	-0.20 [-1.25, 0.85]
Oosthuyse et al. 2015a ii [115]	0.9%	-0.31 [-1.24, 0.62]
O'Hara et al. 2014 i [118]	1.0%	0.34 [-0.54, 1.22]
O'Hara et al. 2014 ii [118]	1.0%	-0.25 [-1.13, 0.63]
Roberts et al. 2014 ii [123]	0.9%	-0.06 [-1.02, 0.90]
Robson-Ansley et al. 2009 [124]	0.7%	0.44 [-0.63, 1.50]
Smith et al. 2010 i [129]	1.2%	0.41 [-0.40, 1.22]
Smith et al. 2010 ii [129]	1.1%	0.49 [-0.32, 1.31]
Smith et al. 2010 iii [129]	1.1%	0.65 [-0.18, 1.47]
Tokmakidis & Karamanolis 2008 [132]	1.1%	0.27 [-0.57, 1.11]
Van Essen et al. 2006 [138]	1.0%	0.19 [-0.69, 1.07]
**Subtotal (95% CI)**	**51.6%**	**0.46 [0.32, 0.60]**
Heterogeneity: Tau² = 0.06; Chi² = 66.20, df = 52 (P = 0.09); I² = 21%		
Test for overall effect: Z = 6.54 (P < 0.00001)		
**Double-source CHO solution**		
Anastasiou et al. 2004 iii [50]	0.9%	1.01 [0.07, 1.96]
Baldassarre et al. 2021 [53]	0.8%	0.83 [-0.21, 1.86]
Baur et al. 2014 i [54]	0.8%	0.78 [-0.25, 1.80]
Cole et al. 1993 i [64]	1.0%	0.23 [-0.65, 1.11]
Cole et al. 1993 iii [64]	1.0%	0.21 [-0.67, 1.09]
Currell et al. 2008 ii [68]	0.2%	4.37 [2.37, 6.37]
Hulston & Jeukendrup 2009 [83]	0.8%	1.35 [0.36, 2.35]
Jarvis et al. 1999 [85]	1.0%	0.08 [-0.79, 0.96]
Kang et al. 1995 [87]	0.7%	0.61 [-0.47, 1.69]
Kang et al. 1996 [88]	0.7%	0.48 [-0.59, 1.55]
King et al. 2018 iii [90]	0.9%	0.83 [-0.10, 1.75]
King et al. 2018 iv [90]	1.0%	0.58 [-0.32, 1.48]
King et al. 2019 i [91]	1.0%	0.92 [0.03, 1.81]
King et al. 2019 ii [91]	0.9%	1.09 [0.18, 2.00]
King et al. 2019 iii [91]	1.0%	0.58 [-0.27, 1.44]
McGawley et al. 2012 [100]	1.0%	0.40 [-0.48, 1.29]
Millard-Stafford et al. 1990 [101]	1.0%	0.14 [-0.74, 1.02]
Millard-Stafford et al. 2005 i [102]	1.0%	0.50 [-0.39, 1.40]
Murray et al. 1987 ii [105]	1.1%	1.13 [0.29, 1.97]
Murray et al. 1987 iii [105]	1.0%	1.49 [0.60, 2.37]
Murray et al. 1991 ii [107]	1.0%	0.40 [-0.49, 1.29]
Murray et al. 1991 iii [107]	1.0%	0.49 [-0.40, 1.38]
Oosthuyse et al. 2015a i [115]	0.9%	0.07 [-0.86, 0.99]
Oosthuyse et al. 2015b [116]	0.8%	0.15 [-0.83, 1.14]
Pettersson et al. 2019 [120]	1.2%	-0.06 [-0.86, 0.74]
Pottier et al. 2010 [121]	1.2%	-0.16 [-0.96, 0.64]
Rilley et al. 1988 [122]	0.9%	0.16 [-0.77, 1.08]
Roberts et al. 2014 i [123]	0.8%	0.50 [-0.47, 1.48]
Tsintzas et al. 1995 i [134]	0.7%	0.13 [-0.92, 1.17]
Wilber et al. 1992 [141]	0.9%	0.85 [-0.08, 1.77]
**Subtotal (95% CI)**	**27.3%**	**0.57 [0.37, 0.76]**
Heterogeneity: Tau² = 0.07; Chi² = 38.35, df = 29 (P = 0.11); I² = 24%		
Test for overall effect: Z = 5.69 (P < 0.00001)		
**Triple-or-more-source CHO solution**		
Campbell et al. 2008 [59]	1.5%	0.19 [-0.50, 0.89]
Fulco et al. 2007 i [75]	0.7%	0.58 [-0.48, 1.64]
Fulco et al. 2007 ii [75]	0.7%	0.43 [-0.62, 1.48]
Funnell et al. 2017 i [76]	1.5%	-0.28 [-0.97, 0.42]
Funnell et al. 2017 ii [76]	1.5%	-0.23 [-0.92, 0.47]
Millard-Stafford et al. 2005 ii [102]	1.0%	0.62 [-0.29, 1.52]
Morris et al. 2003 [104]	0.9%	-0.31 [-1.24, 0.62]
Osterberg et al. 2008 [117]	1.2%	0.60 [-0.19, 1.39]
Tsintzas et al. 1993 [133]	0.7%	0.14 [-0.91, 1.19]
Tsintzas et al. 1995 ii [134]	0.7%	0.31 [-0.75, 1.36]
Tsintzas et al. 1996a [135]	0.7%	0.89 [-0.16, 1.93]
Tsintzas et al. 1996b i [136]	1.1%	0.49 [-0.36, 1.34]
Watson et al. 2012 i [140]	1.2%	0.19 [-0.61, 0.99]
Watson et al. 2012 ii [140]	1.1%	0.59 [-0.23, 1.41]
Watson et al. 2012 iii [140]	1.1%	0.60 [-0.22, 1.42]
Watson et al. 2012 iv [140]	1.1%	0.41 [-0.40, 1.22]
Watson et al. 2012 v [140]	1.1%	0.41 [-0.40, 1.22]
Watson et al. 2012 vi [140]	1.1%	0.63 [-0.19, 1.46]
Williams et al. 1990 i [142]	1.0%	0.27 [-0.60, 1.14]
Williams et al. 1990 ii [142]	1.0%	0.18 [-0.68, 1.05]
**Subtotal (95% CI)**	**21.1%**	**0.30 [0.11, 0.49]**
Heterogeneity: Tau² = 0.00; Chi² = 11.36, df = 19 (P = 0.91); I² = 0%		
Test for overall effect: Z = 3.11 (P = 0.002)		
**Total (95% CI)**	**100.0%**	**0.46 [0.36, 0.55]**
Heterogeneity: Tau² = 0.04; Chi² = 119.81, df = 102 (P = 0.11); I² = 15%		
Test for overall effect: Z = 9.35 (P < 0.00001)		
Test for subgroup differences: Chi² = 3.79, df = 2 (P = 0.15), I² = 47.3%		

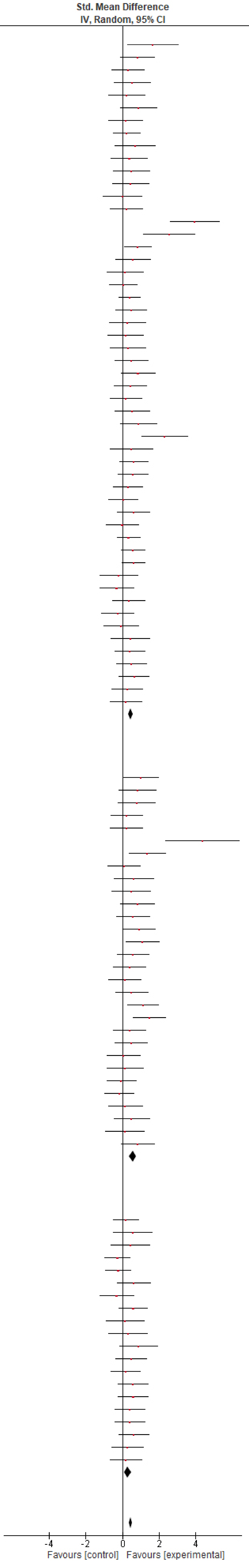

Figure S11: Forest plot shows the effects of experimental carbohydrate supplementation as compared to control on exercise outcome for 103 interventions. Subgroup analyses show the results with regard to supplementation in three carbohydrate solution formulation groups (Single-source CHO solution, Double-source CHO solution, Triple-or-more-source CHO solution). The black diamond symbol at the subgroups and at the bottom of the figure represents the standardized mean difference with the 95% confidence intervals for all interventions following random-effect meta-analyses. Studies or trials that provided insufficient data for subgroup classification were not included. Abbreviations: CHO, carbohydrate; CI, confidence interval; IV, inverse variance; SD, standard deviation; Std, standardized; i-vi denote different intervention arms (tri-als) within the same study

Study or Subgroup	Weight	Std. Mean Difference IV, Random, 95% CI
**Administration time of CHO supplementation: Prior to or at the beginning of exercise**		
El-Sayed et al. 1997 [73]	0.6%	0.16 [-0.82, 1.14]
Funnell et al. 2017 i [76]	1.0%	-0.28 [-0.97, 0.42]
Heesch et al. 2014 ii [81]	0.6%	0.17 [-0.81, 1.15]
O'Hara et al. 2014 i [118]	0.7%	0.34 [-0.54, 1.22]
O'Hara et al. 2014 ii [118]	0.7%	-0.25 [-1.13, 0.63]
Tokmakidis & Karamanolis 2008 [132]	0.8%	0.27 [-0.57, 1.11]
Walton & Rhodes 1997 [139]	0.7%	0.68 [-0.23, 1.59]
**Subtotal (95% CI)**	**5.0%**	**0.12 [-0.21, 0.44]**
Heterogeneity: Tau² = 0.00; Chi² = 3.74, df = 6 (P = 0.71); I² = 0%		
Test for overall effect: Z = 0.70 (P = 0.48)		
**Administration time of CHO supplementation: During exercise**		
Ali et al. 2007 [49]	1.0%	0.22 [-0.47, 0.92]
Anastasiou et al. 2004 i [50]	0.7%	0.83 [-0.10, 1.75]
Anastasiou et al. 2004 ii [50]	0.7%	0.31 [-0.57, 1.20]
Anastasiou et al. 2004 iii [50]	0.6%	1.01 [0.07, 1.96]
Angus et al. 2000 [51]	0.5%	1.23 [0.13, 2.33]
Bailey et al. 2000 i [52]	0.6%	0.57 [-0.38, 1.52]
Bailey et al. 2000 ii [52]	0.6%	0.34 [-0.59, 1.27]
Baldassarre et al. 2021 [53]	0.5%	0.83 [-0.21, 1.86]
Below et al. 1995 [55]	0.5%	0.77 [-0.26, 1.80]
Bishop et al. 2001 [56]	0.6%	0.89 [-0.09, 1.87]
Burgess et al. 1991 [57]	0.6%	0.18 [-0.74, 1.11]
Byrne et al. 2005 [58]	0.9%	0.22 [-0.52, 0.97]
Campbell et al. 2008 [59]	1.0%	0.19 [-0.50, 0.89]
Carter et al. 2003 i [60]	0.5%	0.70 [-0.39, 1.79]
Carter et al. 2003 ii [60]	0.6%	0.38 [-0.61, 1.37]
Carter et al. 2005 i [61]	0.6%	0.49 [-0.51, 1.49]
Carter et al. 2005 ii [61]	0.6%	0.44 [-0.56, 1.43]
Clark et al. 2000 [62]	0.5%	0.00 [-1.05, 1.05]
Clarke et al. 2011 [63]	0.4%	2.90 [1.70, 4.10]
Cole et al. 1993 i [64]	0.7%	0.23 [-0.65, 1.11]
Cole et al. 1993 ii [64]	0.7%	0.22 [-0.66, 1.10]
Cole et al. 1993 iii [64]	0.7%	0.21 [-0.67, 1.09]
Cole et al. 2018 [65]	0.3%	3.96 [2.62, 5.30]
Coletta et al. 2013 i [66]	0.8%	0.08 [-0.72, 0.88]
Coletta et al. 2013 ii [66]	0.8%	-0.07 [-0.87, 0.73]
Cureton et al. 2007 [67]	1.0%	0.35 [-0.35, 1.05]
Currell et al. 2008 i [68]	0.3%	2.55 [1.14, 3.97]
Currell et al. 2008 ii [68]	0.2%	4.37 [2.37, 6.37]
Davis et al. 2000 [69]	0.6%	0.49 [-0.51, 1.49]
Desbrow et al. 2004 [70]	0.7%	0.02 [-0.90, 0.95]
Duhamel et al. 2007 [71]	0.9%	0.85 [0.10, 1.60]
El-Sayed et al. 1995 [72]	0.6%	0.57 [-0.38, 1.52]
Finger et al. 2018 [74]	0.9%	0.04 [-0.73, 0.81]
Fulco et al. 2007 i [75]	0.5%	0.58 [-0.48, 1.64]
Fulco et al. 2007 ii [75]	0.5%	0.43 [-0.62, 1.48]
Glace et al. 2019 i [78]	0.7%	0.28 [-0.60, 1.16]
Glace et al. 2019 ii [78]	0.7%	0.38 [-0.51, 1.26]
Glace et al. 2019 iii [78]	0.7%	0.38 [-0.51, 1.26]
Glace et al. 2019 iv [78]	0.7%	-0.02 [-0.89, 0.86]
Gui et al. 2017 [80]	0.7%	0.47 [-0.38, 1.32]
Heesch et al. 2014 i [81]	0.6%	0.28 [-0.71, 1.26]
Hulston & Jeukendrup 2008 [82]	0.7%	0.50 [-0.40, 1.39]
Hulston & Jeukendrup 2009 [83]	0.6%	1.35 [0.36, 2.35]
Ivy et al. 1983 [84]	0.6%	0.86 [-0.07, 1.78]
Jarvis et al. 1999 [85]	0.7%	0.08 [-0.79, 0.96]
Jeukendrup et al. 1997 [86]	1.1%	0.54 [-0.11, 1.19]
Kang et al. 1995 [87]	0.5%	0.61 [-0.47, 1.69]
Kang et al. 1996 [88]	0.5%	0.48 [-0.59, 1.55]
Khanna & Manna 2005 [89]	0.5%	2.10 [0.96, 3.24]
King et al. 2018 i [90]	0.7%	0.43 [-0.46, 1.32]
King et al. 2018 ii [90]	0.7%	0.19 [-0.69, 1.07]
King et al. 2018 iii [90]	0.7%	0.83 [-0.10, 1.75]
King et al. 2018 iv [90]	0.7%	0.58 [-0.32, 1.48]
King et al. 2019 i [91]	0.7%	0.92 [0.03, 1.81]
King et al. 2019 ii [91]	0.7%	1.09 [0.18, 2.00]
King et al. 2019 iii [91]	0.7%	0.58 [-0.27, 1.44]
Kingwell et al. 1989 [92]	0.6%	0.53 [-0.42, 1.47]
Lugo et al. 1993 [94]	0.6%	0.88 [-0.10, 1.86]
Martínez-Lagunas et al. 2010 [95]	0.8%	0.69 [-0.14, 1.52]
Maughan et al. 1989 [96]	0.4%	0.51 [-0.65, 1.66]
McConell et al. 1996 [97]	0.5%	1.02 [-0.04, 2.08]
McConell et al. 1999 [98]	0.5%	0.97 [-0.08, 2.03]
McConell et al. 2000 [99]	0.9%	-0.08 [-0.85, 0.69]
McGawley et al. 2012 [100]	0.7%	0.40 [-0.48, 1.29]
Millard-Stafford et al. 1990 [101]	0.7%	0.14 [-0.74, 1.02]
Millard-Stafford et al. 2005 i [102]	0.7%	0.50 [-0.39, 1.40]
Millard-Stafford et al. 2005 ii [102]	0.7%	0.62 [-0.29, 1.52]
Millard-Stafford et al. 2007 [103]	1.0%	0.34 [-0.36, 1.04]
Morris et al. 2003 [104]	0.6%	-0.31 [-1.24, 0.62]
Murray et al. 1987 i [105]	0.8%	0.63 [-0.16, 1.42]
Murray et al. 1987 ii [105]	0.8%	1.13 [0.29, 1.97]
Murray et al. 1987 iii [105]	0.7%	1.49 [0.60, 2.37]
Murray et al. 1989 i [106]	0.8%	0.57 [-0.25, 1.39]
Murray et al. 1989 ii [106]	0.8%	0.30 [-0.50, 1.11]
Murray et al. 1989 iii [106]	0.8%	0.05 [-0.75, 0.85]
Murray et al. 1991 i [107]	0.7%	0.60 [-0.30, 1.50]
Murray et al. 1991 ii [107]	0.7%	0.40 [-0.49, 1.29]
Murray et al. 1991 iii [107]	0.7%	0.49 [-0.40, 1.38]
Nassif et al. 2014 [109]	0.7%	-0.66 [-1.56, 0.25]
Nassis et al. 1998 [110]	0.7%	-0.10 [-1.02, 0.83]
Nicholas et al. 1995 [112]	0.6%	0.55 [-0.40, 1.49]
Nikolopoulos et al. 2004 [113]	0.6%	0.34 [-0.65, 1.33]
Nishibata et al. 1993 [114]	0.5%	-0.20 [-1.25, 0.85]
Oosthuyse et al. 2015a i [115]	0.7%	0.07 [-0.86, 0.99]
Oosthuyse et al. 2015a ii [115]	0.6%	-0.31 [-1.24, 0.62]
Osterberg et al. 2008 [117]	0.8%	0.60 [-0.19, 1.39]
O'Neal et al. 2013 [119]	1.7%	0.10 [-0.36, 0.56]
Rilley et al. 1988 [122]	0.6%	0.16 [-0.77, 1.08]
Robson-Ansley et al. 2009 [124]	0.5%	0.44 [-0.63, 1.50]
Smith et al. 2010 i [129]	0.8%	0.41 [-0.40, 1.22]
Smith et al. 2010 ii [129]	0.8%	0.49 [-0.32, 1.31]
Smith et al. 2010 iii [129]	0.8%	0.65 [-0.18, 1.47]
Steiner et al. 2009 [130]	0.7%	0.07 [-0.85, 1.00]
Sun et al. 2015 [131]	0.6%	0.62 [-0.39, 1.63]
Tsintzas et al. 1993 [133]	0.5%	0.14 [-0.91, 1.19]
Tsintzas et al. 1995 i [134]	0.5%	0.13 [-0.92, 1.17]
Tsintzas et al. 1995 ii [134]	0.5%	0.31 [-0.75, 1.36]
Tsintzas et al. 1996a [135]	0.5%	0.89 [-0.16, 1.93]
Tsintzas et al. 1996b i [136]	0.7%	0.49 [-0.36, 1.34]
Tsintzas et al. 1996b ii [136]	0.8%	0.37 [-0.48, 1.21]
Utter et al. 2002 [137]	2.1%	0.04 [-0.36, 0.43]
Van Essen et al. 2006 [138]	0.7%	0.19 [-0.69, 1.07]
Wilber et al. 1992 [141]	0.7%	0.85 [-0.08, 1.77]
Williams et al. 1990 i [142]	0.7%	0.27 [-0.60, 1.14]
Williams et al. 1990 ii [142]	0.7%	0.18 [-0.68, 1.05]
**Subtotal (95% CI)**	**72.4%**	**0.47 [0.37, 0.58]**
Heterogeneity: Tau² = 0.07; Chi² = 139.00, df = 104 (P = 0.01); I² = 25%		
Test for overall effect: Z = 9.23 (P < 0.00001)		
**Administration time of CHO supplementation: Late in exercise**		
Heesch et al. 2014 iii [81]	0.6%	0.31 [-0.68, 1.30]
**Subtotal (95% CI)**	**0.6%**	**0.31 [-0.68, 1.30]**
Heterogeneity: Not applicable		
Test for overall effect: Z = 0.61 (P = 0.54)		
**Administration time of CHO supplementation: Prior to or at the beginning & during exercise**		
Acker-Hewitt et al. 2012 [47]	0.7%	0.09 [-0.79, 0.97]
Alghannam 2011 [48]	0.3%	1.67 [0.28, 3.07]
Baur et al. 2014 i [54]	0.5%	0.78 [-0.25, 1.80]
Baur et al. 2014 ii [54]	0.6%	0.55 [-0.46, 1.55]
Baur et al. 2014 iii [54]	0.6%	0.23 [-0.75, 1.21]
Funnell et al. 2017 ii [76]	1.0%	-0.23 [-0.92, 0.47]
Ganio et al. 2010 [77]	0.9%	0.42 [-0.33, 1.17]
Goedecke et al. 2013 [79]	1.3%	0.39 [-0.21, 0.99]
Learsi et al. 2019 i [93]	0.6%	0.89 [-0.09, 1.87]
Learsi et al. 2019 ii [93]	0.4%	2.31 [1.05, 3.57]
Naclerio et al. 2014 [108]	0.7%	-0.01 [-0.89, 0.87]
Newell et al. 2015 i [111]	1.2%	0.34 [-0.28, 0.97]
Newell et al. 2015 ii [111]	1.2%	0.59 [-0.05, 1.22]
Newell et al. 2015 iii [111]	1.2%	0.62 [-0.02, 1.25]
Oosthuyse et al. 2015b [116]	0.6%	0.15 [-0.83, 1.14]
Pettersson et al. 2019 [120]	0.8%	-0.06 [-0.86, 0.74]
Pottier et al. 2010 [121]	0.8%	-0.16 [-0.96, 0.64]
Roberts et al. 2014 i [123]	0.6%	0.50 [-0.47, 1.48]
Roberts et al. 2014 ii [123]	0.6%	-0.06 [-1.02, 0.90]
Robson-Ansley et al. 2011 [125]	0.6%	0.16 [-0.76, 1.09]
Rollo & Williams 2009 [126]	0.6%	0.28 [-0.71, 1.26]
Rollo & Williams 2010 [127]	0.7%	-0.06 [-0.93, 0.82]
Rollo et al. 2011 [128]	0.7%	0.40 [-0.49, 1.29]
Watson et al. 2012 i [140]	0.8%	0.19 [-0.61, 0.99]
Watson et al. 2012 ii [140]	0.8%	0.59 [-0.23, 1.41]
Watson et al. 2012 iii [140]	0.8%	0.60 [-0.22, 1.42]
Watson et al. 2012 iv [140]	0.8%	0.41 [-0.40, 1.22]
Watson et al. 2012 v [140]	0.8%	0.41 [-0.40, 1.22]
Watson et al. 2012 vi [140]	0.8%	0.63 [-0.19, 1.46]
**Subtotal (95% CI)**	**22.0%**	**0.37 [0.22, 0.52]**
Heterogeneity: Tau² = 0.00; Chi² = 25.55, df = 28 (P = 0.60); I² = 0%		
Test for overall effect: Z = 4.70 (P < 0.00001)		
**Total (95% CI)**	**100.0%**	**0.43 [0.35, 0.51]**
Heterogeneity: Tau² = 0.04; Chi² = 172.38, df = 141 (P = 0.04); I² = 18%		
Test for overall effect: Z = 10.31 (P < 0.00001)		
Test for subgroup differences: Chi² = 4.85, df = 3 (P = 0.18), I² = 38.2%		

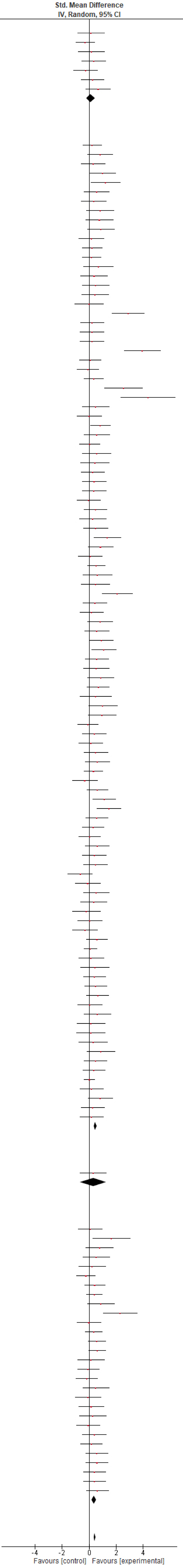

Figure S12: Forest plot shows the effects of experimental carbohydrate supplementation as compared to control on exercise outcome for 142 interventions. Subgroup analyses show the results with regard to supplementation in four carbohydrate administration time groups (Prior to or at the beginning, During, Prior to or at the beginning + during, Late in exercise). The black diamond symbol at the subgroups and at the bottom of the figure represents the standardized mean difference with the 95% confidence intervals for all interventions following random-effect meta-analyses. Studies or trials that provided insufficient data for subgroup classification were not included. Abbreviations: CHO, carbohydrate; CI, confidence interval; IV, inverse variance; SD, standard deviation; Std, standardized; i-vi denote different intervention arms (trials) within the same study

Study or Subgroup	Weight	Std. Mean Difference IV, Random, 95% CI
**Temperature of CHO supplement administration: Cool (<18°C)**		
Ivy et al. 1983 [84]	3.3%	0.86 [-0.07, 1.78]
Murray et al. 1989 i [106]	4.2%	0.57 [-0.25, 1.39]
Murray et al. 1989 ii [106]	4.3%	0.30 [-0.50, 1.11]
Murray et al. 1989 iii [106]	4.4%	0.05 [-0.75, 0.85]
Murray et al. 1991 i [107]	3.4%	0.60 [-0.30, 1.50]
Murray et al. 1991 ii [107]	3.5%	0.40 [-0.49, 1.29]
Murray et al. 1991 iii [107]	3.5%	0.49 [-0.40, 1.38]
Nassif et al. 2014 [109]	3.4%	-0.66 [-1.56, 0.25]
Nassis et al. 1998 [110]	3.3%	-0.10 [-1.02, 0.83]
Newell et al. 2015 i [111]	7.2%	0.34 [-0.28, 0.97]
Newell et al. 2015 ii [111]	6.9%	0.59 [-0.05, 1.22]
Newell et al. 2015 iii [111]	6.9%	0.62 [-0.02, 1.25]
Nishibata et al. 1993 [114]	2.5%	-0.20 [-1.25, 0.85]
O'Neal et al. 2013 [119]	13.1%	0.10 [-0.36, 0.56]
**Subtotal (95% CI)**	**69.8%**	**0.30 [0.10, 0.50]**
Heterogeneity: $Tau^2 = 0.00$; $Chi^2 = 11.17$, df = 13 (P = 0.60); $I^2 = 0\%$		
Test for overall effect: Z = 2.94 (P = 0.003)		
**Temperature of CHO supplement administration: Neutral (18-26°C)**		
Duhamel et al. 2007 [71]	4.9%	0.85 [0.10, 1.60]
Watson et al. 2012 i [140]	4.3%	0.19 [-0.61, 0.99]
Watson et al. 2012 ii [140]	4.1%	0.59 [-0.23, 1.41]
Watson et al. 2012 iii [140]	4.1%	0.60 [-0.22, 1.42]
Watson et al. 2012 iv [140]	4.3%	0.41 [-0.40, 1.22]
Watson et al. 2012 v [140]	4.3%	0.41 [-0.40, 1.22]
Watson et al. 2012 vi [140]	4.1%	0.63 [-0.19, 1.46]
**Subtotal (95% CI)**	**30.2%**	**0.53 [0.23, 0.84]**
Heterogeneity: $Tau^2 = 0.00$; $Chi^2 = 1.65$, df = 6 (P = 0.95); $I^2 = 0\%$		
Test for overall effect: Z = 3.43 (P = 0.0006)		
**Total (95% CI)**	**100.0%**	**0.37 [0.20, 0.54]**
Heterogeneity: $Tau^2 = 0.00$; $Chi^2 = 14.37$, df = 20 (P = 0.81); $I^2 = 0\%$		
Test for overall effect: Z = 4.34 (P < 0.0001)		
Test for subgroup differences: $Chi^2 = 1.56$, df = 1 (P = 0.21), $I^2 = 35.7\%$		

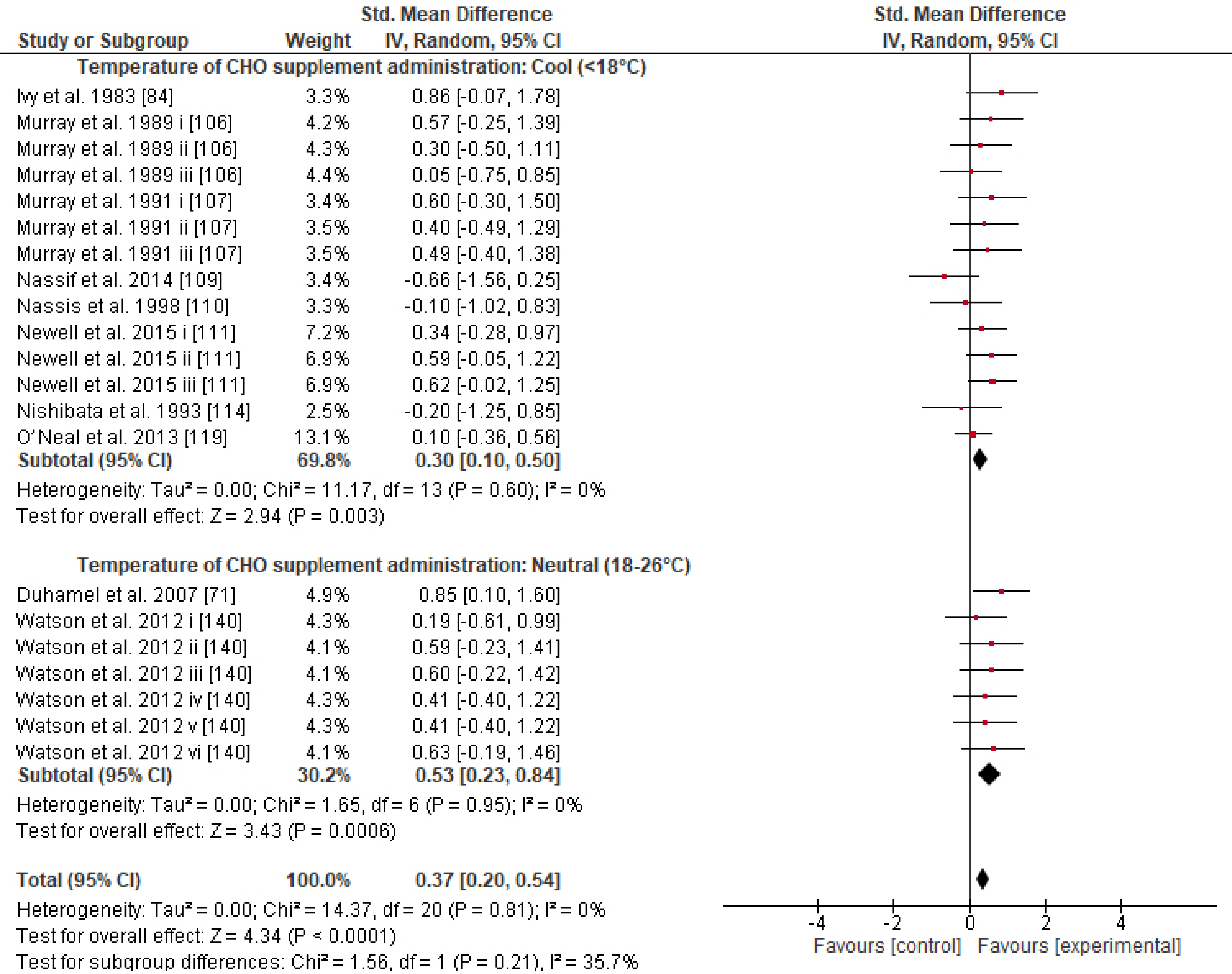

Figure S13: Forest plot shows the effects of experimental carbohydrate supplementation as compared to control on exercise outcome for 21 interventions. Subgroup analyses show the results with regard to supplementation in two temperature of supplement administration groups [Cool (< 18°C), Neutral (18-26°C)]. The black diamond symbol at the subgroups and at the bottom of the figure represents the standardized mean difference with the 95% confidence intervals for all interventions following random-effect meta-analyses. Studies or trials that provided insufficient data for subgroup classification were not included. Abbreviations: CHO, carbohydrate; CI, confidence interval; IV, inverse variance; SD, standard deviation; Std, standardized; i-vi denote different intervention arms (trials) within the same study

Study or Subgroup	Weight	Std. Mean Difference IV, Fixed, 95% CI
**GL:FRU solution**		
Baldassarre et al. 2021 [53]	1.5%	0.83 [-0.21, 1.86]
Baur et al. 2014 i [54]	1.5%	0.78 [-0.25, 1.80]
Cole et al. 1993 iii [64]	2.1%	0.21 [-0.67, 1.09]
Currell et al. 2008 ii [68]	0.4%	4.37 [2.37, 6.37]
Hulston & Jeukendrup 2009 [83]	1.6%	1.35 [0.36, 2.35]
King et al. 2018 iii [90]	1.9%	0.83 [-0.10, 1.75]
King et al. 2018 iv [90]	2.0%	0.58 [-0.32, 1.48]
King et al. 2019 i [91]	2.0%	0.92 [0.03, 1.81]
King et al. 2019 ii [91]	1.9%	1.09 [0.18, 2.00]
King et al. 2019 iii [91]	2.2%	0.58 [-0.27, 1.44]
Millard-Stafford et al. 1990 [101]	2.1%	0.14 [-0.74, 1.02]
Murray et al. 1987 iii [105]	2.0%	1.49 [0.60, 2.37]
Rilley et al. 1988 [122]	1.9%	0.16 [-0.77, 1.08]
**Subtotal (95% CI)**	**23.1%**	**0.80 [0.53, 1.06]**
Heterogeneity: Chi² = 22.45, df = 12 (P = 0.03); I² = 47%		
Test for overall effect: Z = 5.92 (P < 0.00001)		
**GL:SUC solution**		
Cole et al. 1993 i [64]	2.1%	0.23 [-0.65, 1.11]
Kang et al. 1995 [87]	1.4%	0.61 [-0.47, 1.69]
Kang et al. 1996 [88]	1.4%	0.48 [-0.59, 1.55]
Millard-Stafford et al. 2005 i [102]	2.0%	0.50 [-0.39, 1.40]
Murray et al. 1987 ii [105]	2.3%	1.13 [0.29, 1.97]
Pottier et al. 2010 [121]	2.5%	-0.16 [-0.96, 0.64]
Wilber et al. 1992 [141]	1.9%	0.85 [-0.08, 1.77]
**Subtotal (95% CI)**	**13.5%**	**0.50 [0.16, 0.85]**
Heterogeneity: Chi² = 5.72, df = 6 (P = 0.46); I² = 0%		
Test for overall effect: Z = 2.86 (P = 0.004)		
**GL:MD solution**		
Jarvis et al. 1999 [85]	2.1%	0.08 [-0.79, 0.96]
**Subtotal (95% CI)**	**2.1%**	**0.08 [-0.79, 0.96]**
Heterogeneity: Not applicable		
Test for overall effect: Z = 0.19 (P = 0.85)		
**MD:FRU solution**		
McGawley et al. 2012 [100]	2.0%	0.40 [-0.48, 1.29]
Oosthuyse et al. 2015a i [115]	1.9%	0.07 [-0.86, 0.99]
Oosthuyse et al. 2015b [116]	1.7%	0.15 [-0.83, 1.14]
Pettersson et al. 2019 [120]	2.5%	-0.06 [-0.86, 0.74]
Roberts et al. 2014 i [123]	1.7%	0.50 [-0.47, 1.48]
**Subtotal (95% CI)**	**9.7%**	**0.20 [-0.21, 0.60]**
Heterogeneity: Chi² = 1.07, df = 4 (P = 0.90); I² = 0%		
Test for overall effect: Z = 0.95 (P = 0.34)		
**MD:DEX solution**		
Murray et al. 1991 ii [107]	2.0%	0.40 [-0.49, 1.29]
Murray et al. 1991 iii [107]	2.0%	0.49 [-0.40, 1.38]
**Subtotal (95% CI)**	**4.0%**	**0.45 [-0.18, 1.08]**
Heterogeneity: Chi² = 0.02, df = 1 (P = 0.89); I² = 0%		
Test for overall effect: Z = 1.39 (P = 0.16)		
**MD:SUC solution**		
Tsintzas et al. 1995 i [134]	1.5%	0.13 [-0.92, 1.17]
**Subtotal (95% CI)**	**1.5%**	**0.13 [-0.92, 1.17]**
Heterogeneity: Not applicable		
Test for overall effect: Z = 0.23 (P = 0.81)		
**GL:MD:FRU solution**		
Fulco et al. 2007 i [75]	1.4%	0.58 [-0.48, 1.64]
Fulco et al. 2007 ii [75]	1.5%	0.43 [-0.62, 1.48]
Millard-Stafford et al. 2005 ii [102]	2.0%	0.62 [-0.29, 1.52]
Tsintzas et al. 1995 ii [134]	1.4%	0.31 [-0.75, 1.36]
**Subtotal (95% CI)**	**6.3%**	**0.50 [-0.01, 1.00]**
Heterogeneity: Chi² = 0.23, df = 3 (P = 0.97); I² = 0%		
Test for overall effect: Z = 1.92 (P = 0.05)		
**GL:MD:DEX solution**		
Morris et al. 2003 [104]	1.8%	-0.31 [-1.24, 0.62]
**Subtotal (95% CI)**	**1.8%**	**-0.31 [-1.24, 0.62]**
Heterogeneity: Not applicable		
Test for overall effect: Z = 0.66 (P = 0.51)		
**GL:SUC:FRU solution**		
Campbell et al. 2008 [59]	3.3%	0.19 [-0.50, 0.89]
Osterberg et al. 2008 [117]	2.6%	0.60 [-0.19, 1.39]
Watson et al. 2012 i [140]	2.5%	0.19 [-0.61, 0.99]
Watson et al. 2012 ii [140]	2.4%	0.59 [-0.23, 1.41]
Watson et al. 2012 iii [140]	2.4%	0.60 [-0.22, 1.42]
Watson et al. 2012 iv [140]	2.4%	0.41 [-0.40, 1.22]
Watson et al. 2012 v [140]	2.4%	0.41 [-0.40, 1.22]
Watson et al. 2012 vi [140]	2.4%	0.63 [-0.19, 1.46]
**Subtotal (95% CI)**	**20.3%**	**0.44 [0.16, 0.72]**
Heterogeneity: Chi² = 1.50, df = 7 (P = 0.98); I² = 0%		
Test for overall effect: Z = 3.08 (P = 0.002)		
**GL:MD:MAL:Saccharides solution**		
Tsintzas et al. 1996a [135]	1.5%	0.89 [-0.16, 1.93]
Tsintzas et al. 1996b i [136]	2.2%	0.49 [-0.36, 1.34]
**Subtotal (95% CI)**	**3.7%**	**0.65 [-0.01, 1.31]**
Heterogeneity: Chi² = 0.33, df = 1 (P = 0.56); I² = 0%		
Test for overall effect: Z = 1.93 (P = 0.05)		
**SUC:MD:IsoMAL**		
Funnell et al. 2017 i [76]	3.3%	-0.28 [-0.97, 0.42]
Funnell et al. 2017 ii [76]	3.3%	-0.23 [-0.92, 0.47]
**Subtotal (95% CI)**	**6.6%**	**-0.25 [-0.74, 0.24]**
Heterogeneity: Chi² = 0.01, df = 1 (P = 0.93); I² = 0%		
Test for overall effect: Z = 1.00 (P = 0.32)		
**Unclear source CHO solution**		
Anastasiou et al. 2004 iii [50]	1.8%	1.01 [0.07, 1.96]
Tsintzas et al. 1993 [133]	1.5%	0.14 [-0.91, 1.19]
Williams et al. 1990 i [142]	2.1%	0.27 [-0.60, 1.14]
Williams et al. 1990 ii [142]	2.1%	0.18 [-0.68, 1.05]
**Subtotal (95% CI)**	**7.5%**	**0.40 [-0.06, 0.86]**
Heterogeneity: Chi² = 2.19, df = 3 (P = 0.53); I² = 0%		
Test for overall effect: Z = 1.69 (P = 0.09)		
**Total (95% CI)**	**100.0%**	**0.44 [0.32, 0.57]**
Heterogeneity: Chi² = 53.60, df = 49 (P = 0.30); I² = 9%		
Test for overall effect: Z = 6.88 (P < 0.00001)		
Test for subgroup differences: Chi² = 20.08, df = 11 (P = 0.04), I² = 45.2%		

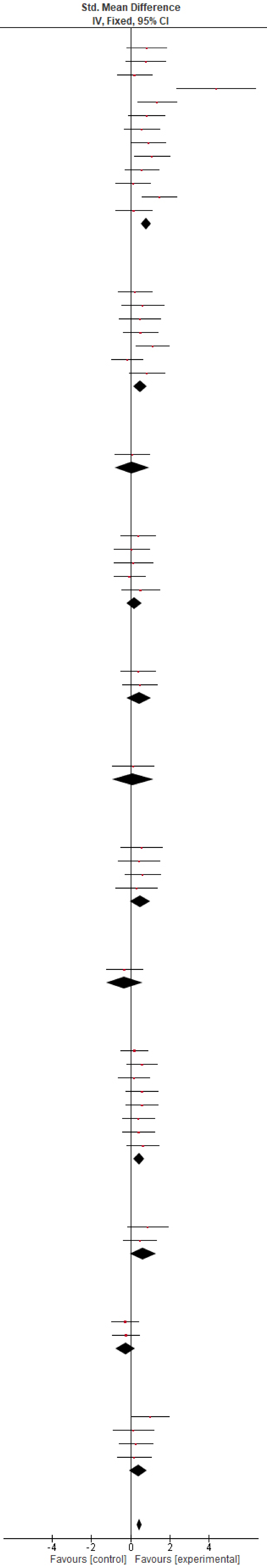

Figure S14: Forest plot shows the effects of experimental carbohydrate supplementation as compared to control on exercise outcome for 50 interventions. Subgroup analyses show the results with regards to supplementation in twelve MTC groups (GL:FRU, GL:SUC, GL:MD, MD:FRU, MD:DEX, MD:SUC, GL:MD:FRU, GL:MD:DEX, GL:SUC:FRU, GL:MD:MAL:Saccharides, SUC:MD:IsoMAL, Unclear CHO substances mixture). The black diamond symbol at the subgroups and at the bottom of the figure represents the standardized mean difference with the 95% confidence intervals for all interventions following fixed effects meta-analyses. Studies or trials that provided not sufficient data for subgroup classification were not included. Abbreviations: CHO, carbohydrate, CI, confidence interval; DEX, dextrose; FRU, fructose; GAL, galactose; GL, glucose; IV, inverse variance; MD, maltodextrin; MAL, maltose; MTC, multiple transportable carbohydrate; SD, standard deviation; Std, standardized; SUC, sucrose; i-vi denote different in-tervention arms (trials) within the same study

Study or Subgroup	Weight	Std. Mean Difference IV, Random, 95% CI
**CHO supplementation in Cool enviroment (<18°C)**		
Jarvis et al. 1999 [85]	1.0%	0.08 [-0.79, 0.96]
McGawley et al. 2012 [100]	1.0%	0.40 [-0.48, 1.29]
Murray et al. 1991 i [107]	0.9%	0.60 [-0.30, 1.50]
Murray et al. 1991 ii [107]	1.0%	0.40 [-0.49, 1.29]
Murray et al. 1991 iii [107]	1.0%	0.49 [-0.40, 1.38]
Pettersson et al. 2019 [120]	1.1%	-0.06 [-0.86, 0.74]
Rollo & Williams 2009 [126]	0.8%	0.28 [-0.71, 1.26]
Rollo & Williams 2010 [127]	1.0%	-0.06 [-0.93, 0.82]
Watson et al. 2012 i [140]	1.1%	0.19 [-0.61, 0.99]
Watson et al. 2012 ii [140]	1.1%	0.59 [-0.23, 1.41]
Watson et al. 2012 iii [140]	1.1%	0.60 [-0.22, 1.42]
**Subtotal (95% CI)**	**11.0%**	**0.32 [0.06, 0.58]**
Heterogeneity: Tau² = 0.00; Chi² = 3.40, df = 10 (P = 0.97); I² = 0%		
Test for overall effect: Z = 2.38 (P = 0.02)		
**CHO supplementation Neutral enviroment (18-26°C)**		
Acker-Hewitt et al. 2012 [47]	1.0%	0.09 [-0.79, 0.97]
Alghannam 2011 [48]	0.5%	1.67 [0.28, 3.07]
Angus et al. 2000 [51]	0.7%	1.23 [0.13, 2.33]
Bailey et al. 2000 i [52]	0.9%	0.57 [-0.38, 1.52]
Bailey et al. 2000 ii [52]	0.9%	0.34 [-0.59, 1.27]
Bishop et al. 2001 [56]	0.8%	0.89 [-0.09, 1.87]
Burgess et al. 1991 [57]	0.9%	0.18 [-0.74, 1.11]
Campbell et al. 2008 [59]	1.3%	0.19 [-0.50, 0.89]
Cole et al. 1993 i [64]	1.0%	0.23 [-0.65, 1.11]
Cole et al. 1993 ii [64]	1.0%	0.22 [-0.66, 1.10]
Cole et al. 1993 iii [64]	1.0%	0.21 [-0.67, 1.09]
Cole et al. 2018 [65]	0.5%	3.96 [2.62, 5.30]
Currell et al. 2008 i [68]	0.5%	2.55 [1.14, 3.97]
Currell et al. 2008 ii [68]	0.2%	4.37 [2.37, 6.37]
Desbrow et al. 2004 [70]	0.9%	0.02 [-0.90, 0.95]
Duhamel et al. 2007 [71]	1.2%	0.85 [0.10, 1.60]
El-Sayed et al. 1995 [72]	0.9%	0.57 [-0.38, 1.52]
El-Sayed et al. 1997 [73]	0.8%	0.16 [-0.82, 1.14]
Finger et al. 2018 [74]	1.2%	0.04 [-0.73, 0.81]
Gui et al. 2017 [80]	1.0%	0.47 [-0.38, 1.32]
Heesch et al. 2014 i [81]	0.8%	0.28 [-0.71, 1.26]
Heesch et al. 2014 ii [81]	0.8%	0.17 [-0.81, 1.15]
Heesch et al. 2014 iii [81]	0.8%	0.31 [-0.68, 1.30]
Jeukendrup et al. 1997 [86]	1.4%	0.54 [-0.11, 1.19]
Khanna & Manna 2005 [89]	0.7%	2.10 [0.96, 3.24]
Kingwell et al. 1989 [92]	0.9%	0.53 [-0.42, 1.47]
Learsi et al. 2019 i [93]	0.8%	0.89 [-0.09, 1.87]
Learsi et al. 2019 ii [93]	0.6%	2.31 [1.05, 3.57]
Lugo et al. 1993 [94]	0.8%	0.88 [-0.10, 1.86]
Martínez-Lagunas et al. 2010 [95]	1.1%	0.69 [-0.14, 1.52]
McConell et al. 1996 [97]	0.7%	1.02 [-0.04, 2.08]
McConell et al. 2000 [99]	1.2%	-0.08 [-0.85, 0.69]
Millard-Stafford et al. 2005 i [102]	1.0%	0.50 [-0.39, 1.40]
Millard-Stafford et al. 2005 ii [102]	0.9%	0.62 [-0.29, 1.52]
Nassis et al. 1998 [110]	0.9%	-0.10 [-1.02, 0.83]
Newell et al. 2015 i [111]	1.5%	0.34 [-0.28, 0.97]
Newell et al. 2015 ii [111]	1.5%	0.59 [-0.05, 1.22]
Newell et al. 2015 iii [111]	1.5%	0.62 [-0.02, 1.25]
Nicholas et al. 1995 [112]	0.9%	0.55 [-0.40, 1.49]
Oosthuyse et al. 2015a i [115]	0.9%	0.07 [-0.86, 0.99]
Oosthuyse et al. 2015a ii [115]	0.9%	-0.31 [-1.24, 0.62]
Oosthuyse et al. 2015b [116]	0.8%	0.15 [-0.83, 1.14]
Osterberg et al. 2008 [117]	1.1%	0.60 [-0.19, 1.39]
O'Hara et al. 2014 i [118]	1.0%	0.34 [-0.54, 1.22]
O'Hara et al. 2014 ii [118]	1.0%	-0.25 [-1.13, 0.63]
O'Neal et al. 2013 [119]	2.0%	0.10 [-0.36, 0.56]
Pottier et al. 2010 [121]	1.1%	-0.16 [-0.96, 0.64]
Roberts et al. 2014 i [123]	0.9%	0.50 [-0.47, 1.48]
Roberts et al. 2014 ii [123]	0.9%	-0.06 [-1.02, 0.90]
Robson-Ansley et al. 2011 [125]	0.9%	0.16 [-0.76, 1.09]
Rollo et al. 2011 [128]	1.0%	0.40 [-0.49, 1.29]
Smith et al. 2010 i [129]	1.1%	0.41 [-0.40, 1.22]
Smith et al. 2010 ii [129]	1.1%	0.49 [-0.32, 1.31]
Smith et al. 2010 iii [129]	1.1%	0.65 [-0.18, 1.47]
Sun et al. 2015 [131]	0.8%	0.62 [-0.39, 1.63]
Tsintzas et al. 1995 i [134]	0.8%	0.13 [-0.92, 1.17]
Tsintzas et al. 1995 ii [134]	0.7%	0.31 [-0.75, 1.36]
Tsintzas et al. 1996a [135]	0.8%	0.89 [-0.16, 1.93]
Tsintzas et al. 1996b i [136]	1.0%	0.49 [-0.36, 1.34]
Tsintzas et al. 1996b ii [136]	1.0%	0.37 [-0.48, 1.21]
Utter et al. 2002 [137]	2.2%	0.04 [-0.36, 0.43]
Van Essen et al. 2006 [138]	1.0%	0.19 [-0.69, 1.07]
Wilber et al. 1992 [141]	0.9%	0.85 [-0.08, 1.77]
Williams et al. 1990 i [142]	1.0%	0.27 [-0.60, 1.14]
Williams et al. 1990 ii [142]	1.0%	0.18 [-0.68, 1.05]
**Subtotal (95% CI)**	**63.2%**	**0.47 [0.33, 0.61]**
Heterogeneity: Tau² = 0.12; Chi² = 103.98, df = 64 (P = 0.001); I² = 38%		
Test for overall effect: Z = 6.68 (P < 0.00001)		
**CHO supplementation in Heat enviroment (> 26°C)**		
Anastasiou et al. 2004 i [50]	0.9%	0.83 [-0.10, 1.75]
Anastasiou et al. 2004 ii [50]	1.0%	0.31 [-0.57, 1.20]
Anastasiou et al. 2004 iii [50]	0.9%	1.01 [0.07, 1.96]
Baldassarre et al. 2021 [53]	0.8%	0.83 [-0.21, 1.86]
Below et al. 1995 [55]	0.8%	0.77 [-0.26, 1.80]
Byrne et al. 2005 [58]	1.2%	0.22 [-0.52, 0.97]
Carter et al. 2003 i [60]	0.7%	0.70 [-0.39, 1.79]
Carter et al. 2003 ii [60]	0.8%	0.38 [-0.61, 1.37]
Carter et al. 2005 i [61]	0.8%	0.49 [-0.51, 1.49]
Carter et al. 2005 ii [61]	0.8%	0.44 [-0.56, 1.43]
Clarke et al. 2011 [63]	0.6%	2.90 [1.70, 4.10]
Cureton et al. 2007 [67]	1.3%	0.35 [-0.35, 1.05]
Ganio et al. 2010 [77]	1.2%	0.42 [-0.33, 1.17]
Millard-Stafford et al. 1990 [101]	1.0%	0.14 [-0.74, 1.02]
Millard-Stafford et al. 2007 [103]	1.3%	0.34 [-0.36, 1.04]
Morris et al. 2003 [104]	0.9%	-0.31 [-1.24, 0.62]
Murray et al. 1987 i [105]	1.1%	0.63 [-0.16, 1.42]
Murray et al. 1987 ii [105]	1.0%	1.13 [0.29, 1.97]
Murray et al. 1987 iii [105]	1.0%	1.49 [0.60, 2.37]
Murray et al. 1989 i [106]	1.1%	0.57 [-0.25, 1.39]
Murray et al. 1989 ii [106]	1.1%	0.30 [-0.50, 1.11]
Murray et al. 1989 iii [106]	1.1%	0.05 [-0.75, 0.85]
Nassif et al. 2014 [109]	0.9%	-0.66 [-1.56, 0.25]
Watson et al. 2012 iv [140]	1.1%	0.41 [-0.40, 1.22]
Watson et al. 2012 v [140]	1.1%	0.41 [-0.40, 1.22]
Watson et al. 2012 vi [140]	1.1%	0.63 [-0.19, 1.46]
**Subtotal (95% CI)**	**25.8%**	**0.52 [0.31, 0.73]**
Heterogeneity: Tau² = 0.10; Chi² = 37.45, df = 25 (P = 0.05); I² = 33%		
Test for overall effect: Z = 4.90 (P < 0.00001)		
**Total (95% CI)**	**100.0%**	**0.46 [0.36, 0.57]**
Heterogeneity: Tau² = 0.09; Chi² = 146.36, df = 101 (P = 0.002); I² = 31%		
Test for overall effect: Z = 8.71 (P < 0.00001)		
Test for subgroup differences: Chi² = 1.54, df = 2 (P = 0.46), I² = 0%		

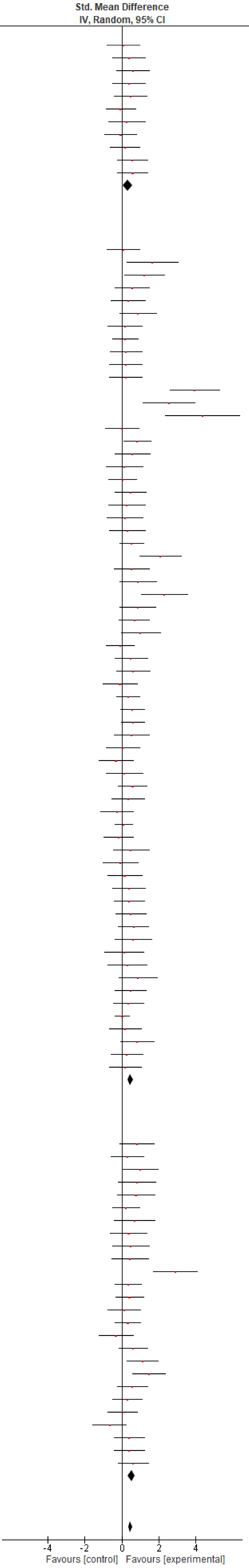

Figure S15: Forest plot shows the effects of experimental carbohydrate supplementation as compared to control on exercise outcome for 102 interventions. Subgroup analyses show the results with regard to ambient conditions in two thermal condition groups [Cool (< 18°C), Neutral (18-26°C), Heat (> 26°C)]. The black diamond symbol at the subgroups and at the bottom of the figure represents the standardized mean difference with the 95% confidence intervals for all interventions following random-effect meta-analyses. Studies or trials that provided insufficient data for subgroup classification were not included. Abbreviations: CHO, carbohydrate; CI, confidence interval; IV, inverse variance; SD, standard deviation; Std, standardized; i-vi denote different in-tervention arms (trials) within the same study

Figure S16: Funnel plot of comparison: I. in all studies (and trials); and II.-VII. in classes of different subgroups analysis

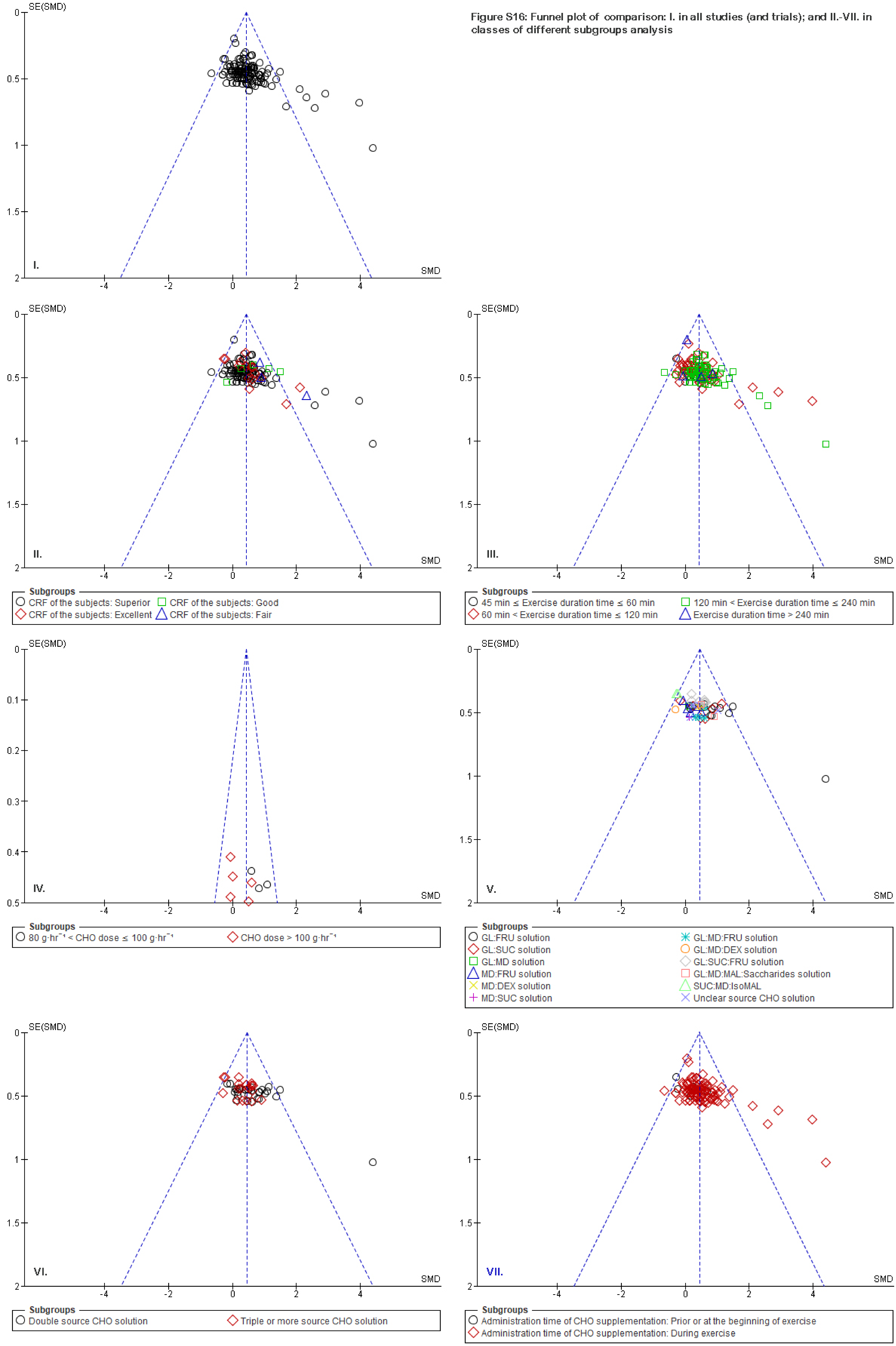

Supplement: Supplementary file 1 [file nutrients-13-04223-s001.zip › nutrients-1438372-supplementary.pdf]
